# Abstracts from the International Conference Diversity Interventions 2022

**DOI:** 10.1186/s12919-023-00275-w

**Published:** 2023-10-26

## Edited by Pavel V Ovseiko^1*^ and Mei Leow^2*^

### ^1^University of Oxford, Oxford, United Kingdom; ^2^Science in Australia Gender Equity (SAGE) Limited, Canberra, Australia

**Correspondence:** Pavel V Ovseiko (pavel.ovseiko@rdm.ox.ac.uk); Mei Leow (mei.leow@sciencegenderequity.org.au)

*BMC Proceedings 2023*, **17(15)**

**Introduction**

Diversity Interventions is an international conference dedicated to promoting advances in gender equality, diversity and inclusion. The conference has emerged from the collaboration between the organisations that use the Athena Swan framework across the globe to advance gender equality, diversity and inclusion within higher education and research [https://www.advance-he.ac.uk/equality-charters/international-charters]. The conference serves as a platform for equality, diversity and inclusion professionals, researchers and advocates from across the globe to share best practice, discuss emerging innovations and exchange personal experiences in designing, implementing and evaluating interventions and action plans.

Due to the COVID-19 travel restrictions and time differences across continents, Diversity Interventions 2022 was held as two events with the joint call for abstracts. “*Diversity Interventions 2022 SAGE: Systemic approaches that work*” was organised and hosted by Science in Australia Gender Equity (SAGE) in Canberra, Australia on 5–6 April 2022 as a virtual conference [https://sciencegenderequity.org.au/event/diversity-interventions-2022-sage-systemic-approaches-that-work/]. “*Diversity Interventions 2022: Towards a Science and Profession of Athena Swan*” was organised and hosted by Advance HE in partnership with the University of Oxford on 7–8 April 2022 as a hybrid conference [https://diversityinterventions.org/].

The joint call for abstracts focused on the following topics:

1. *Gender equality action plans*. How institutions or departments design, implement and evaluate gender equality action plans.
2. *Innovative interventions.* What works (and what doesn’t) in addressing underrepresentation in leadership/particular disciplines/key transition points.
3. *Translating lessons and successes.* Implementing what works (or learnings from the activities/actions that didn’t) outside the original setting and/or scaling up from one part of the organisation to others, or from one sector to another.
4. *Professional development of equality, diversity and inclusion (EDI) practitioners.* What knowledge, skills and experiences are required by EDI practitioners and how to provide these.
5. *Institutional and departmental maturity.* Enterprise-wide and departmental approaches that embed gender equality and diversity as business imperatives, show a sophisticated understanding of intersectionality and set the course for lasting change.
6. *Meaningful partnerships and co-design.* How institutions and departments are working with or being led by affected groups to change systems.
7. *Impact of COVID-19* on EDI, flexible work and the future of work, and how to adapt EDI interventions and action plans to the new normal.

**Acknowledgements**

The conference received funding from Science in Australia Gender Equity (SAGE) Limited; Advance HE; The John Fell Fund, University of Oxford; The van Houten Fund, University of Oxford; and the European Union’s Horizon 2020 research and innovation programme under grant agreement no. 872396 ALLINTERACT (Widening and diversifying citizen engagement in science). PVO is supported by the NIHR Oxford Biomedical Research Centre (BRC), grant BRC-1215-20008 to the Oxford University Hospitals NHS Foundation Trust and the University of Oxford.

## A1 Male allyship in institutional STEMM gender equity initiatives

### Meredith Nash^1*^, Ruby Grant^2^, Robyn Moore^1^, Tania Winzenberg^3^

#### ^1^University of Tasmania, Hobart, Australia; ^2^University of Tasmania, Launceston, Australia; ^3^Menzies Institute for Medical Research, Hobart, Australia

##### **Correspondence:** Meredith Nash (hello@meredithnash.com)

*BMC Proceedings 2023*, **17(15):**A1

For a full report published elsewhere, please see: Nash M, Grant R, Moore R, Winzenberg T. Male allyship in institutional STEMM gender equity initiatives. PLOS ONE 2021;16(3): e0248373. 10.1371/journal.pone.0248373

## A2 A sustainable Program to Support Early Academic Careers (PEAC)

### Julia Richardson, Dorothy Wardale, Julianne J Reid

#### Curtin University, Perth, Australia

##### **Correspondence:** Dorothy Wardale (d.wardale@curtin.edu.au)

*BMC Proceedings 2023*, **17(15):**A2

**Background**

From 2020 to 2021, Curtin University designed and delivered a high-impact Program to support Early Academic Careers (PEAC). The impetus for the program was in part to demonstrate our commitment as signatories to Athena Swan and to respond to feedback from early career academics (ECAs) signalling the need for a targeted program of learning, development and networking to support women’s careers including: provision of easier access to university information; career support; mentoring; managing competing work priorities; and cross-institutional interaction.

**Methods**

Twenty-four women from across Curtin were nominated by their Head of School to participate in the Program with the following learning outcomes: professional strength building; expanding cross disciplinary networks and understanding of institutional support systems; deepening understanding of the higher education landscape; and developing a three-year career plan. After widespread consultation by the leaders of the Program the final design comprised five workshops (9am to 3pm to accommodate personal commitments), mentorship, peer networking and executive support. The initial 6-month structure was adjusted to accommodate restrictions from the global pandemic stretching to just under a year. The design also met our commitment to minimising information overload, encouraging individual reflection and learning and accommodating participants’ work and non-work commitments.

**Results**

An evaluation survey indicated explicit and immediate impact including increased understanding of university structures and processes, enhanced professional and interpersonal skills and a stronger sense of career ownership: “*[it] gave me a kick and reminded me to keep fighting for my career*”; ”*The ability to network and meet others outside of my immediate field and area has proved valuable to expanding my professional reach*”. Of the twenty participants who completed the Program, seven received an academic promotion and a further two were promoted to a higher rank in other universities. Many of those who had applied for promotion indicated that they would not have done so without the Program: “*The PEAC program gave me the tools and confidence to apply for promotion … and I was successful*”; “*During this process I applied for promotion because of the message to step up and believe in myself… and I got it!*” Other measures of success included teaching awards (2), research awards (2), leadership and innovation awards (2), and becoming an academic discipline lead (1). While the global pandemic introduced many challenges in the design and delivery of the Program, identification and availability of mentoring partners was challenging. Scheduling meetings between mentors and mentees proved especially difficult and some participants indicated they would have preferred to select their own mentor.

**Conclusions**

The overwhelming success of PEAC has set a path for future progress at Curtin where we are expanding access to other cohorts. PEAC is now being refined and delivered again by new facilitators and speakers and offered to both men and women ECAs. All materials and learning outcomes from design and delivery of the Program are centrally stored to ensure a sustainable suite of offerings. As a global university we are also looking to expand the program to our global campuses.

## A3 Lifting diversity and inclusion in economics: how the Australian Women in Economics Network put the evidence into action

### Duygu Yengin^1*^, Leonora Risse^2^, Rebecca Cassells^3,4^, Danielle Wood^5^

#### ^1^University of Adelaide, Adelaide, Australia; ^2^RMIT, Melbourne, Australia; ^3^Commonwealth Treasury, Canberra, Australia; ^4^Bankwest Curtin Economics Centre (BCEC), Canberra, Australia; ^5^Grattan Institute, Melbourne, Australia

##### **Correspondence:** Duygu Yengin (duygu.yengin@adelaide.edu.au)

*BMC Proceedings 2023*, **17(15):**A3

For a full report published elsewhere, please see: Cassells R, Risse L, Wood D, Yengin D Lifting diversity and inclusion in Economics: how the Australian Women in Economics Network put the evidence into action. Econ Pap. 2023; 42:1-29. 10.1111/1759-3441.12367

## A4 Pandemic productivity in academia: using Ecological Momentary Assessment to explore the impact of COVID-19 on research productivity

### Roxanna N Pebdani, Adriana Zeidan, Lee-Fay Low, Andrew Baillie

#### The University of Sydney, Sydney, Australia

##### **Correspondence:** Roxanna N Pebdani (roxanna.pebdani@sydney.edu.au)

*BMC Proceedings 2023*, **17(15):**A4

For a full report published elsewhere, please see: Pebdani RN, Zeidan A, Low LF, Baillie A. Pandemic productivity in academia: using ecological momentary assessment to explore the impact of COVID-19 on research productivity. High Educ Res Dev. 2023;42(4):937-53. https://doi.org/10.1080/07294360.2022.2128075

## A5 Challenges and strategies from the perspective of a culturally diverse staff network

### Kumudika de Silva, Fernanda Penaloza, Zakia Hossain, Dieter Hochuli, Naomi Koh Belic, Sanetta Du Toit, Rahena Akhter, Tracey Tsang, Corinne Caillaud

#### University of Sydney, Sydney, Australia

##### **Correspondence:** Kumudika de Silva (kumudika.desilva@uts.edu.au)

*BMC Proceedings 2023*, **17(15):**A5

The Mosaic network at the University of Sydney was created in 2018, at a time when multicultural diversity was not a focus within the ‘diversity’ agenda in the Australian Higher Education sector. It functions as a unique combination of a grassroots initiative with executive support and aims to challenge the status quo about experiences, support and progression of culturally and linguistically diverse (CALD) colleagues.

In creating Mosaic and giving it robust foundations, the network leads sought leadership buy-in and engaged with colleagues. Some of the themes that emerged at an initial all-staff workshop were that Mosaic should aim to increase visibility and influence the experiences of CALD staff at the university. Internalised stereotypes (for example, the negative or positive association-perception of certain groups) and lack of social acceptance were highlighted by workshop participants as inhibitors to a successful culturally inclusive workplace while the role Mosaic could play was envisaged as providing networking opportunities, increasing awareness and influencing governance.

Mosaic follows a shared model of leadership with the Steering Committee led by three co-Chairs and is supported by an Executive Sponsor. The network collaborates with other like-minded groups to organise workshops on topics relevant to our communities (racism, microaggression), create opportunities to shift the workplace culture and create safe spaces in which colleagues share experiences. Mosaic’s Say My Name campaign which aims to help others pronounce a person’s name and signal additional languages (Figure 1) has been widely accepted by colleagues within and beyond the university.

Important challenges the network encounters are likely to be familiar to others engaged in creating diversity interventions in Australia. These include 1) engaging beyond the ‘CALD bubble’, 2) counting and examining culture and 3) delegating/diversifying the responsibility for creating change.

The responsibility of identifying and resolving equity-related issues continues to fall on individuals from specific minority groups. In the case of Mosaic, there is limited buy-in from voices or perspectives outside the CALD community and there is a gendered disinterest in being involved in equity initiatives. As an attempt to resonate with people outside the interest groups they support Mosaic has recently partnered with other stakeholders to develop an anti-racism pledge which encourages staff to listen, pause, reflect, and commit to take actions to create more inclusive spaces within teaching, research and other workplace practices.

In the 3 years that Mosaic has been operational, it has become an important resource for staff by creating safe spaces which allow CALD staff to share their experiences. However, a real challenge is how conversations within this group can actually guide action to address and overcome the obstacles we face: in other words, how do we go from being a support group to become an action group? By creating a direct line of communication to senior executives at the university via the Executive Sponsor, Mosaic now has opportunities to be heard at the highest levels within our institution.

**Fig. 1 (abstract A5).**
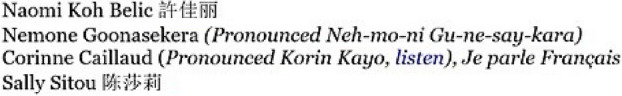
Example for the Say My Name campaign.

## A6 Promoting gender equity within the academic promotions process

### Lesley Hughes

#### Macquarie University, Sydney, Australia

##### **Correspondence:** Lesley Hughes (lesley.hughes@mq.edu.au)

*BMC Proceedings 2023*, **17(15):**A6

Macquarie University has implemented and evaluated an innovative new academic promotions scheme that works in parallel with our Gender Equity Strategy, broadening the scope of recognised achievement and improving the representation of women at higher academic levels.

Most academic promotion systems worldwide have remained essentially unchanged for decades, often failing to capture the complexity of modern academic lives.

Our new approach, with a focus of streamlining both the application and assessment process, uses criteria for promotion based on Ernest Boyer’s four areas of academic scholarship: Discovery, Integration, Teaching and Application, with an additional criterion of Leadership and Citizenship.

Our overarching aim was to design a strengths-based assessment, recognising the diversity of career pathways and academic work. The scheme also aims to provide greater transparency of decision-making, increased involvement from faculties and an alignment of recruitment and promotion standards.

Applicants self-assess on a points basis (assigning 0-3 points to each of the five categories), guided by an extensive list of evidence indicators, with successively more rigorous thresholds of achievement required for promotion to higher levels.

The deliberately flexible scoring system allows applicants to spread points across the four Boyer categories (with mandatory elements in Leadership and Citizenship). Minimum points are required for promotion as well as outstanding achievements in at least one category. Faculty-level promotions committees then assess an applicant’s case, ensuring justification based on evidence provided, and external validation (where possible).

Since the new model was implemented four years ago, applications have increased by nearly 70% compared to the average for the previous five years, with the proportion of women applicants increasing proportionately more (87%) than men (49%).

A comparison of self-assessment and committee scores showed self-assessment scores were higher for both women and men before interview. After interviews, these differences were reduced slightly for women, indicating women were somewhat more advantaged by having the opportunity to make their case for promotion orally and to respond to questions.

An evaluation found 93% of promotion committee members agreed or strongly agreed that the new scheme was strength-based and acknowledged the diversity of academic work. Qualitative comments were also positive: “*I loved the flexibility to argue your case for promotion - gone are the days of research output being the sole indicator of achievement! In the current tertiary climate, it is imperative to acknowledge the diverse array of skills and contributions required of academics, and that each of us will have unique profiles that are difficult to compare 1:1 with our peers.*”

The new scheme has had broad support across the university community, is seen as fairer and more inclusive, and is contributing to greater gender balance across academic levels. It is our hope that this promotions model will continue to be a core enabler for improving morale, job satisfaction, performance excellence and institutional growth.

## A7 Responsibilities when teaching critical race and whiteness studies

### Holly Randell-Moon

#### Charles Sturt University, Dubbo, Australia

##### **Correspondence:** Holly Randell-Moon (hrandell-moon@csu.edu.au)

*BMC Proceedings 2023*, **17(15):**A7

In this presentation, I reflect on my practices in teaching critical race and whiteness studies to undergraduate and postgraduate students over the past fifteen years in higher education. I discuss some of the key theoretical insights of critical race and whiteness studies and outline some strategies for negotiating white privilege and white possession in professional training and education contexts. I situate myself in these teaching practices as a white and non-Indigenous academic and critically examine what facilitating anti-racist teaching means in the relational space of learning. In recognising how non-Indigenous peoples view me as an ally in deficit discourse regarding Indigenous peoples, I can work in a pedagogical space to contest these views and the emotional responses to critical reflection and histories of colonial racism. Promoting racial literacy through critical race and whiteness studies is one contribution to decolonising the knowledges that circulate in public institutions and discourse regarding the nation-state, history, and national identity. Non-Indigenous teachers such as myself have a pedagogical responsibility to work with their communities in challenging the cultural norms of white possession and the privileges we derive from it.

## A8 Knowledge, skills and experiences developed to be an effective university EDI practitioner

### Shamika Almeida, Jenny Fisher, Mark Freeman

#### University of Wollongong, Wollongong, Australia

##### **Correspondence:** Shamika Almeida (shamika@uow.edu.au)

*BMC Proceedings 2023*, **17(15):**A8

This abstract reports on the key skills, knowledge, and experiences we believe we possess as Equity, Diversity and Inclusion (EDI) practitioners at an Australian Higher Education Provider and how these have had an enduring impact on our co-workers. We are three Associate Deans (AD) of EDI across the Science, Technology, Engineering and Mathematics (STEM) and Business and Law faculties. Our role is limited to 40% of our time, and the rest of the time, we continue with our usual academic activities (teaching and research). Our positions are strategic and not operational, and we have been in our appointments for between 7 and 18 months. Being an AD-EDI means we work to create an inclusive work environment for all staff. We recognise that some staff (and students) may not be ready for change, and our actions might not please everyone.

We each have unique lived experiences related to both inequity and privilege. One of us is the youngest sister of three siblings, the first in the family to complete university and a first-generation migrant woman of colour with adult children. One is a white woman in STEM who is the only child from a middle-class family and is a first-generation migrant who arrived in Australia after completing her PhD at a globally prestigious university. One is a born and bred white Australian man who humbly states that he comes from a privileged background but is first in the family and a new dad. We are very different in our personalities and experiences. However, we share our passion for celebrating diversity and championing equity, our willingness to be uncomfortable with change, and our appreciation for ongoing learning to create an inclusive and care-based workplace.

We also have the skill set to be curious and respectfully ask questions and acknowledge our ignorance of what we do not know. For example, how do we use multiple pronouns and acknowledge Country in a meaningful and authentic way? How do we ask someone about pronouncing their names and their cultural values? We learn and disseminate information on novel concepts to many, such as intersectionality: the compounding impact of multiple factors (e.g. cultural background, disability status, gender, sexuality and socioeconomic background).

As a collective of AD-EDIs, we realise that we need to divide and conquer to do our best while also caring for our wellbeing. To do this, we respect each other and have built trust. Thus, we all do not need to be involved in every project; collectively, we nominate a lead to action specific activities or represent us on certain committees. We share our resources to learn from each other and reduce duplication by having regular catch-ups where we have reflective conversations. We empower others to be EDI advocates, creating a ripple effect and recognising the need for inclusion at all different levels of the organisation, as people are influenced by their closest links.

We conclude by stating that our lived experiences, skillset, and collective approach to EDI impacts the effectiveness of the EDI team.

## A9 Gender in policy language

### Robin C Ladwig

#### University of Canberra, Canberra, Australia

##### **Correspondence:** Robin C Ladwig (robin.ladwig@canberra.edu.au)

*BMC Proceedings 2023*, **17(15):**A9

The representation of gender identities through language is a decades-long conversation about the invisibility of women due to the generic masculine or gender neutrality. In recent years, the debate between feminist and transgender and gender diverse scholars and activists has prospered. Nevertheless, transgender and gender diverse identities have been neglected or written out of policies by applying cis-normative gender binary language limited to women and men.

The workshop is run as a respectful space where people can learn and co-create in good faith, with the objective of opening up the organisation to diverse gendered experiences. The aim is to build an understanding of the complexities and possibilities of organisation policy in navigating people’s rights and embodied experiences of work. The practice-orientated workshop explores the application of the queering approach [1] including feminist perspectives [2] to increase gender diversity and equity in organisational policies.

This workshop draws from my research project concerning work experiences and career development of transgender and gender diverse individuals in Australia [3]. Participants are asked to explore the implications of three approaches to increasing gender diversity and equity through language: gender inclusivity or multiplicity, gender neutrality, and gender distinctiveness. The first approach is gender inclusivity or multiplicity which embraces the naming of various gender identities plus applying gendered language beyond the gender binary to include considerations of transgender and gender diverse lived realities in organisational policies. The second approach is gender neutrality which refers to the exclusion of specific gender markers to avoid gender-stereotypical bias and stigmatisation. The third approach is gender distinctiveness which refers to explicit labelling of gendered lived experiences such as maternity or menopause for women or gender affirmation for transgender and gender diverse individuals.

The discussion is limited to explicitly gendered terminology and does not consider feminine- or masculine-themed words [4]. Participants are encouraged to analyse policy under a gender diverse lens and propose situational solutions to increase the inclusion of transgender and gender diverse identities.

**References**

1. Christensen JF. Queer organising and performativity: towards a norm-critical conceptualisation of organisational intersectionality. Ephemera. 2018;18(1):103-30.
2. Acker J. Hierarchies, jobs, bodies: a theory of gendered organizations. Gend Soc. 1990;4(2):139-58.
3. Ladwig RC. Trans and gender diverse work experiences and career development in the Australian work environment. Gender and Sexuality at Work: A Multidisciplinary Research and Engagement Conference. 2022;2:43-7.
4. Tokarz RE, Mesfin T. Stereotyping ourselves: gendered language use in management and instruction library job advertisements. J Lbr Adm. 2021;61(3):301-11.

## A10 Fighting gender inequality in media education: in the classroom and on set

### Lucy A A Brown

#### London South Bank University, London, United Kingdom

##### **Correspondence:** Lucy A A Brown (lucybrown@lsbu.ac.uk)

*BMC Proceedings 2023*, **17(15):**A10

There is a startling lack of gender equality in the film and television industry despite data from the Higher Education Statistics Agency (HESA) indicating a gender balance on media university courses. Why are women not climbing the ladder to direct, write, produce, or take on other craft roles on the same scale as their male counterparts and what measures can film schools and universities put in place to combat this inequity? This presentation calls for the academy and industry to work collaboratively to end gender inequality in the media sector and shares best practice garnered from workshops, screenings, and discussions between students, practitioners, politicians, researchers and pedagogues put forward at the international Trailblazing Women on and off Screen Conference between 2017-21. It is solutions focused and proposes a set of gender equality action plans to better support female students and graduates who wish to work in the screen industries.

## A11 Improving the under-representation of women in senior roles in higher education

### Mark C Toner^1*^, Gunilla E Burrowes^2^

#### ^1^Gender Matters, Melbourne, Australia; ^2^Gender Matters, Newcastle, Australia

##### **Correspondence:** Mark C Toner (mctoner@tonner-assoc.com.au)

*BMC Proceedings 2023*, **17(15):**A11

Figure 1 shows the continuing under-representation at academic levels D and E across Australian Higher Education (HE) institutions.

There are a number of common myths about why there aren't more women in senior HE roles. These myths include: women are not as interested in senior roles as men are; women don't perform well in senior roles compared with men; there aren’t enough women available for these roles.

Some valid reasons for the under-representation are: for a variety of reasons, women don’t apply for these roles as much as men do; some men in charge favour men and discriminate against women in recruitment, promotion and in other decisions.

Why do these men discriminate against women? It could be either deliberate or unintentional, but a major factor is cognitive bias. As humans we are all biased, i.e. we form opinions and make decisions which do not accord with relevant facts or data.

Two common biases affecting recruitment and promotion decisions are: 1) In-group and Out-group Bias: we favour people with similar characteristics, interests or backgrounds to ourselves and are uncomfortable with and act to disadvantage others; 2) Halo Effect: we assume that because someone has some positive features or characteristics, they have other positive characteristics. The opposite is called the Horn Effect.

Unfortunately, there are many more biases relevant to recruitment and promotion. Biases can be unconscious or conscious; we are all subject to both types. Our unconscious mind is extremely powerful; it forms associations and opinions without our conscious knowledge, and when we make decisions, unconscious bias or conscious bias are usually present.

Bias mitigation training is very popular but there is little evidence it reduces bias, because it is hard to change unconscious associations; they can even remain in conflict with our conscious beliefs.

Rather than just documenting the under-representation of women in senior positions, it is important to design and implement a valid solution. Our recommendations for organisations are they: ensure that advertised roles are described in a gender-neutral way; ensure that appropriate policies and procedures are followed; organise discussions on ‘merit’ (a subjective concept susceptible to bias); organise removal of personal data for shortlisting candidates; identify and encourage suitable women to apply for positions and promotions.

Panel members or individuals making recruitment/promotion decisions should: ensure clear non-gendered judging criteria are used; take into account gender differences, e.g. awareness of stereotypes and self-estimation differences; understand the major biases relevant to recruitment and promotion decisions, their causes and their mitigation; if on a panel, constructively discuss their own and other members’ biases before and after making decisions, in a transparent process.

In conclusion, the first step in improving the number of women in senior HE roles is understanding the reasons for the problem. The second step is tackling these reasons with appropriate mitigation strategies. The third step is establishing best practice in recruitment and promotion decision-making, and demanding it is followed across the organisation.

**Fig. 1 (abstract A11).**
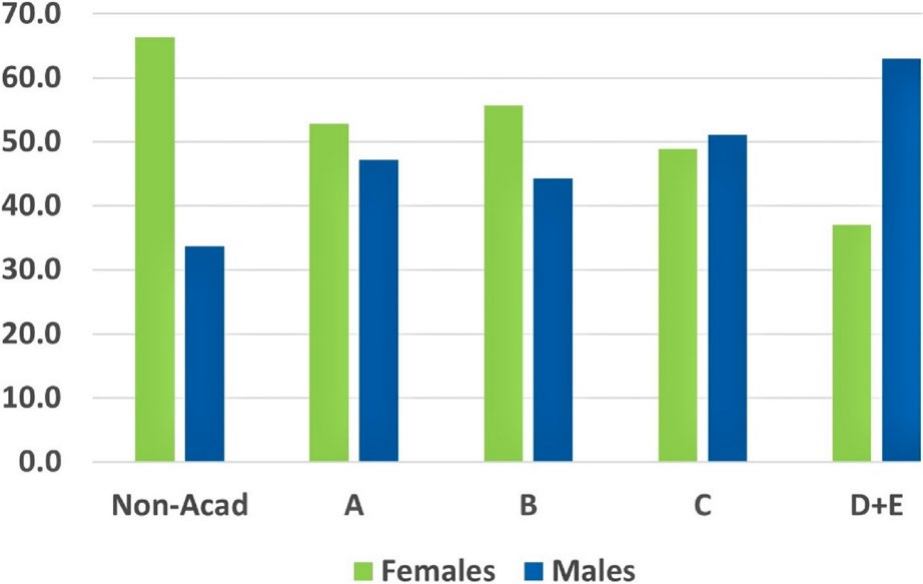
Percentage by gender in full-time and fractional full-time HE staff numbers in 2021 (source: Department of Education, Skills and Employment)

## A12 Visibilising mothering in the academy: (re)performing academic mothering in the transformative moment of COVID-19

### Emilee Gilbert^1^, Carla Pascoe-Leahy^2^

#### ^1^Western Sydney University, Sydney, Australia; ^2^Melbourne University, Melbourne, Australia

##### **Correspondence:** Emilee Gilbert (e.gilbert@westernsydney.edu.au)

*BMC Proceedings 2023*, **17(15):**A12

For a full report published elsewhere, please see: Gilbert E, Pascoe-Leahy C. Viewpoint: Visibilising care in the academy: (re)performing academic mothering in the transformative moment of COVID-19. Gend Hist 2023;35:340-61. https://doi.org/10.1111/1468-0424.12659

## A13 What works? Evaluating your equity program

### Isabelle Kingsley^1,2*^, Lisa Harvey-Smith^1^

#### ^1^Office of the Women in STEM Ambassador, Sydney, Australia; ^2^University of New South Wales, Sydney, Australia

##### **Correspondence:** Isabelle Kingsley (isabelle.kingsley@unsw.edu.au)

*BMC Proceedings 2023*, **17(15):**A13

If we are trying to create change towards a more diverse, equitable and inclusive world, then we need to understand what works and how to improve what doesn’t. Evaluation is a key part of any equity, diversity and inclusion (EDI) program. It allows you to understand if, how and to what extent your actions are creating change. This hands-on workshop provides practical advice to help you to evaluate your program. It will give you the tools to bring accountability, transparency, demonstrable impact and an evidence-based approach to EDI programs.

*What to expect:* In the first half of the workshop, Professor Lisa Harvey-Smith, the Australian Government's Women in STEM Ambassador [https://womeninstem.org.au/], will outline why we need to evaluate EDI programs. Then, Isabelle Kingsley — author of the National Evaluation Guide [https://womeninstem.org.au/national-evaluation-guide/] — will break evaluation down into a simple, 5-step approach. She will show you how to embed evaluation within your program from the very beginning. In the second half of the workshop, Isabelle will help you will complete your own evaluation plan using templates and resources from the National Evaluation Guide. The session will end with an open Q&A forum.

*Before the workshop:* Please familiarise yourself with the National Evaluation Guide and associated resources. Come ready with an EDI program to evaluate—it can be your own, someone else’s or a sample program [https://womeninstem.org.au/examples-of-program-evaluations/].

## A14 Monitoring Australia’s STEM gender divide

### Jaynell Vila

#### Department of Industry, Science and Resources, Canberra, Australia

##### **Correspondence:** Jaynell Vila (stem@industry.gov.au)

*BMC Proceedings 2023*, **17(15):**A14

Science, technology, engineering and mathematics (STEM) skills are the foundation of a thriving economy. However, Australian women and girls remain critically underrepresented in STEM education and careers. The causes are broad, complex and long-standing, and participation data is housed in a number of disparate sources, collected using differing methodologies. These limitations impact the ability of public and private sectors to effectively inform evidence-based policies that aim to improve gender equity in STEM.

The Australian Government’s STEM Equity Monitor (the Monitor) is a 10-year commitment to leading a data-driven approach to informing gender equity in STEM [1]. It is aligned to the government’s Advancing Women in STEM Strategy and 2020 Action Plan.

The Monitor consolidates a range of relevant data, spanning early-education to senior career positions, to showcase Australian STEM participation rates by gender. The Monitor also supplements existing data (including some previously unpublished) with commissioned reports to address key data gaps. For example, YouthInsight’s ‘Youth in STEM’ and ‘STEM influencers’ reports highlight perceptions and attitudes toward STEM of Australia’s youth, parents and educators [2].

The Monitor shows that, despite encouraging recent signs of progress toward STEM gender equity, there is still a long way to go. Two improved areas include: the increased proportion of women across all STEM-qualified industries to 28% in 2020 (from 24% in 2016) and the increased proportion of women in management positions (excluding chief executives) to 23% in 2020 (from 18% in 2016).

The Monitor is a useful tool for researchers and policymakers as data consolidation increases the visibility of existing data. It provides a comprehensive analysis of women’s and girls’ participation across various STEM pathways. The Monitor also provides trend analysis through annual data updates, which tracks progress and identifies areas that require greater focus to achieve gender equity.

The Monitor is published in two different formats, which is crucial for reaching various audiences. It is available as an interactive online report (utilising Power BI), which allows users to explore the data in detail. It is also published as a static highlights report, providing a high-level summary of key trends.

The Monitor has been used to identify gaps for further government support and interventions. For example, the design of round 3 of the Women in STEM and Entrepreneurship grants was informed by the Monitor’s data. It showed areas with low levels of engagement by girls and women, and informed the round’s four key strategic areas: information technology, engineering, entrepreneurship and intersectionality.

In summary, the Monitor is an important tool that can be used by both private and public actors to inform evidence-based practises and policies in the gender equity space.

The Monitor was first published in 2020 and is updated annually. The fourth edition (published in 2023) is currently available online [1].

**References**

1. Department of Industry, Science, Energy and Resources (DISER). STEM Equity Monitor [Internet]. Canberra: DISER; 2022 [updated 2023 July 20; cited 2022 Mar 7]. Available from: https://www.industry.gov.au/publications/stem-equity-monitor.
2. Department of Industry, Science, Energy and Resources (DISER). Youth in STEM research project. Canberra: DISER; 2019 [cited 2022 Mar 7]. Available from: https://www.industry.gov.au/data-and-publications/youth-in-stem-research-project.

## A15 Funding innovation: Western Sydney University’s Vice-Chancellor’s Gender Equity Fund

### Kieryn McKay

#### Western Sydney University, Parramatta, Australia

##### **Correspondence:** Kieryn McKay (k.mckay@westernsydney.edu.au)

*BMC Proceedings 2023*, **17(15):**A15

Recognised as an outstanding initiative within the SAGE Athena SWAN Bronze Institutional Award process, Western Sydney University’s Vice-Chancellor’s Gender Equity Fund (VC GE Fund) is the first of its kind in the Australian higher education sector. Established and sponsored by the University’s Vice-Chancellor in 2016 and overseen by the Vice-Chancellor’s Gender Equity Committee, the VC GE Fund is a funding innovation that funds innovation: an internal grant that furnishes academic and professional staff with resourcing to undertake gender equity and diversity research or initiatives that have the potential to make tangible impact at the University.

Now in its sixth year of operation, the VC GE Fund has become an essential mechanism for generating new knowledge and innovative practice for gender equity, diversity and inclusion at Western Sydney University. To date, more than $200,000 has been granted to projects that have strengthened and expanded the University’s understanding of its gender equity challenges and opportunities, helping to focus the University’s gender equity efforts for the benefit of staff, students and community. As a direct result of VC GE Fund projects evidence-informed improvements have been made to University breastfeeding facilities, Academic Promotions processes, targeted supports for parents and carers, and staff and student training on gender equity and diversity matters (among others). New recommendations are forthcoming for enhancing the University’s intersectional approach to gender equity and targeting its responsiveness to the gendered impacts of COVID-19.

Across the life of this initiative, much has also been learned about how to optimise the operations of the VC GE Fund itself. The University has progressively consulted and clarified priorities for new research and programs, refined Fund guidelines to safeguard project success, improved oversights for monitoring progress, introduced new mechanisms to ensure the delivery of practical and implementable recommendations, and increased both internal and sector-facing engagement with our project findings and outcomes. Each of these improvements has shaped a more sustainable and more impactful initiative with greater strategic effectiveness and increasing reach both within and beyond our institution.

This presentation shares the best-practice insights we have accrued across six years of implementing the Vice-Chancellor’s Gender Equity Fund at Western Sydney University. As more higher education institutions look to adopt this funding model (or similar) into their own contexts, we offer learnings about the benefits, but also the hurdles and challenges we have encountered along the way. In doing so, we map the administrative and strategic refinements made to realise new potentials for this initiative at our organisation, with the aim of guiding others to effectively implement such funding innovations of their own.

## A16 "Everybody needs a little MAGIC": mid-career scientists reflect on the impact of Mentoring and Guidance in Careers (MAGIC) Workshops

### Joanna Sikora, Merryn McKinnon

#### Australian National University, Canberra, Australia

##### **Correspondence:** Joanna Sikora (joanna.sikora@anu.edu.au)

*BMC Proceedings 2023*, **17(15):**A16

**Background**

Mentoring and Guidance in Careers Workshops (MAGIC) were five-day long residential training and networking events held at the Australian National University (ANU), initiated by Professors Mahananda Dasgupta (physics – ANU) and Nalini Joshi (mathematics – University of Sydney) as part of their ARC Georgina Sweet Fellowships. Open to female-identifying scientists within the first seven years of their career in the typically male-dominated fields of mathematics, physics, chemistry and related fields, the fully-funded workshops attracted about 35 participants each year between 2017 and 2019. MAGIC aimed to provide participants with resources to enable long-term career planning and overcome cultural or institutional challenges to forge a successful career. Experts from academia, government and industry contributed to panel discussions and exercises and provided participants with individual assistance. Most importantly, participants shared their successes and the challenges they faced at the early stages of their careers with peers and senior leaders in cognate disciplines. Feedback immediately following each MAGIC workshop showed early-career scientists found MAGIC extraordinarily relevant and helpful – but did that withstand the test of time?

**Methods**

Towards the end of 2021, we interviewed 49 out of 106 participants, capturing comprehensive reflections on the perceived impact that MAGIC workshops had on participants' lives in the two to four years that followed. In total, the semi-structured interviews took over 50 hours and generated nearly half a million words in transcripts.

**Results**

In this talk, we present the results of a preliminary thematic analysis, focusing on the perceived benefits of such a workshop format.

**Conclusions**

We highlight what aspects of MAGIC work to support early-career scientists in male-dominated fields and what areas need more focus to create more inclusive and equitable systems. Aside from offering insight into effective mechanisms and inclusions for use in gender equity initiatives, we will also reflect on the challenges of assessing the effectiveness of MAGIC and other gender equity interventions [1] and highlight the benefits of adopting a longitudinal approach such as ours.

**Reference**

1. McKinnon M. The absence of evidence of the effectiveness of Australian gender equity in STEM initiatives. Aust J Soc Issues. 2022;57:202-14.

## A17 The power of conversation – lessons from incorporating social justice issues into human physiology and employability curriculum

### Fiona J Baird

#### Griffith University, Gold Coast, Australia

##### **Correspondence:** Fiona J Baird (f.baird@griffith.edu.au)

*BMC Proceedings 2023*, **17(15):**A17

As a physiology lecturer in the higher education sector, I was never expected to explore social justice issues such as racism, gender stereotypes or discrimination with the Health students who take my courses. After undertaking active bystander training in the domestic violence landscape, I recognised the opportunity to share these skills with my undergraduate students as they are transferable between their professional and personal lives. In collaboration with the MATE Bystander Program at Griffith University, a series of ten 15-minute activities called MATE Bytes were developed and embedded in a first trimester, first-year human physiology course covering social justice issues in 2020. This cohort consisted of over 500 students from over 30 degrees across the Health disciplines. An associated microcredential was also provided for students who wanted to formalise their active bystander training. In the same year, a third-year employability course had the same social issues embedded in its curriculum with principles of inclusion and equality governing the interactions in the workshops.

The general framework is to set up a safe and brave space [1] and link the issue with the curriculum. For example, in the physiology course, we discussed the issue of racism in the same week where we went through the structure and function of skin. This provided a direct link to the physiology and how some people wrongly discriminate based on a pigment deposited in the skin. Due to the online learning environment, we allowed comments to be anonymous; however, several students chose to identify themselves to share their lived experience. Based on student feedback we extended the time from 15 to 30 minutes in the second year of the program. For the employability course, one example we examined in-person was the perception of professional competency is affected by gender and diversity bias.

Over the last two years, we have had incredible interactions through these two courses that demonstrate that just discussing these issues has changed behaviours immediately and plants the seed for further exploration. By using safe and brave spaces, we have had students share their lived experiences that have opened minds and hearts across our diverse student population. In 2020, 39 MATE Bytes microcredentials were awarded and this increased to 51 in the 2021 cohort. The extended impact of these conversations has been far reaching. Years later, students have reported back using their skills. Some have changed jobs after realising the behaviours they were subject to were harassment or discrimination. Others have initiated conversations ranging from questioning artwork hanging in libraries that objectify and exploit subjects, to identifying biases in our language that exclude the LGBTQI+ community when talking about domestic violence. However, the most common outcome reported back is a feeling of empowerment and validation that any bystander action they take leads to a more inclusive community for all.

**Reference**

1. Arao B, Clemens K. From safe spaces to brave spaces. In: Landerman LM, editor. The art of effective facilitation: reflections from social justice educators. Stirling, Virginia: Stylus Publishing, LLC; 2013. p. 135-50.

## A18 Advancing women in healthcare leadership: a national organisation level, research and translation partnership

### Helena Teede^1,2*^, Jenny Proimos^1^, Mariam Mousa^1^, Madison Hartill-Law^1^, Belinda Garth^1^, Jacqueline Boyle^1^, on behalf of the AWHL Investigator Study Group

#### ^1^Monash Centre for Health Research and Implementation, Monash University, Clayton, Victoria,Australia; ^2^Monash Health, Clayton, Victoria, Australia

##### **Correspondence:** Helena Teede (helena.teede@monash.edu)

*BMC Proceedings 2023*, **17(15):**A18

**Background**

Healthcare is a major Australian employer and has a 75% female workforce [1]. Yet, here and internationally, women are under-represented in healthcare leadership, failing to reflect our community or workforce [1–3]. Major barriers to change persist [2–4]. Healthcare lacks sector-wide, evidence-based, organisational approaches to drive effective change, leaving the burden to individuals to battle change alone [4]. Current efforts are ad hoc, duplicative and of limited effectiveness and reach. Partnership, research and translation are urgently needed to deliver multi-faceted organisational change for measurable improvement in equity [4].

We aim to create a national partnership across broad stakeholders and cross-sector academic expertise to co-design and deliver effective organisational change to Advance Women in Healthcare Leadership (AWHL).

**Methods**

Stakeholder partnership, a shared vision, resourcing, evidence synthesis (systematic review, meta-synthesis and meta-ethnography), workshops, priority setting, qualitative research, co-design and implementation research with intervention, and evaluation, capturing and sharing learning for broad scale-up and impact.

**Results**

Partnership across Professional Medical and Nursing Colleges, leading health services, Government and academia (led by Monash University) has been established. A shared vision to advance women in healthcare leadership has been developed. Resourcing was obtained via partner contributions and a National Health and Medical Research Council (NHMRC) Partnership Grant. Evidence synthesis identified evidence-based interventions and implementation strategies [5]. Co-design processes generated four research themes: Organisational Change Management; Leadership Development Programs; Nursing Leadership; and Role of Member Organisations. Implementation research is currently underway with partners to tailor and support these key organisations to co-design and operationalise their programs of evidence-based work.

**Conclusions**

Recognition of the importance of gender inequity in healthcare leadership in Australia is high. Engagement and partnership have been established nationally. Evidence-based approaches and priority areas for collective action have been identified. Project partners are developing gender equity and diversity plans and work is underway to inform and support each partner in their work and to enable collective action to implement evidence-based organisational change to advance women in healthcare leadership. Partners and those interested to engage are welcome [https://www.womeninhealthleadership.org/; mchri.awhl@monash.edu].

**References**

1. McDonagh K, Bobrowski P, Hoss M, Paris N, Schulte M. The leadership gap: ensuring effective healthcare leadership requires inclusion of women at the top. Open Journal of Leadership. 2014;3:20-9.
2. Bismark M, Morris J, Thomas L, Loh E, Phelps G, Dickinson H. Reasons and remedies for under-representation of women in medical leadership roles: a qualitative study from Australia. BMJ Open. 2015;5(11):e009384.
3. Weiss A, Lee KC, Tapia V, Chang D, Freischlag J, Blair SL, Ramamoorthy S. Equity in surgical leadership for women: more work to do. Am J Surg. 2014;208(3):494-8.
4. Teede HJ. Advancing women in medical leadership. Med J Aust. 2019;211(9):392-394.e1.
5. Mousa M, Boyle J, Skouteris H, Mullins AK, Currie G, Riach K, Teede HJ. Advancing women in healthcare leadership: a systematic review and meta-synthesis of multi-sector evidence on organisational interventions. EClinicalMedicine. 2021;39:101084.

## A19 Beyond binary: a practitioner’s account of implementing gender identity and expression initiatives at Royal College of Surgeons in Ireland

### Julia R Morrow

#### RCSI, Dublin, Ireland

##### **Correspondence:** Julia R Morrow (juliamorrow@rcsi.com)

*BMC Proceedings 2023*, **17(15):**A19

**Background**

In Ireland, Transgender Equality Network Ireland’s (TENI) 2013 report, *Speaking from the Margins*, indicated 1–2% of people in Ireland identify as trans [1]. This report also highlighted trans people’s experiences of poor mental health; 80% of respondents had contemplated suicide and half had made at least one attempt (n = 113) (ibid). In January 2022, Dr Karl Neff, endocrinologist and clinical lead at the National Gender Service (NGS) at St Columcille’s Hospital in Dublin reported an increasing demand for the NGS, from referral rates of 10 people per year in the early 2000s when the NGS was initially established to more than 300 referrals in 2021.

This project outlines initiatives driven by the Equality, Diversity and Inclusion (EDI) Unit to ensure RCSI’s organisational culture and environment is inclusive and equitable for transgender people [2]. This work to advance gender equality has also been recognised as beacon activity in RCSI’s institutional Athena SWAN Bronze award. RCSI University of Medicine and Health Sciences is an academic and research institution as well as the national surgical training body for Ireland.

**Methods**

Over the past 5 years, RCSI has implemented several interventions aimed at making the University a more inclusive place for non-binary people. These interventions include: 1) providing gender neutral, or universal access, toilets and shower facilities; 2) facilitating “Trans 101” training delivered by the Transgender Equality Network Ireland (TENI) for staff and students; 3) delivery and launch of a Gender Identity and Expression Policy (the third university in Ireland to launch and implement such a policy); 4) establishing a LGBTI+ staff network (prior to the EDI Unit’s establishment, there is a thriving student PRIDE society); 5) hosting annual Pride celebrations; 6) raising the Pride and trans flags above RCSI annually since the inaugural raising in 2018; 7) supporting staff members transition at work (formally, e.g., updating HR, IT and pension records, or informally); 8) introducing the option of adding your pronouns to your email signature; 9) expanding the question set in RCSI's annual EDI Staff Survey to include a question capturing trans history to inform future actions and policies. The objective of introducing these initiatives is to foster a trans inclusive environment, increase engagement in LBGTI+ related activities and strengthen the values of dignity and respect for all at RCSI.

**Results**

The analysis of the survey results, event attendances and staff network membership count is underway.

**Conclusions**

The preliminary findings indicate the interventions are achieving the desired outcomes. However, the work is not complete, but ongoing, as RCSI continues to drive equality and positive impact for staff and students.

**References**

1. McNeil J, Bailey L, Ellis S, Regan M. Speaking from the margins: trans mental health and wellbeing in Ireland [Internet]. Transgender Equality Network Ireland (TENI); 2013. Available from: https://bit.ly/2P8jIbI.
2. Beemyn BG. Making campuses more inclusive of transgender students. Journal of Gay & Lesbian Issues in Education. 2005;3(1):77-87.

## A20 The role of professional member organisations in promoting gender equity and advancing women in healthcare leadership

### Jenny Proimos^1,2*^, Jacqueline Boyle^1,2^, Madison Hartill-Law^1,2^, Helena Teede^1,2^

#### ^1^Monash Centre for Health Research and Implementation, Clayton, Australia; ^2^Monash University, Clayton, Australia

##### **Correspondence:** Jenny Proimos (jenny.proimos@monash.edu)

*BMC Proceedings 2023*, **17(15):**A20

**Background**

There has been significant attention on gender inequities within medical and surgical specialties recently. Research has highlighted the barriers to entry and career advancement for women which have resulted in an inequity in gender distribution of medical and surgical specialists [1].

Professional member organisations, such as Medical Colleges, play a crucial role in medical training and careers; development of standards and curricula for postgraduate vocational training; control of entry into and exit out of vocational training programs; development of accreditation standards and accreditation of health services for vocational training; continuing professional development; leadership opportunities through participation in governance committees. This project aimed to identify evidence-informed interventions promoting gender equity already being delivered by professional member organisations, and to assess the appetite for collective co-design and implementation of potential relevant interventions.

**Methods**

A scoping review of literature and publicly available data was undertaken, including analysis of member organisation stakeholders’ current policies, procedures, standards, guidelines and regulations relating to vocational training, discrimination, bullying, inclusion and accreditation. Current activities were classified according to evidence-informed interventions known to promote gender equity [2]. A workshop with member organisation stakeholders was held to explore issues relevant to each organisation, to communicate findings from the scoping review and to determine appetite and priorities for potential collective action.

**Results**

Most member organisations involved had already established gender equity or diversity and inclusion working groups. Two organisations had published gender equity reports, with agreed targets for representation on boards, councils and committees. One member organisation had reported progress on their targets. While most organisations had published policies on flexible training, parental leave and interrupted training, implementation was not reported. Accreditation standards allowed for fractional training, subject to staffing levels and hospital requirements. Transparent data on selection into training programs and member organisation committees was variably available. Specific leadership training and development programs were not found. Most member organisations did not provide formal career mentoring or networking. Member organisation stakeholders agreed that collective action would likely magnify their impact on healthcare services. Potential interventions discussed included ownership by their leadership of gender equity focused culture change, enforcement of accreditation standards which promote flexible training, enterprise bargaining agreement negotiations and development of collective position statements for advocacy. Platforms such as the College of Presidents of Medical Colleges and the Australian Medical Council were suggested to drive collective action more systematically.

**Conclusions**

Professional member organisations recognise their role in promoting gender equity, and have begun to establish targets, policies and interventions, but are still early in their journey of implementation. They recognise the opportunity to amplify the impact of interventions by working collectively.

**References**

1. Burgess S, Shaw E, Ellenberger KA, Segan L, Castles AV, Biswas S, Thomas L, Zaman S. Gender equity within medical specialties of Australia and New Zealand: cardiology's outlier status. Intern Med J. 2020;50(4):412-9.
2. Mousa M, Boyle J, Skouteris H, Mullins AK, Currie G, Riach K, Teede HJ. Advancing women in healthcare leadership: a systematic review and meta-synthesis of multi-sector evidence on organisational interventions. EClinicalMedicine. 2021;39:101084.

## A21 What do Athena SWAN applications show us about addressing workplace sexual harassment in the Australian university sector?

### Alicia C Pearce

#### University of Technology Sydney, Sydney, Australia

##### **Correspondence:** Alicia C Pearce (alicia.c.pearce@student.uts.edu.au)

*BMC Proceedings 2023*, **17(15):**A21

Examining the rich evidence base provided by the Australian Athena SWAN applications, I consider the ways that Universities administer workplace sexual harassment prevention and remedy in academic and professional staff workplaces. I present new insights into its administration across the Australian higher education sector, its effectiveness and evidence of interventions brought about through the SAGE Athena SWAN pilot.

My research extracts data from the full cohort of Athena SWAN applications and action plans on what Universities have said about their existing and proposed policies, practices and prevalence of workplace sexual harassment. This data is coded and analysed against a research-informed framework developed by Mcdonald et al. [1], that describes effective organisational interventions to prevent and address sexual harassment in the workplace. I consider how developed primary, secondary and tertiary strategies are across the sector, and what these strategies are.

This research is part of my broader doctoral research project creating a whole of sector view of the University employer strategies to address workplace sexual harassment, how they are formulated and what kind of regulation would assist the sector to create progressive change. Developed policies are supported by cultures that assess risk factors and reward managers who respond appropriately to harassment claims [1]. My research contributes to a growing body of literature on Athena SWAN interventions and outcomes, mostly conducted in UK universities [2–5].

**Acknowledgements**

I acknowledge the support of the UTS Quentin Bryce Scholarship in conducting this research.

**References**

McDonald P, Charlesworth S, Graham T. Developing a framework of effective prevention and response strategies in workplace sexual harassment. Asia Pacific Journal of Human Resources. 2015;53(1):41-58.
Gregory-Smith I. Positive action towards gender equality: evidence from the Athena SWAN Charter in UK medical schools. Br J Ind Relat. 2018;56(3):463-83.
3.Rosser SV, Barnard S, Carnes M, Munir F. Athena SWAN and ADVANCE: effectiveness and lessons learned. Lancet. 2019;393(10171):604-8.
Kalpazidou Schmidt E, Ovseiko PV, Henderson LR, Kiparoglou V. Understanding the Athena SWAN award scheme for gender equality as a complex social intervention in a complex system: analysis of Silver award action plans in a comparative European perspective. Health Res Policy Syst. 2020;18:19.
Ovseiko PV, Chapple A, Edmunds LD, Ziebland S. Advancing gender equality through the Athena SWAN Charter for Women in Science: an exploratory study of women’s and men’s perceptions. Health Res Policy Syst. 2017;15:12.

## A22 "STEM x"- university/school collaboration through design thinking: a solution for gender in STEM outreach interventions

### Marco Angelini

#### UTS, Sydney, Australia

##### **Correspondence:** Marco Angelini (marco.angelini@uts.edu.au)

*BMC Proceedings 2023*, **17(15):**A22

**Background**

Across Australia, women are under-represented in engineering and IT at all levels; these gaps have not shifted over the past two decades [1,2]. The 2019 Women in STEM Decadal Plan published by the Australian Academy of Science identified barriers to participation as occurring from early school years, with teachers, families and socio-cultural factors as key influencers. The STEM X program vision, devised by Women in Engineering and IT at UTS as a response to the 2019 Decadal Plan, is that STEM study and career journeys should not be limited by gender. Our research-based analysis shows that intervening early has the greatest long-term impact in gender equity, particularly among schools in less advantaged areas. This presentation will outline how the project’s aim to increase girls’ confidence, interest and awareness in STEM studies in the short term, and participation in STEM careers in the long-term, is showing early promise in outcomes.

**Methods**

Our STEM X outreach model was revised in 2019 to address early barriers to participation. The focus shifted to multiple interactions in a 4-8 week in-curriculum, in-classroom program involving university students and industry mentors as facilitators; as a result our primary and high school programs centre around gender-inclusive STEM project learning involving students, teachers and families as key stakeholders. We use design thinking processes that involve educational technologies to ideate solutions to real world challenges self-identified by students who then build their project solution prototypes. Our focus on female students, their school and wider community that surrounds them, building of long-term teacher capacity and intervening early from primary school onwards is a unique model that is a sector leader in Australia.

**Results**

This presentation will use three years of data from 2019 to map the early impacts of our program delivery. Results will be discussed from matched participant pre and post activity surveys, showing which quantitative areas show statistically significant changes. There will also be a discussion of the qualitative data we gathered to outline the changes in self perceptions among female participants.

**Conclusions**

Although our aim is longitudinal impact, our evaluation embedded into the program is already starting to see significant increased interest and confidence among female students. This presentation will outline the key features of the STEM X program, underlining its innovative features and mapping out future developments. There will be a discussion on how to address teacher confidence through professional development including removing unconscious bias in the classroom and encouraging girls to opt-in to further STEM opportunities, as well as engaging parents through improved communication strategies.

**References**

1. Holmes K, Gore J, Smith M, Lloyd A. An integrated analysis of school students’ aspirations for STEM careers: which student and school factors are most predictive? Int J Sci Math Educ. 2018;16(4):655-75.
2. Olsson M, Martiny SE. Does exposure to counterstereotypical role models influence girls' and women's gender stereotypes and career choices? A review of social psychological research. Front Psychol. 2018;9:2264.

## A23 A diversity and inclusion toolkit by the Australian Academy of Technology and Engineering

### Candice Raeburn, Nicola Smillie, Lachlan Blackhall, Leeanne Bond, Gunilla Burrowes, Adi Paterson

#### Australian Academy of Technology and Engineering, Canberra, Australia

##### **Correspondence:** Lachlan Blackhall (lachlanblackhall@gmail.com)

*BMC Proceedings 2023*, **17(15):**A23

The Australian Academy of Technology and Engineering (ATSE) has developed a toolkit to assist small and medium enterprises (SMEs) in fostering a more diverse and inclusive workforce.

The Diversity and Inclusion (D&I) Toolkit was developed in response to the findings of the Women in STEM Decadal Plan, co-written by ATSE and the Australian Academy of Science (AAS) in 2019. The Decadal Plan found that the tools available to science and technology SMEs to address issues of gender inequality were insufficient.

In response to these findings, ATSE held a workshop with SME leaders to discuss the challenges these business face in relation to gender equity. The outcome of this workshop was the production of the D&I Toolkit, focusing not just on gender equity, but diversity and inclusion more broadly.

The D&I Toolkit explores the moral, legal and economic imperatives behind diverse hiring, and emphasises how workplace diversity boosts business performance. The Toolkit then delves into a reference guide on how best to Recruit, Retain, and Reach a diverse workforce.

Recruit is designed to assist SME leaders in understanding and addressing the barriers preventing diverse talent from applying. This section also includes suggestions and resources to ensure recruitment processes are inclusive, accessible, and mitigate bias.

Retain discusses the different approaches to making the workplace more inclusive, accessible, and respectful, to ensure SMEs can retain a diverse workplace.

Reach helps SMEs ensure promotional activities are inclusive, and guides leaders to adopt best-practice promotional and professional development opportunities to all employees.

The D&I Toolkit is currently a digital only tool, however there is the possibility in the future it could be developed into an interactive website. The D&I Toolkit is now being piloted, with 10 businesses unique in size, function and capacity working through the toolkit at their own pace until the pilot’s conclusion in June 2022.

Throughout this pilot stage, each of these businesses are being offered the opportunity to provide monthly feedback, as well as the opportunity to schedule a meeting with the ATSE team. This allows the SMEs to share their progress to date or ask any questions that may have arisen as they have worked their way through the Toolkit.

At the conclusion of the pilot stage, each business will be asked to complete a final evaluation of the project. This evaluation will be completed in line with the Australian Government’s guide for evaluating STEM gender equity programs. This feedback will then be incorporated into the final version of the D&I Toolkit [https://www.atse.org.au/research-and-policy/publications/publication/diversity-and-inclusion-toolkit/].

## A24 “This is not necessarily a gender thing”: gender-blind talk about flexible work

### Marianne H Clausen^1*^, Amanda LeCouteur^1^, Shona Crabb^1^, Anna Chur-Hansen^1^, Niki Vincent^2^

#### ^1^The University of Adelaide, Adelaide, Australia; ^2^Commission for Gender Equality in the Public Sector, Melbourne, Australia

##### **Correspondence:** Marianne H Clausen (marianne.clausen@adelaide.edu.au)

*BMC Proceedings 2023*, **17(15):**A24

**Background**

Patterns of paid employment and domestic/care work in Australia are typically divided according to gender. Women often combine paid employment with domestic/care tasks, while men engage in paid employment and tend not to use flexible work arrangements. While flexible work has helped facilitate women’s presence in paid employment, workplace gender equality relies on men using flexible work, to prevent flexible work from being devalued and feminised [1]. Workplace policies are increasingly presented in gender-neutral terms, theoretically providing access to all employees. There is also increasing evidence suggesting that men would like to use flexible work arrangements and spend more time with their families [2]. Despite the removal of barriers to men’s flexible work, and men reporting a desire to use flexible work, there are continued gender differences and underuse of flexible work by men.

**Methods**

Organisational leaders are a crucial element in the provision and use of flexible work, both in terms of setting policy and workplace culture [3], and are, therefore, the focus of this study. Interviews were conducted with twelve leaders of various public sector departments and private companies, including large multi-national companies. Qualitative analysis was undertaken, using thematic discourse analysis and Stoll’s [4] theory of gender-blind sexism.

**Results**

Participants frequently avoided talking about gender when being interviewed, despite being aware they were participating in research about men’s flexible work. This seeming reluctance led the researchers to question how gender was attended to and managed in discussions of flexible work. It was found that participants attended to gender in particular ways, often conforming to the style and frames of gender-blind sexism. Two broad themes were developed from the data, with participants (1) avoiding talking about men, or (2) discussing men and women with regard to traditional gender roles. Both themes are supported by the theory of gender-blind sexism. The data also supported a suggestion for an expanded concept of gender-blind sexism that incorporates a novel finding of emphasising gender.

**Conclusions**

Gender-blind sexism contributes to current understandings of subtle sexism and explanations of gender inequality, particularly in situations claiming to be gender-neutral, such as modern organisations. Leaders’ discussions of men’s flexible work have the potential to perpetuate workplace cultures where men are not visible as flexible workers and where gendered patterns of work and care are reinforced.

**References**

1. Workplace Gender Equality Agency [Internet]. Sydney: Workplace Gender Equality Agency. Workplace flexibility. 2018. Available from: https://www.wgea.gov.au/topics/workplace-flexibility
2. McCurdy S. “You did what?": taking the daddy track. In: Breekveldt N, editor. Career interrupted: how 14 successful women navigate career breaks. Melbourne: Melbourne Books; 2015. p. 178-89.
3. Borgkvist A, Moore V, Crabb S, Eliott J. Critical considerations of workplace flexibility “for all” and gendered outcomes: men being flexible about their flexibility. Gend Work Organ. 2021;28(6):2076-90.
4. Stoll LC. Race and gender in the classroom: teachers, privilege, and enduring social inequalities. Plymouth: Lexington Books; 2013.

## A25 Moving towards fair and equitable workload allocation: piloting the Workload Allocation Principles Matrix

### Elizabeth N Mackinlay^1*^, Cate Thomas^2^, Tara Magdalinski^3^

#### ^1^Southern Cross University, Gold Coast, Australia; ^2^Charles Sturt University, Wagga Wagga, Australia; ^3^Swinburne University, Melbourne, Australia

##### **Correspondence:** Elizabeth N Mackinlay (mermaidliz21@gmail.com)

*BMC Proceedings 2023*, **17(15):**A25

There is a vast and growing body of evidence which tells us that in response to a performance-based demand for increased efficiency and accountability in universities, academic and administrative workloads have increased dramatically over the past ten years—indeed, this was identified as a common theme across many applications for the SAGE Athena Swan Bronze Awards. The onset of COVID-19 has simultaneously further transformed, disrupted, and exacerbated academic and administrative labour, so that the impact of such workload intensification and change disproportionately affects particular groups of university employees, notably women. The SAGE Special Interest Group (SIG) on Workload recognises then that there is an urgent need for fair and equitable models for managing workload allocation across the sector which challenge and address such inequities, contribute to post–COVID-19 recovery, and empower universities to drive change in this space.

Adopting a collaborative approach, the Workload SIG has been meeting fortnightly since 7 April 2021 to share insight on practices, problems and possibilities in the development of a fit-for-purpose guide of fair and equitable workload allocation for staff at all levels—the Workload Allocation Principles Matrix (or the Matrix). The SIG comprises members from across regions, with representatives from the ACT, NSW, QLD, WA and VIC and includes professional staff, academic staff and university executives, giving a broad perspective and wealth of knowledge on workload allocation issues.

We know that every university grapples with how to most efficiently allocate workload across its staff, with different institutions using single or multiple workload models, centralised or localised systems, sophisticated planning tools or simple spreadsheets, all with varying levels of engagement and accountability. The tool does not dictate how workload should be allocated but rather invites open reflection within the institution. In this workshop, we will introduce the Matrix, describe and deconstruct each principle, provide attendees with a guide to how the matrix might be used, ask representatives to consider how the Matrix might be used and applied in their respective institutions, and invite reflection on the effectiveness of the Matrix tool.

The feedback from this workshop will contribute significantly to refinement of the Matrix and the ways in which it is taken up my institutions in workload allocation conversations, particularly in the current climate of negotiating new enterprise bargaining agreements.

## A26 A review of strategies that advance female nursing careers

### Mihirika Surangi De Silva Pincha Baduge^1,2*^, Mariam Mousa^1^, Leanne Boyd^3^, Helena Teede^1,4^

#### ^1^Monash University, Clayton, Australia; ^2^Austin Health, Heidelberg, Australia; ^3^Eastern Health, Boxhill, Australia; ^4^Monash Health, Clayton, Australia

##### **Correspondence:** Mihirika Surangi De Silva Pincha Baduge (mihirika.pinchabaduge@monash.edu)

*BMC Proceedings 2023*, **17(15):**A26

**Background**

Nurses bring unique perspectives to board rooms in areas related to strategic planning, critical thinking, communication, quality and process improvement, human resources, finance, and complex problem-solving. However, the nursing profession is gendered, which often undervalues its contribution leading to a lack of recognition. Nursing, often perceived as a caring role, has been typically stereotyped as feminine work with fewer men choose it as a career. While there are far fewer men in nursing, the ‘glass escalator’ finds male nurses climbing the leadership ladder faster than their female counterparts. Strategies for nurses to attain leadership positions are studied and reported on, without a specific gender lens. This systematic review explores the evidence for organisational-level strategies that advance female nurses in their careers.

**Methods**

Four large databases were searched using search terms: leadership, OR career mobility, OR career progression, OR career advancement, AND academia, OR health services, AND female, OR women. For this additional analysis, the term nurs* was also included. A total of six studies met the inclusion criteria and were included in this analysis. Data were extracted narratively to identify the barriers, facilitators and organisational-level interventions that support the advancement of female nurses in leadership based on their career stage and setting including academia, clinical or global health.

**Results**

Organisational interventions such as leadership training, mentorship and networking, and financial support have been found to be effective across nursing career stages (early-, mid-, and senior-). Career assistance, orientation programmes, shared experiences, and stories of successful senior nurse academics were reported as supportive organisational level interventions for early-career nurse academics. Executive training programmes were found to be an effective leadership development intervention for senior clinicians.

**Conclusions**

Mentorship, networking, financial support, leadership trainings and leadership opportunities are highly cited organisational level interventions for advancing nurses in healthcare leadership.

## A27 Diversity Moments: taking 4 minutes to embed diversity concepts in a science organisation

### Patrick Laffy, Sharon Barnwell

#### Australian Institute of Marine Science, Townsville, Australia

##### **Correspondence:** Sharon Barnwell (sharon.barnwell@gmail.com)

*BMC Proceedings 2023*, **17(15):**A27

Diversity and equity initiatives are an important part of structuring a fair and equitable science research environment, and the benefits of creating this environment are numerous and well-documented. A major hurdle faced by research organisations attempting to implement diversity initiatives is finding the space and time to communicate the benefits of diversity and equity interventions. The Australian Institute of Marine Science (AIMS) has introduced the “Diversity Moment” initiative to deliberately create this space and time. Building on the organisation’s existing communication strategy for workplace safety initiatives, the Diversity Moment is a 4-minute speaking slot included in fortnightly All-Staff Meetings to highlight aspects of diversity.

First presented by members of AIMS’ internal equity and diversity working group, a nominated group member communicated a brief story or reflection on a diversity, equity or inclusion topic at a scheduled webinar. This segment provided staff, students and other invited guests opportunities for reflection and consideration on matters beyond the business of scientific research. In parallel, it has established the importance of gender equity, diversity and inclusion matters as being on equal footing with safety in workplace culture.

Staff feedback and internal review identified that sharing the presentation of Diversity Moments with any staff member or student at AIMS had the potential to increase the impact of this initiative. By widening participation, the Diversity Moment has created awareness of a broader range of issues and given more voices the opportunity to offer personal reflections on topics that matter to them with colleagues across all sites.

The Diversity Moment is helping to create an environment where staff and students can freely discuss broader diversity issues, as well as to communicate and educate co-workers on real-world experiences that impact their daily life. This presentation will share some of the Diversity Moments presented by staff, examples of the feedback received from listeners, and make the case that four minutes per fortnight is an effective tool to embed diversity, inclusion and gender equity matters in a science organisation’s mindset.

## A28 Achievement relative to opportunity – beyond academic promotion

### Mat Lewis^1^, Madhu Bhaskaran^2^, Maddy Yewers^3^, Amy Love^1^, Kay Latham^3*^, Carol Corzo^1^

#### ^1^People Team, RMIT University, Melbourne, Australia; ^2^ARC Centre of Excellence for Transformative Meta-Optical Systems (TMOS), STEM College, RMIT University, Melbourne, Australia; ^3^SAGE Athena SWAN Project Team, STEM College, RMIT University, Melbourne, Australia

##### **Correspondence:** Kay Latham (kay.latham@rmit.edu.au)

*BMC Proceedings 2023*, **17(15):**A28

Our commitment to equity and gender equity is based on a concrete program of strategy and action under Athena SWAN. We focus on removing entry barriers using substantive equity practices with diversity and intersectionality as key principles.

Achievement Relative to Opportunity (ARtO) is an equity enabler, essential to the equitable positioning of women and diverse talent in academia. Most higher education institutions apply ARtO to academic promotion, yet few organisations have embedded ARtO across the employee lifecycle. The sector is still grappling over how to apply ARtO in recruitment, performance review and other talent management practices. RMIT investigated the application of ARtO beyond academic promotion to other areas of the employee lifecycle, specifically recruitment.

Evidence from ARtO in academic promotion shows that it can improve career outcomes and retention for staff by changing the paradigm in decision-making away from the traditional concept of ‘merit’. Changing traditional methods of evaluating merit in the employee lifecycle has the potential to allow women and people from diverse backgrounds to be fairly evaluated and shift the dominance of people who have experienced little or no career interruption.

The study design included: a consultation process; review of academic promotion implementation and practices; embedding ARtO in recruitment (guidelines for hiring managers, information for candidates on external careers site, training for recruitment teams, job advertisement language and application process design).

The implementation process was piloted with the newly established ARC Centre of Excellence for Transformative Meta-Optical Systems (TMOS), in conjunction with the use of Special Measures for women, trans and gender diverse, and Aboriginal and Torres Strait Islander applicants. The learnings from the pilot were subsequently applied to a large excellence in research campaign, in conjunction with the use of Special Measures. This is RMIT’s most complex recruitment process.

Within TMOS, ARtO and Special Measures recruitment resulted in high engagement with the process. Many applicants activated ARtO in their applications, including a third of the shortlist. ARtO gave the hiring managers a key understanding of diverse career journeys and impacts on track record. Insights from the TMOS ARtO pilot informed the establishment of a new Career Reconnect Fellowship offered to three women in 2021, allowing these women further upskilling, connection and opportunity for reestablishment of their research career.

We also saw positive uptake of ARtO in the large recruitment campaign for excellence in research. Following the implementation of ARtO and Special Measures, we have seen greater diversity in applicants than previous years. Out of all 2021/22 appointments, 76% identified as female (she/her). Out of all successful applicants, 41% utilised ARtO demonstrating its uptake and its utilisation in appointments. Inclusive of this data is the appointment of one Aboriginal and Torres Strait Islander Fellow.

RMIT is now developing a consistent ARtO employee experience, with adaptations for academic promotion and recruitment, as part of a phased implementation. Key considerations include further education for those applying ARtO for increased uptake where appropriate and reinforcing the cultural change required for everyone to feel safe in its application.

## A29 What will it take: gender equality in leadership by 2030. Collaborating internationally and across sectors to Transform Women's Leadership Pathways

### Lily Halliday

#### University of New South Wales, Sydney, Australia

##### **Correspondence:** Lily Halliday (lily.halliday@unsw.edu.au)

*BMC Proceedings 2023*, **17(15):**A29

The inaugural Transforming Women’s Leadership Pathways (TWLP) event in 2020 brought higher education, industry and government partners together to develop action plans to close the gender leadership gap across 10 sectors by 2030. This was the first time a group of top international universities (the PLuS Alliance) collaborated with industry and government to tackle this global challenge.

Representatives from 10 sectors engaged in working groups focussed on the delivery of an action plan for their specific area. The 10 groups were the Arts, Corporate, Engineering, Entrepreneurship & Innovation, Higher Education, Media & Communications, Medicine, Policy & Politics, Science, and Technology. The action plans articulate the practical steps universities, industry and government must take to see more women in leadership by 2030. Building on previous initiatives, working groups tested recommendations against the guiding principles of intersectionality, sustainability and the changing nature of work to create solutions that are inclusive and adaptive for future ways of working. To ensure the recommendations put to universities, industry and government were evidence-based and inclusive, each working group was designed to include a diverse group of people from multiple institutions and professional backgrounds. Pre-reading packs specific to each area and general resources around women’s leadership were circulated to participants to provide a starting point for best practice research, programs, policies, processes and systems. Experts provided custom case studies and participated in the groups. A Student Ambassador program was developed to engage students from each of the PLuS universities. This allowed students to connect and network with leading academics, policymakers, and professionals across each sector.

An event website served as a key part of the initiative's success. It stored supporting resources including session guidelines, technical support, action plan templates, pre-reading, case studies, programs and timetables, event support and contact information, as well as a networking lounge.

The 10 action plans have been published, and we are working individually and in concert with partners from higher education, industry and government to implement the recommendations to drive workplace change and to close the gender leadership gap by 2030. Transparency and the tracking of our progress and impact is key to our program design, and outcomes will be shared on our website.

This novel approach would not have been possible without the buy-in from our passionate working group members, and our leaders. Progress requires a commitment from all stakeholders, and the leveraging of networks and advocates. As we move forward, we have developed processes and pathways to ensure our community is supported in their efforts to shift the dial, with both people resources and via schemes to fund the implementation of action plan recommendations.

## A30 RCSI staff and students collaborate to develop Ireland's first Race Equality Action Plan in the higher education sector

### Oluchi Porter

#### RCSI University of Medicine and Health Sciences, Dublin, Ireland

##### **Correspondence:** Oluchi Porter (oluchiporter@rcsi.ie)

*BMC Proceedings 2023*, **17(15):**A30

**Background**

In January 2021, the RCSI University of Medicine and Health Sciences convened a Race Equality Forum (the Forum) as part of the University's commitment to advancing race equality for staff and students. The Forum is comprised of a multidisciplinary and diverse group of 42 students and staff, with the support and sponsorship of RCSI’s Senior Management Team. The Forum is joined by valued external advisors, including Pavee Point Traveller and Roma Centre and Advance HE Race Equality Charter award holders in the UK.

RCSI is a multicultural university with staff and students hailing from more than 80 countries. However, 82.2% of Ireland’s overall population identify as white Irish [1]. Therefore, RCSI’s work on race equality is cognisant of the dynamics of being a multicultural institution in a society where minoritised ethnic communities experience discrimination and exclusion. The Forum is also informed by the findings in Ireland’s Higher Education Authority 2021 report detailing the experiences of staff relating to race equality [2]. This report was the first of its kind and highlighted the structural racism and discrimination that exists in Irish higher education institutions. Until 2022, higher education institutions were not required to collect staff ethnicity data.

The Forum was tasked with developing RCSI’s first three-year Race Equality Action Plan, the first of its kind for any higher education institution in Ireland, and provides an opportunity to take meaningful steps to effect change and to evaluate the impact and effectiveness of change initiatives. Adopting an intersectional approach was a fundamental aspect to this project and initiatives within the action plan will contribute to Beacon projects to RCSI’s Athena Swan Action Plan.

**Methods**

The Race Equality Action Plan was developed through a consultative process with 42 volunteer Forum members that represented a diverse group of nationalities, ethnicities, seniority levels and lived experiences. Facilitated conversations on inclusive conversations and organisational change were provided including a training on race equality and on Irish Traveller rights.

Members were divided into seven different workstreams while working together to develop an evidence-based action plan relevant to the RCSI context. The workstreams focused on governance and culture, data, policies and procedures, awareness-raising and training, education, reporting, and recruitment. During the development phase, the Forum and individual workstreams collaborated to create a comprehensive and considerate action plan (18 consultations in total).

**Results**

A three-year action plan was developed and launched in November 2021 at a student–staff partnership event, involving a panel discussion with female student leaders of colour, race equality experts and senior staff.

**Conclusions**

The RCSI Race Equality Action Plan [https://bit.ly/35YxuKS], which aims to address race equality for both staff and students, offers valuable best practice insights and learning for the entire Irish sector. The plan is currently being implemented.

**References**

1. Central Statistics Office [Internet]. Cork (Ireland): Central Statistics Office. Census of population 2016 – profile 8 Irish Travellers, ethnicity and religion. Available from: https://www.cso.ie/en/releasesandpublications/ep/p-cp8iter/p8iter/p8e/
2. Kempny S, Michael L. Race equality in the higher education sector. Dublin (Ireland): Higher Education Authority; c2021. 110 p.

## A31 "Make Visible": promoting diversity and inclusion for members of gender and sexually diverse communities (to create a 'Ripple Affect') – in partnership with community and industry

### Amy B Mullens^1*^, Shannon Novak^2^, Beata Batorowicz^3^, Annette Bromdal^3^, Brodie Taylor^3^

#### ^1^University of Southern Queensland, Ipswich, Australia; ^2^Independent consultant, Auckland, New Zealand; ^3^University of Southern Queensland, Toowoomba, Australia

##### **Correspondence:** Amy B Mullens (amy.mullens@usq.edu.au)

*BMC Proceedings 2023*, **17(15):**A31

**Background**

Make Visible is an ongoing, community-led project developed by New Zealand artist, curator and activist Shannon Novak and is aligned with his work currently featured in the Queensland Art Gallery and Gallery of Modern Art (QA GOMA) Asia Pacific Triennial-10. The aim of this initiative is to grow support for the LGBTQI+ community worldwide by making visible challenges and triumphs for this community. This project seeks to address the following: 1) Reduce rates of anxiety, depression and suicide in the LGBTQI+ community; 2) Create positive, meaningful and sustained change for the LGBTQI+ community; 3) Centre and amplify LGBTQI+ BIPOC (Black, Indigenous, People of Colour) and Brotherboy/Sistergirl voices; 4) Collect, archive and share LGBTQI+ history; and 5) Increase awareness and knowledge of the LGBTQI+ community.

**Methods**

This project included detailed formative assessment and ongoing community participation and co-design, process evaluation and formal research and evaluation phases. These included ethnographic, auto-ethnographic, qualitative and action research paradigms, as well as practice-led frameworks, including non-traditional (artistic) research outputs. Further, artworks and arts contributions made by/for the exhibition enabled new discoveries to emerge through the art-making process and in its completion.

**Results**

Formative assessment commenced with the artist (SN) approaching a researcher and clinical/health psychologist (AB) to explore ways in which art could be used as a dissemination tool for sharing research findings with new (and non-academic) audiences to positively influence social change. This led to a submission of works for the QAGOMA APT-10 and subsequent “Artist In Residence” program at the University of Southern Queensland, with various branching projects and initiatives creating a “ripple affect”, including (but not limited to): art making/reflection workshops for students and community members; university-based art exhibition opening/panel discussion and curators talks; impetus for a diversity and inclusion research symposium; creation of artistic works surrounding contemporary research (e.g. chemsex, trans incarceration); the launch of a LGBTIQA+ health and wellbeing needs survey; and connections forged with LGBTIQA+ community members, artists, academics/researchers, students, clinicians and industry partners—including the Curious Arts Festival. A research ethics application has been progressed alongside “Make Visible” to conduct the transformational and lived experiences of those involved in 1) the process of organising the project/exhibition; 2) creating/curating artistic works for the project/exhibition; and 3) viewing/participating in the events/workshops.

**Conclusions**

This project is meaningfully and effectively raising awareness of the unique strengths and challenges experienced by members of rainbow communities through art as a medium—sparking various other rippling projects to sustain the project’s mission and grow momentum and reach across community, industry and academic settings—through various learning, teaching, research and service initiatives. This project provides a useful template for bridging art with science to work towards enduring positive changes within society among seemingly disparate (yet interconnected) groups. This model can be readily adapted to other contexts and priority groups to enable and promote advocacy, awareness-raising and much needed social change.

## A32 Studying and parenting: supporting student mothers during COVID-19 and beyond

### Anne Jamison, Dorothea Bowyer, Chloe Taylor, Tinashe Dune, Erika Gyengesi, Milissa Deitz, Jaime Ross, Anita Ogbeide, Hollie Hammond

#### Western Sydney University, Sydney, Australia

##### **Correspondence:** Anne Jamison (a.jamison@westernsydney.edu.au)

*BMC Proceedings 2023*, **17(15):**A32

**Background**

Substantial progress has been made in academic institutions across Australia in enhancing gender equity for academic staff and reducing barriers to career progression, especially for women and mothers. However, less attention has been given to students in this context, especially student mothers. This is despite a growing body of research that indicates student mothers in Australia and elsewhere invariably commence their studies with complex and significant obstacles to learning which include their role as carers [1,2]. Preliminary research and media reports further suggest that the global COVID-19 health crisis has exposed a lack of institutional support for student mothers and heightened their existing challenges. This paper seeks to outline the experiences of student mothers currently studying at university before, during and after the peak of the COVID-19 health crisis in Australia, as well as better understand the gendered and other challenges they face in achieving equal participation and success in their studies.

**Methods**

This paper’s discussion is based on a case study carried out at a large regional university in Australia. The study utilised two qualitative methods for exploring the experiences of student mothers at the university. This paper’s discussion and results are based on an online Qualtrics survey issued to student mothers at the university, as well as three online focus groups. Both the survey and focus group methodology employed open-ended questions to encourage depth of response.

**Results**

The findings of the study coalesced around several core emerging themes, which this paper outlines and discusses. Each theme reveals and evidences a series of significant gendered barriers to equal and effective participation in study for student mothers, including a number of key pressure points for this cohort: identity and recognition, time management, social isolation, wellbeing, impacts of COVID-19, and flexibility. Significantly, the study further identified existing positive support models and staff behaviours and attitudes at the university that assisted student mothers in their study and helped to mitigate some of the worst effects of the gendered barriers they face.

**Conclusions**

Our paper concludes that the negative impacts of COVID-19 have been disproportionately felt by student mothers engaged in university study in Australia. Our core findings and discussion suggest that the fragile life–work–study balancing act of student mothers has long suffered a lack of institutional recognition and support, and that the global pandemic has further aggravated the complex circumstances surrounding these students and their efforts to engage in higher education. This has had severe consequences for the study progression of this cohort. Our paper finally recommends that we need to include student voices in gender equity conversations and formal action planning in order to better support student mothers into the future.

**References**

1. Stone C, O’Shea S. Older, online and first: recommendations for retention and success. Australasian Journal of Educational Technology. 2018;35(1):57-69.
2. Chesters J, Watson L. Returns to education for those returning to education: evidence from Australia. Stud High Educ. 2014;39(9):1634-48.

## A33 LIBRA: future proofing gender equality in higher education institutions – experiential learning in simulation for student leaders

### Claire Condron^1*^, Siobhán Lucey^2^, Siobhán Lucey^2^, Patrick Henn^2^, Michelle Scott^1^, Avril Hutch^2^, Tanya Dean^3^, Míde Power^1^

#### ^1^Royal College of Surgeons in Ireland, Dublin, Ireland; ^2^University College Cork, Cork, Ireland; ^3^Technological University Dublin, Dublin, Ireland

##### **Correspondence:** Claire Condron (ccondron@rcsi.ie)

*BMC Proceedings 2023*, **17(15):**A33

**Background**

A recent study highlighted the gender inequality that exists in student leadership, demonstrating that although females made up 55% of the student body, they represented only 33% of the leadership in student organisation [1]. This gender imbalance is replicated in senior leadership in Irish higher education institutions [2]. Training is needed to increase awareness and nurture competencies for student leaders to actively address gender inequality. The LIBRA programme aims to empower student leaders to promote gender equality among the student body and in the wider higher education community. By working with leaders of the future, this project strives to future-proof gender equality in Irish higher education leadership and accelerate culture change. This project is a multi-institutional initiative, involving partnership with the Royal College of Surgeons in Ireland, University College Cork and Technological University Dublin.

**Methods**

A mixed-method approach was adopted to capture the perspectives and ideas of relevant stakeholders to develop an evidence-based and collaborative programme on gender equality training. Six focus groups were carried out with 29 student leaders, discussing participants’ experiences and perceptions of gender and gender equality as a student in higher education. Nine interviews were carried out with experts in gender equality and leadership. The interviews focused on experts’ experience of promoting gender equality and leadership and considered the core competences required to foster gender equality. Student leaders across the three partner higher education institutions were also invited to participate in a quantitative survey to determine the status of the gender equality agenda present in undergraduate and postgraduate courses. Qualitative data was reviewed and synthesised using framework analysis, and descriptive statistics were used to draw conclusions from the quantitative survey data.

**Results**

The LIBRA gender equality leadership training programme pilot will be completed in 2022.

**Conclusions**

Findings from the empirical data collection will be employed to develop the key blended learning components of the LIBRA gender equality leadership training programme. This will take the format of in-person, experiential simulation interactions which will include pre-encounter content, group-learning interactions with simulated participants, peer observation and feedback and post-encounter reflection. LIBRA will apply the principles and practice of peer-based learning to teach and assess skills that address gender equality and raise awareness of inclusive leadership behaviours. Potential scenarios may include: bystander interventions; difficult one-to-one conversations; navigating power dynamics in promoting gender equality and facilitating inclusion of gender diversity. Students will participate in scenarios, both as the protagonist interacting with trained role players and then as an observer, and give feedback to their fellow students.

**References**

1. Handayani T, Widodo W. Gender gaps in students leadership at a university in Portugal. Advances in Social Science, Education and Humanities Research. 2019;349:578-81.
2. Higher Education Authority. Higher education institutional staff profiles by gender. Dublin (Ireland): Higher Education Authority; 2020. 40 p.

## A34 Towards a better minority/gender representation in Australian higher education: the case of Asian female academics

### Nana Oishi^1*^, Guangyu Qiao-Franco^2^

#### ^1^University of Melbourne, Parkville, Australia; ^2^University of Southern Denmark, Odense, Denmark

##### **Correspondence:** Nana Oishi (nana.oishi@unimelb.edu.au)

*BMC Proceedings 2023*, **17(15):**A34

The report, Workforce Diversity in Higher Education [1], identified major challenges that Asian academics face in Australian universities, including: (1) their significant under-representation in senior positions, despite the fact that Asians have constituted the majority of international students since the 1980s; (2) the gender gap among Asian-born academics has widened between 2005 and 2015, while it has been closed for other groups; and (3) the majority (67.9%) of Asian women felt they had been affected by their immigrant background in the workplace, which was higher than the figure (52.7%) for their male counterparts.

This research argues for more attention to intersectionality in diversity and inclusion (D&I) efforts to address these issues. Based on 77 qualitative interviews with Asian female academics, it identified several interventions that have been and/or would be helpful in addressing their under-representation and challenges. The most crucial one is the university management’s commitment to diversifying the staff and supporting minorities. When there is a strong commitment from senior management, more minorities are hired, and support is provided for inclusion. Unconscious bias training is also effective, as studies have demonstrated that unconscious bias often operates in recruitment/promotion processes. Such training should be offered more widely, together with anti-racism and intercultural-awareness training. Training alone, however, will not resolve the under-representation of Asian women: quotas and/or targets, which successfully advanced women’s positions in Australian academia, should also address intersectionality.

The participants acknowledged that Asian women tend to undervalue themselves and do not feel comfortable with self-promotion due to their cultural upbringing. They also struggle with their family’s gender role expectations. For these matters, mentoring is helpful, though its effectiveness varies, depending on the program arrangements, mentors’ time commitment and the nature of mentorships. Long-term, regular consultations and research collaboration with mentors are effective. For those who were educated in Australia, their academic advisors often support them as informal mentors, providing advice and career-related opportunities. Those who were educated overseas, however, struggle to find supportive mentors due to their limited social capital in Australia. Their lack of cultural capital and unfamiliarity with Australia’s higher education system add another challenge. Official mentorship is crucial for them, but some participants argued that mentors should be incentivised in the forms of workload and/or remunerations, as they tend to be too busy to meet with their mentees on a regular basis. Others reported that the mentoring/leadership programs that specifically cater to minority women were highly helpful. The participants benefitted from developing connections with women in senior management, receiving advice from them and developing support groups with other minority women. Such targeted programs should become more widely available. Lastly, the participants emphasised the importance of minority female “role models” in their university communities. Australian universities should have more diverse ethnic and gender minorities in visible leadership positions to inspire future generations of minority academics.

**Reference**

1. Oishi N. Workforce diversity in higher education: the experiences of Asian academics in Australian universities. Melbourne (Australia): The University of Melbourne; 2017. 60 p.

## A35 Impact of parenthood on career progression in STEMM

### Isabel L Torres^1*^, Victoria Sugrue^2^, Mira Rahal^3^, Adeline Samson^4^, Elise Arnaud^4^

#### ^1^Mothers in Science, Montpellier, France; ^2^University of Otago, Dunedin, New Zealand; ^3^Paris School of Economics, Paris, France; ^4^University of Grenoble, Grenoble, France

##### **Correspondence:** Isabel L Torres (isabel.torres@mothersinscience.com)

*BMC Proceedings 2023*, **17(15):**A35

**Background**

Despite decades of efforts to close the gender gap in science, technology, engineering, maths and medicine (STEMM), women are still vastly underrepresented in this sector, particularly in leadership positions – including in fields where women outnumber men as graduates [1]. Thus, attracting more women into STEMM careers is insufficient to close the gender gap and more research efforts should be invested into developing effective interventions to increase retention. Evidence shows that implicit gender bias partially explains the so-called STEMM "leaky pipeline” [2]. However, another contributing factor has received less attention: parenthood. The negative impact of work–family conflict on the career and wellbeing of mothers is well reported, but few studies have addressed this question in STEMM. A recent US study shows that parenthood is an important driver of gender imbalance in STEM, with 42% of mothers leaving full-time STEM employment after becoming parents [4]. However, the mechanisms of mothers attrition remain poorly understood. Research analysing the average publication rates of American and Canadian scholars suggests that parenthood explains the widely reported gender productivity gap in academia [5], yet it remains unclear why parenthood impacts on scientific productivity and why only mothers seem to be affected.

**Methods**

We conducted a global survey to measure the impact of parenthood on career progression in STEMM. Statistical descriptive and regression analyses were performed to identify the mechanisms promoting the exit of mothers from STEMM careers.

**Results**

About 9,000 STEMM professionals from 128 countries completed the survey. Based on self-reported average publication rate, our data show there is a significant scientific productivity gap between mothers and fathers in every academic field analysed. By using a Poisson mixed-effects model, we found that women working in Physical Sciences experience a significant reduction in scientific productivity after becoming parents. Moreover, our data shows there is widespread maternity discrimination in STEMM. Mothers were more likely than fathers to say they were perceived as being less competent, and were offered fewer professional opportunities, since becoming a parent. Importantly, using a general linear model we found a significant interaction between fewer offered opportunities and lower publication rates for mothers. Taken together, our data suggest that maternity discrimination is an important obstacle to the career advancement of women in STEMM by indirectly impacting on scientific productivity.

**Conclusions**

Our research shows that structural barriers related to parenthood contribute to the underrepresentation of women in the STEMM sector, and thus has important implications for the design of policies and interventions to retain women in STEMM careers.

**References**

1. Shannon G, Jansen M, Williams K, Cáceres C, Motta A, Odhiambo A, Eleveld A, Mannell J. Gender equality in science, medicine, and global health: where are we at and why does it matter? Lancet. 2019;393(10171):560-9.
2. Lerchenmueller MJ, Sorenson O. The gender gap in early career transitions in the life sciences. Research Policy. 2018;47(6):1007-17.
3. Mason MA, Wolfinger NH, Goulden M. Do babies matter? Gender and family in the ivory tower. New Jersey: Rutgers University Press; 2013.
4. Cech EA, Blair-Loy M. The changing career trajectories of new parents in STEM. Proc Natl Acad Sci U S A. 2019;116(10):4182-7.
5. Morgan AC, Way SF, Hoefer MJD, Larremore DB, Galesic M, Clauset A. The unequal impact of parenthood in academia. Sci Adv. 2021;7(9):eabd1996.

## A36 Impact of health-related factors on gender inequality: a case study of OECD countries

### Vipul Sharma, Shivani Mehta, Gargi Sharma

#### Amity University, Noida, Uttar Pradesh, India

##### **Correspondence:** Vipul Sharma (thevipulsharma19@gmail.com)

*BMC Proceedings 2023*, **17(15):**A36

The United Nations Development Programme (UNDP) asserts that gender inequality remains a major blockade to human development. This in turn poses strong challenges for global economies in their drive towards attaining Sustainable Development Goals. Therefore, this study analyses the impact of health-related factors on gender inequality especially among OECD countries, using the Gender Inequality Index (GII) developed by UNDP. GII measures three key components – reproductive health, labour market participation, and empowerment – to create an index primarily concentrated on gender inequality, especially highlighting women. As a result, the higher the GII value, the greater the disparities between men and women, and the greater the loss of human development. Descriptive and panel data regression analysis are performed to examine the impact of gender inequalities in health on economic productivity using statistical and econometrics tools. The study finds an inverse relation between gender inequality and life expectancy where 'disability' is seen as a significant barrier to gender equality. Therefore, the study concludes by putting forward key recommendations for supporting gender equality, as it is essential for augmenting national development and instrumental in attaining the Sustainable Development Goals.

## A37 Promotion support initiatives in the Faculty of Science and Engineering at Anglia Ruskin University

### Claudia A F Wascher, Christopher Parris, Helen Keyes

#### Anglia Ruskin University, Cambridge, United Kingdom

##### **Correspondence:** Claudia A F Wascher (claudia.wascher@aru.ac.uk)

*BMC Proceedings 2023*, **17(15):**A37

The ‘leaky pipeline’ metaphor describes how women and other disadvantaged groups become underrepresented throughout their academic careers. In the Faculty of Science and Engineering at Anglia Ruskin University, in 2017/18, 43% of undergraduate students were women, whereas they were virtually absent from Grade 8 (Faculty Management and Professor) and above. To address this and, in time, establish a good gender balance in senior positions, several support initiatives were introduced.

First, we have rolled out a ‘promotion spreadsheet tool’. The spreadsheet tool provides an opportunity for members of staff to self-evaluate career progression standing and get feedback from their line manager. All academics are encouraged to discuss the spreadsheet tool with their line manager during the annual appraisal process and it is encouraged to formulate yearly objectives around areas for development identified during these discussions.

Second, an ‘ambitious women’ peer-support group was formed creating a safe environment for a small number of peers to discuss and encourage applications for promotions. The ‘ambitious women’ groups are locally run in some areas of the Faculty and are organically self-organised based on community initiative.

Third, on the basis of the success of these local groups, we developed ‘promotion clubs’ across all areas of the Faculty. The purpose of these clubs is to provide peer support and discuss, share and review applications for promotion. The spreadsheet tool and promotion clubs are centrally advertised to all members of staff by the Dean and the Athena SWAN self-assessment-team chair respectively. Clubs are led by experienced members of staff, selected via an Expression of Interest process, and receive a time allocation in the workload allocation model.

All three support initiatives received positive feedback. People said they appreciated the space and time the clubs provided to them and said it allowed them to work on their applications. They also positively acknowledged having some space where it was OK to be openly ambitious. People also gave feedback that they applied for promotion earlier than they would have done without the additional support. Staff appreciated the clarity of the spreadsheet tools and said it helped them to easily evaluate which promotion criteria they already meet and to identify developmental needs. Line managers also reported that the spreadsheet tool helped them to initiate conversations around promotion.

Success of support initiatives is also reflected in successful promotion application from members of staff from our Faculty. In the 2020/21, six women and three men have been promoted from Lecturer to Senior Lecturer, 10 women and one man have been promoted from Senior Lecturer to Associate Professor and two women and one man were promoted from Associate Professor to Professor. Overall, we feel that the support initiatives helped to create a culture of open conversation around the promotion process within our Faculty. As well as having a positive effect on career progression, these initiatives have had a positive impact on wellbeing; members of staff feel supported in their career ambitions and line managers feel they can support their staff.

## A38 Intersectional and place-based barriers to women’s advancement in higher education

### Gail Crimmins^1*^, Sarah Casey^1^, Maria Tsouroufli^2^

#### ^1^University of the Sunshine Coast, Sunshine Coast, Australia; ^2^Brunel University, London, United Kingdom

##### **Correspondence:** Gail Crimmins (gcrimmin@usc.edu.au)

*BMC Proceedings 2023*, **17(15):**A38

**Background**

Evidence of persistent barriers to women’s advancement in higher education are well-documented. In response, over the past two decades, many Certification and Award schemes (CAS) related to gender equality, diversity and inclusion have emerged in the higher education, research, and industry sectors. The Athena Swan Charter “is arguably the most prominent and well-known certification system for research organisations” [1]. The Athena Swan Charter was established in 2005 to combat the gender inequities in higher education and research careers faced by women in science, technology, engineering, maths and medicine (STEMM). The Charter’s remit expanded in 2015 to encompass gender equality in academia and research in the fields of arts, humanities, social sciences, business and law. As of July 2021, there were 962 active awards in total, with 164 held by institutions and 798 by departments. Notwithstanding the significant role that Athena Swan plays in higher education institutions’ gender equity policy and practice, the perceived impact of Athena Swan upon these work experiences, remains under-researched and theorised.

**Methods**

We therefore designed a research project where academics and professional staff employed in across 12 schools or units within academic institutions in the UK and Northern Ireland were invited to provide their demographic details and to describe their work experience and career opportunities.

**Results**

A total of 207 respondents participated in the survey from various institutions across England, Scotland, Northern Ireland and Wales. Quantitative findings revealed that even in higher education institutions that have been awarded (and thus recognised) for supporting gender equality, a higher proportion of males were: employed in a permanent/ongoing basis; more likely to be promoted, and promoted within a shorter timeframe, than females; more likely to hold a senior professorial and leadership role than females; and less likely to spend 40 hours of more on caring responsibilities than females. Furthermore, Black, and Asian participants were found to have been employed for under 5 years. The recurrent themes that emerged in the qualitative data were: women experience the role of primary carer as a barrier to career success; a gendered allocation of tasks/workload prevent access to promotion; a lack of engagement with, and appropriation of, female ‘voice’; and the intersection of religion and gender can reinforce gendered and raced structures.

**Conclusions**

Analysis of these findings illuminate that, despite the implementation of gender equity plans, raced and gendered power structures within higher education institutions are maintained through ‘everyday sexism’ [2] and further suggest that the geographical contexts of higher education institutions not only reflect but appear to constitute intersectional experience. The outcomes demonstrate a multiplicative effect of race/religion and parental responsibility on career opportunities for women, reinforces the need for data collection and analysis that is sensitive to intersectional social identities, and recommendation to use a place-based lens to focus on social locations rather than groups in research and policy development.

**References**

1. Tzanakou C, Clayton-Hathway K, Humbert AL. Certifying gender equality in research: lessons learnt from Athena SWAN and Total E-Quality award schemes. Front Sociol. 2021 Nov 16;6:784446.
2. Savigny H. Women, know your limits: cultural sexism in academia. Gend Educ. 2014;26(7):794-809.

## A39 Decadal Plan Champions addressing gender equality

### Anna-Maria Arabia, Zach Ghirardello

#### Australian Academy of Science, Canberra, Australia

##### **Correspondence:** Zach Ghirardello (zach.ghirardello@science.org.au)

*BMC Proceedings 2023*, **17(15):**A39

The Australian Academy of Science (the Academy) is spearheading the development of critical national frameworks for diversity and inclusion to guide transformative, systematic and sustained change in Australia’s STEM sector. In collaboration with the Australian Academy of Technology and Engineering, the Academy co-authored the Women in STEM Decadal Plan, a 10-year strategy to lift the participation of girls and women in STEM education and careers. The plan outlines six opportunities for stakeholders—government, academia, industry, education, and the broader community—who have the power to achieve gender equity by 2030. As the steward for the plan, the Academy coordinates the Women in STEM Decadal Plan Champions initiative enabling STEM sector organisations to publicly align their initiatives to achieve gender equity with the six opportunity areas detailed in the Plan: leadership and cohesion; evaluation; workplace culture; visibility; education; industry action.

Over 40 organisations have become Decadal Plan Champions over the last 3 years. Their gender equity actions provide a source of ideas and inspiration for everyone wishing to support girls and women in STEM. Aligning actions with the Plan means the Australian STEM sector can push in the same direction to achieve gender equity by 2030.

All Champions display visible leadership from their executive teams and demonstrate efforts to improve the visibility of women in STEM roles. The adoption of varying forms of inclusive workplace practices such as flexible work, paid parental leave policies, domestic violence leave, and training are common. Women earn 12.4% less than their counterparts and pay equity remains a persistent challenge for the sector.

This abstract showcases the efforts and successes of four Champions from different industries. Analysis of other actions and gaps will be presented orally.

Opportunity 2: Evaluation – QinetiQ conducts an annual gender pay gap analysis conducted using the Workplace Gender Equality Agency (WGEA) gender pay gap calculator, and reports openly on their progress towards gender quality, including an increase in the representation of women in their executive leadership team from 25% to 40% and an increase in the overall recruitment ratio of women from 19.5% to 23%.

Opportunity 3: Workplace culture – Department of Defence has collaborated with Defence industry partners in a women’s mentoring program called ‘The Future Through Collaboration’, which has provided 284 women working in Defence and Defence Industry with an opportunity for mentorship that may otherwise not have been available.

Opportunity 4: Visibility – Macquarie University has established gender equity targets in their marketing strategy and now undertakes regular reporting, resulting in an increase in female representation to 45% in internal and external media channels.

Opportunity 5: Education – CSIRO developed the Young Indigenous Women’s STEM Academy which in 2021 is supporting 308 young Indigenous women across Australia to engage with a range of STEM experiences virtually throughout COVID-19.

To achieve gender equity, it is vital that long-term and sustainable changes are implemented across the STEM sector, which is why initiatives such as the Women in STEM Decadal Plan and Champions are instrumental.

## A40 MI TALENT – The Herschel Programme for Women in Technical Leadership

### Lucy Williams^1^, Denise McLean^2*^

#### ^1^MI TALENT – University of Nottingham, Nottingham, United Kingdom; ^2^University of Nottingham, Nottingham, United Kingdom

##### **Correspondence:** Denise McLean (denise.mclean@nottingham.ac.uk)

*BMC Proceedings 2023*, **17(15):**A40

Midlands Innovation (MI) TALENT is a Research England-funded programme which leads and influences change to advance status and opportunity for technical skills, roles and careers in UK higher education and research. One of the programme workstreams focuses on equality, diversity and inclusion, addressing challenges identified by data analysed across the UK technical workforce.

The Herschel Programme for Women in Technical Leadership is a pilot programme, designed by TALENT in collaboration with staff development colleagues across the eight MI universities. It aims to provide a dedicated career development opportunity to address the lack of women in technical leadership positions across the sector [1,2].

The Herschel Programme provides a space for women technicians to learn new skills to develop themselves in a current technical leadership role or equip them if they aspire to be in the future. The programme is named after Caroline Herschel, a pioneer in the discovery of comets and other astronomy work, assisting her brother William. Caroline was an early ‘technician’ at the turn of the 19th century and paved the way for the women of the future to contribute to and play key roles in scientific endeavours.

Following consultation with current women technical leaders and staff development colleagues across the MI partnership, the structured six-month programme offering has developed modules on the topics of You as a Leader, Context and Culture, Influencing and Negotiating, and Confidence and Empowerment.

The Herschel Programme is an innovative programme developed to address a specific challenge evidenced by sector data. It also has an important technician lens placed on women leadership development, often not fully considered in generic institutional-led programmes. There are over 180 delegates on the Herschel Programme, the first of its kind in the UK. It began in January 2022 and will culminate in a celebration event in July 2022. As a pilot programme, MI TALENT is taking steps to evaluate the programme and analyse continuous delegate feedback.

**References**

1. STEMM-CHANGE. Equality, diversity and inclusion: a technician lens. Nottingham (UK): University of Nottingham; 2019. 9 p.
2. MI TALENT. The TALENT Commission report. Nottingham (UK): University of Nottingham; 2022 Feb. 75 p.

## A41 Developing, implementing and embedding gender identity and diversity training and champion network for staff in Irish higher education institutions

### Aine Coady^1^, Kenneally Allison^2*^

#### ^1^Institute of Technology Carlow, Carlow, Ireland; ^2^Waterford Institute of Technology, Waterford, Ireland

##### **Correspondence:** Kenneally Allison (allison.kenneally@wit.ie)

*BMC Proceedings 2023*, **17(15):**A41

**Background**

The aim of this project was to tackle gender inequality in the partner higher education institutions (HEIs) through development of a multifaceted training programme to drive change using interactive learning sessions for staff. The project was funded by the Irish Higher Education Authority through its Gender Equality Enhancement Fund and involved 5 partner HEIs committed to combatting stereotyping and unconscious bias in relation to sexual orientation and gender identity minority groups, fostering gender balance and invoking inclusion for the LGBTQ+ community within the HEIs.

**Methods**

Training was developed in conjunction with and delivered by ShoutOut, a sector-leading charity working to create inclusive services and educational institutions. Training modules included: Bias, Gender & Sex, Differentiations, Gender Stereotypes, Expansive Look at Gender, Identity Explainers, Language (Pronouns & Gender-neutral Language), LGBTQ+ People in Higher Education (including the Ward–Gale Model for LGBTQ+ Inclusivity in Higher Education). Tailored workshops aimed at staff in Senior Leadership, HR and general HEI staff were developed and piloted.

**Results**

Following the initial pilot training sessions, staff were asked to give anonymous feedback on the training. 77 responses to the feedback request were received and the answers were overwhelmingly positive (Table 1). All participants reported that the training was relevant to their work. Qualitative feedback was complimentary of the delivery method, the relaxed nature of the presentation, and the use of lived experience as well as being clear and informative. Points of criticism were around operational issues such as interactivity, time for Q&A etc. These points were taken into account in refining the course content in future.

**Conclusions**

Feedback demonstrates the clear appetite amongst those in the sector for bespoke training in this area. Further funding has been secured for the roll out additional training to enable the HEIs to deliver training in-house in the future and to have access to permanent online resources to support training and policy development initiatives. The partners also launched a National Gender Diversity Champions Network which will be accessible for all staff in Irish HEIs and will act as a forum for collaboration and support, to share best practice and for policy development. The founding and development of the Project Steering group has been invaluable to the partners. In the second year the group has widened to include six HEIs including four campuses of the new Technological Universities. The Steering Group has provided the foundation for the Gender Diversity Champions network, which will be opened to all interested in the HE sector in Ireland. The involvement of the training partner ShoutOut has also provided vital support, credibility and a source of information to the partners.

**Table 1 (abstract A41).** Feedback from pilot programme participants across partner HEIs

The training was...	Comprehensive	Easy to understand	Engaging	Interesting
Strongly agree	40	49	48	53
Agree	35	24	24	23
Neutral	1	4	4	1
Disagree	1	0	0	0
Strongly disagree	0	0	0	0

## A42 Shared parental leave – are higher education institutions encouraging the conversations needed to challenge culture assumptions about who cares?

### Clare Matysova^1,2*^

#### ^1^London School of Hygiene and Tropical Medicine, London, United Kingdom; ^2^University of Leeds, Leeds, United Kingdom

##### **Correspondence:** Clare Matysova (bncmat@leeds.ac.uk)

*BMC Proceedings 2023*, **17(15):**A42

UK’s Shared Parental Leave (SPL) aims to give parents more choice in relation to caring during their child’s first year [1]. Through this potentially increased choice, SPL has possibilities to reduce the impact of maternity leave on women’s career continuity and progression, create greater opportunities for fathers to engage with their children and for children to be cared for by both parents [2]. However, since its introduction in 2015, take up of SPL has been low and this is often attributed to parental choice, for example women not wanting to share their leave, as well as to financial barriers and concerns over the potential impact on fathers’ careers [3]. Lack of awareness and complexity of the policy are also presented as explanatory factors [4] as well as continuing cultural gendered parenting expectations [5].

Employers play a key role in terms of both policy entitlements and workplace culture, how family leave options are communicated and more broadly how carers are supported. While some employers have proactively led the way in encouraging sharing of leave, there has been limited employer focus on family leave, and SPL in particular. Reflecting on the university sector, many higher education institutions offer relatively good financial incentives with soon after implementation approximately 65% of responding institutions offer enhanced SPL, of which 92% were matching maternity pay [6]. However, despite this relatively high proportion of enhanced SPL pay, a review of institutional Athena SWAN gender equality action plans suggests often tokenistic and limited university-led proactive actions to increase take up. Given the significant impact that women taking more time out to care has on the gender pay gap [7], this is a missed opportunity. While finances may be a significant influence, organisational culture also contributes. Research on the role of policy and culture in relation to carers in higher education suggests, especially for academic roles, that care work is often rendered invisible [8].

The aim of this presentation is to spark conversations that challenge the common focus on financial barriers, challenge cultural assumptions about who carers are and creates a more visible approach to supporting parenting, and shared parenting, within higher education institutions. The presentation should be of interest to those working to close the gender pay gap as well as considering the role of Athena SWAN in promoting family leave. There will be a focus on the role of policy and entitlement as well as culturally the steps a university can take. The objective will be to raise the profile of the importance of employer support for expectant and new parents particularly in the current uncertain circumstances and, in this context, to also raise consciousness regarding the potential for SPL to both support parents and to promote gender equality.

**References**

1. GOV.UK [Internet] New ‘Share the joy’ campaign promotes shared parental leave rights for parents; 2018 February 12. Available from: https://www.gov.uk/government/news/new-share-the-joy-campaign-promotes-shared-parental-leave-rights-for-parents
2. Kurowska A, Javornik J. Comparative social policy analysis of parental leave policies through the lenses of capability approach. In: Yerkes MA, Javornik J, Kurowska A, editors. Social policy and the capability approach: concepts, measurements and application. 1st ed. Bristol: Policy Press; 2019. p. 83-106.
3. Twamley K, Schober PS. Shared parental leave: exploring variations in attitudes, eligibility, knowledge and take-up intentions of expectant mothers in London. J Soc Policy. 2018;48:387-407.
4. Ndzi E. Challenges of shared parental leave: reasons why mothers may/may not want to share their maternity leave. SSRN. 2018 Jun 20. Available from: https://ssrn.com/abstract=3191034
5. Birkett H, Forbes S. Where’s dad? Exploring the low take-up of inclusive parenting policies in the UK. Policy Stud. 2019;40(2):205-24.
6. Equality Challenge Unit. Improving uptake of shared parental leave: guidance for UK higher education institutions and colleges in Scotland. London (UK): Equality Challenge Unit; 2018. 15 p.
7. Government Equalities Office. Gender equality at every stage: a roadmap for change. London (UK): Government Equalities Office; 2019 July. 28 p.
8. Moreau M-P, Robertson M. ‘You scratch my back and I’ll scratch yours’? Support to academics who are carers in higher education. Soc Sci (Basel). 2019;8(6):164.

## A43 Advancing women's representation in top academic positions – the influence of institutional measures

### Charlotte Silander^1*^, Ida Drange^2^, Maria Pietiälä^3^, Liza Reisel^4^

#### ^1^Linnaeus University, Växjö, Sweden; ^2^Oslo Metropolitan University, Oslo, Norway; ^3^University of Eastern Finland, Kopio, Finland; ^4^Institute of social research, Oslo, Norway

##### **Correspondence:** Charlotte Silander (charlotte.silander@lnu.se)

*BMC Proceedings 2023*, **17(15):**A43

**Background**

The share of women in top academic positions remains well below the threshold for gender balance in the Nordic countries [1]. This is the case even after a long history of progressive national legislation, universal systems for work–family reconciliation, laws that require employers to take proactive measures to improve the career opportunities of both women and men and comprehensive regulation directed towards the universities [2]. In this paper, we ask what Nordic higher education institutions have done at the institutional level to address gender inequalities in academic careers. Which measures have universities used to increase gender equality, and to what extent are the measures effective in increasing the share of women in top academic positions? This is the first study in the Nordic context that tries to assess which equality measures work, using quantitative methods.

**Methods**

Based on theories on actor-oriented and structure-oriented measures, we investigated the efficacy of gender equality policy measures in 37 universities in Sweden, Norway and Finland, implemented between 1995 and 2018 based on interviews with universities’ HR staff and equality officers. The study combines survey data and register data to assess the impact of institutional gender equality policies on the gender composition of academics in grade A positions. By combining unique survey data on universities’ equality policies and register data on universities’ teaching and research staff, we assess the impact of the policies on the gender composition of academics in grade A positions (e.g. professor positions). We distinguish between career-enhancing measures offered for women, training and awareness-raising measures, organisational responsibility measures and preferential treatment measures.

**Results**

Overall, we find that the use of equality measures has increased over time, but that equality measures seem to contribute relatively little to the overall share of women in grade A positions between 1995 and 2020. Using regression models with fixed effects and lagged information on the introduction of the various types of measures across the 37 institutions, we find that only the structural measures stand out as significantly associated with an increase in the share of women in grade A positions. In particular, having an equality officer or office and providing hiring support for recruitment of women seem to be positively associated with an increase in the share of women in grade A over time.

**References**

1. European Commission. SHE figures 2018. Directorate-General for Research and Innovation. Luxembourg: Publications Office of the European Union; 2019 Mar.
2. Lipinsky A. Gender equality policies in public research. Luxembourg: Publications Office of the European Union; 2014.

## A44 Decolonising London South Bank University – development of a contextualised evidence-informed reflective checklist wheel

### Musharrat J Ahmed-Landeryou

#### London South Bank University, London, United Kingdom

##### **Correspondence:** Musharrat J Ahmed-Landeryou (ahmedlmj@lsbu.ac.uk)

*BMC Proceedings 2023*, **17(15):**A44

**Background**

Decolonising the curriculum is a whole-university transformation, disrupting colonial legacies in knowledge sharing, production and dissemination [1]. London South Bank University (LSBU) has started its decolonising curricula process. The author was awarded a teaching fellow for academic year 2021–2022 for a practice innovation project, developing an evidence-informed framework to guide decolonising curricula for allied health professions (AHP) courses at LSBU, as existing frameworks were not explicit regarding their evidence base. Decolonising the curriculum is not ‘one size fits all’ methodology, it is contextual to the discipline and university [2].

**Methods**

A scoping literature review and meta-thematic analysis of 12 focus group reports regarding students’ experiences of belonging, racism and discrimination at university were carried out.

The scoping literature review question: *What are the components of decolonising the curriculum checklist and why?* The term AHP was removed as this only yielded one paper.

Inclusion criteria: Peer-reviewed from 2015, the year of the #RhodesMustFall movement. Exclusion criteria: No English translation. The thematic analysis followed Popay et al.’s guidance [3].

**Results**

Thirty-two papers identified from the review, 20 included, 18 subthemes identified and grouped into 4 categories (Table 1). The 12 reports yielded 32 subthemes, grouped into 8 categories, 4 were duplicates of the scoping literature review (Table 1). The structural shape used is a wheel, it is simple but holds complex information, and is symbolic of continuity [4]. The linchpin of the wheel is category f); the spokes of the wheel are categories a) to d), g) and h); the wheel rim is category e); and the tyre holds four helpful prompt questions to then be able to plan actions.

**Conclusions**

The AHP decolonising curricula reflective checklist wheel is not prescriptive; it is a prompt to explore areas. This should be in collaboration with students and with the invested support of the university. We must disrupt the status quo of institutional racism and discrimination in higher education. Decolonising the curriculum is a way forward, not an endpoint.

**References**

1. Abu Moghli M, Kadiwal L. Decolonising the curriculum beyond the surge: conceptualisation, positionality and conduct. London Review of Education. 2021;19(1):1-16.
2. Keele University. Decolonising the curriculum: staff guide. Staffordshire (UK): Keele University; 2021 Feb. 43 p.
3. Popay J, Roberts H, Sowden A, Petticrew M, Arai L, Rodgers M, Britten N with Roen K, Duffy S. Guidance on the conduct of narrative synthesis in systematic reviews. London: ESRC Methods Programme; 2006 Apr. 92 p.
4. Hopkins R. Picture, image and experience: a philosophical enquiry. Cambridge: Cambridge University Press; 1999.

**Table 1 (abstract A44).** Categories identified from scoping literature review and thematic analysis of focus group reports

Overlapping categories	Only categories from meta-analysis
a) Decolonising pedagogies	e) Be impactful not performative
b) Decolonising topics/subject content	f) Centre students’ voices in designing and changing
c) Decolonising assessments	g) Decolonising assessment feedback
d) Institutional responsibility in decolonising curricula	h) Outcome measures of impact of decolonising the curriculum

## A45 Equity in the Canada Research Chairs Program

### Kaleb Saulnier, Marie-Lynne Boudreau

#### Tri-agency Institutional Programs Secretariat, Ottawa, Canada

##### **Correspondence:** Marie-Lynne Boudreau (marie-lynne.boudreau@sshrc-crsh.gc.ca)

*BMC Proceedings 2023*, **17(15):**A45

The Canada Research Chairs Program (CRCP) was established at the turn of the millennium by the Government of Canada and created 2000 research chair positions. Up to $311 million per year has since been invested to recruit some of the world’s best researchers to Canada. Chairholders conduct research in engineering and the natural sciences, health sciences, the humanities and social sciences.

Since its inception, the program has faced equity challenges. In 2016, the program launched an equity, diversity and inclusion (EDI) action plan in response to recommendations made in its 15^th^ year evaluation. The plan required participating institutions to meet equity targets by December 2019, develop their own EDI action plans, and meet increased public accountability and transparency requirements.

Between 2016 and 2022 representation in the program increased significantly; representation of women went from 28.9% to 40.9%, racialised minorities from 13% to 22.8%, persons with disabilities from 0.59% to 5.8% and Indigenous Peoples from 0.95% to 3.4%. Institutions that did not meet the deadline for their equity targets were restricted to submitting new nominations only where it addressed an equity gap. As part of a 2006 Canadian Human Rights Settlement agreement, for which an important addendum was signed in 2019, the program committed to ensuring that its representation [1] is reflective of Canada’s population by 2029 [2]: 50.9% women, 22% racialised minorities, 7.5% persons with disabilities and 4.9% Indigenous Peoples. Data is also being collected to monitor nomination rates from the LGBTQ2+ community so that further best practices can be implemented.

All institutions with five or more chair allocations (55/78 institutions) were required to develop their own EDI action plans, which were formally peer-reviewed [3] by an external panel in April 2019. Plans that did not receive a ‘satisfies’ rating (38/55) were required to revise and resubmit their plans for a second evaluation in March 2020. In alignment with the program’s consequences framework [4], institutions not receiving a satisfactory rating in the second review process were restricted to only submitting new nominations that contribute towards their equity targets. In addition, peer review results and payments for all nominations were withheld until the plan was found to meet requirements.

As of December 2018, all institutions were given strict recruitment and nomination requirements focusing on EDI best practices and increased transparency and accountability. In addition, all institutions are required to publish information on their public-facing website that explains how they manage their allocation of chairs in order to further improve transparency and accountability [5].

As of April 2022, 86% (56/65) of institutions are meeting their December 2019 equity targets and 95% (52/55) of institutions have an EDI action plan that meets requirements. Consequences continue to be imposed when requirements are not met.

Engagement activities are planned for 2022 to gain a better understanding of how the program and institutions can build on these experiences and continue increasing the level of EDI in the program. In this session, we unpack the lessons learned to date. For progress towards our equality targets, please see annual updates on the CRCP representation statistics [1].

**References**

1. Canada Research Chairs [Internet]. Canada Research Chairs; [updated 2022 Nov 16]. Equity targets and results of participating institutions. Available from: https://www.chairs-chaires.gc.ca/about_us-a_notre_sujet/statistics-statistiques-eng.aspx#4
2. Canada Research Chairs [Internet]. Canada Research Chairs; [updated 2022 Sep 29]. Establishing equity targets for 2021 to 2029. Available from: https://www.chairs-chaires.gc.ca/program-programme/equity-equite/targets-cibles-eng.aspx
3. Canada Research Chairs [Internet]. Canada Research Chairs; [updated 2022 Jun 28]. Results of formal review of institutional equity, diversity and inclusion action plans. Available from: https://www.chairs-chaires.gc.ca/program-programme/equity-equite/results_of_formal_review-resultats_de_l_evaluation_officielle-eng.aspx
4. Canada Research Chairs [Internet]. Canada Research Chairs; [updated 2023 Apr 6] Consequences framework for institutions that do not meet the program’s equity, diversity and inclusion (EDI) requirements. Available from: https://www.chairs-chaires.gc.ca/program-programme/admin_guide-eng.aspx#equity
5. Canada Research Chairs [Internet]. Canada Research Chairs; [updated 2023 Apr 20]. Institutional public accountability web pages. Available from: https://www.chairs-chaires.gc.ca/program-programme/equity-equite/Institutional-etablissements-eng.aspx

## A46 Bridging the gap – harnessing local and central resourcing to increase impact and efficiency in Athena SWAN activities across departments and the institution

### Kevin S M Coutinho, Sara E Mole

#### UCL, London, United Kingdom

##### **Correspondence:** Kevin S M Coutinho (coutinhok@windsor-fellowship.org)

*BMC Proceedings 2023*, **17(15):**A46

**Background**

A workshop exploring impact of increased cross-institution Athena SWAN (AS) working and co-ordination to increase efficiencies, share learning and mitigate AS fatigue, thereby improving gender equality. With 43 departmental awards including three Golds, there are multiple institutional/departmental AS self-assessment teams (SAT) at UCL focused on developing/delivering AS submissions/action plans. Much work is duplicated, with similar issues/initiatives that could be more impactful with scale/coordination. The 2021 institutional Silver submission committed every academic department and professional services area to hold an award by 2026. Furthermore, with increased specialisation in AS work and SAT membership turnover, knowledge and expertise are lost, exacerbating burden and inefficiencies.

**Methods**

Coordination/partnership efforts have focused on:

1. Data production – providing AS data consistently was difficult particularly because of institutional expansion, data management system limitations (with these not being aligned to produce AS submission data/information) and data accuracy (data integrity). Dedicated Data Analysts posts were created, managed by the UCL Athena SWAN Manager, who processed data and developed data sets for departments. Data integrity issues started to be addressed by new data management systems, information flagging the impact of inaccurate data, sharing good practice on data presentation and modelling better data AS practices, including intersectionality guidance.
2. Sharing information on practice – by developing networking through termly Athena Forums; workshops (AS surgeries); internal SharePoint providing detailed submission guidance and sharing successful submissions; coordinated AS governance through the Gender Equality Steering Group.
3. Co-ordination of local staffing and co-management – faculties/departments were encouraged to recognise the workload needs and resource posts (either within departments or faculty). To coordinate and guide AS work, management support through the UCL Athena SWAN Manager was offered. A Central Athena SWAN Team was developed, providing common purpose for local AS staff; meeting fortnightly to deliver and co-ordinate AS activities.
4. Developing UCL Internal Mock Panels (IMPs) – an internal process was introduced, offering feedback on draft submissions and providing volunteer panellists training on what ‘good’ submissions looked like, with learning used in preparing their own submissions. Given limited availability of national Advance HE panel positions, this enabled sharing of the skills and experience of UCL’s Advance HE panellists with 95 UCL staff members sitting on IMPs.
5. Broader EDI engagement activity – the overall approach linked to institutional efforts to increase engagement and recognition of all equality work at faculty/department levels, including: reconfiguration of governance committees; appointment of institutional Equality Envoys, faculty EDI Vice-Deans and faculty Athena SWAN leads; development of faculty EDI/Athena SWAN committees; development of local initiatives, including funding for EDI/Athena SWAN projects.

**Results**

AS engagement and outcomes have improved. Since its introduction, the IMP process has reduced the number of unsuccessful UCL submissions. In 2018, 1 out of 9 submissions secured the award applied for; by 2020/21, 12 out of 13 did so.

**Conclusions**

Whilst workload issues and associated frustrations remain, greater recognition through promotions criteria and dedicated staffing has mitigated these. Together, these activities enable greater focus on realising gender equality.

## A47 The future of gender equality action plans: Athena Swan and the European GEP requirement

### Charikleia Tzanakou, Anne Laure Humbert

#### Oxford Brookes University, Oxford, United Kingdom

##### **Correspondence:** Charikleia Tzanakou (ctzanakou@brookes.ac.uk)

*BMC Proceedings 2023*, **17(15):**A47

Creating gender and diversity change in institutions relies on devising appropriate interventions, though it is important to understand the wider context in which this process of change is taking place. The landscape has changed considerably over the past two years, following the announcement by the European Commission that Gender Equality Plans (GEPs) would become a requirement for applying for European funding from 2022. For UK institutions, this new development means that the question of how to respond to both the requirements of setting plans of interventions that respond to both Athena Swan and the GEP eligibility criterion is a particularly relevant one.

To showcase the breadth of possible gender and diversity interventions, as well as provide inspiration for future ones, we draw upon EU-funded gender equality and structural change projects where gender and diversity interventions have been designed, implemented and evaluated. We showcase structural change projects such as PLOTINA and GEARING-Roles, in which we have been involved, and also refer to examples and practices from sister projects that have developed a wealth of resources regarding diversity interventions such as GE Academy or FESTA. Because gender and diversity interventions are typically part of wider certification or award schemes, understanding the architecture of this wider frame matters to understand how these schemes (and the interventions within them) might evolve. To illustrate the relevance of the architecture of gender and diversity-related schemes, we present the recent CASPER project, a feasibility study on a potential Europe-wide certification or award system which was based on extensive certification mapping and needs assessment to develop scenarios for a system that can lead to transformative and sustainable change. The CASPER project identified and mapped 113 certification and awards schemes, together with extensive fieldwork and consultation to learn from existing certification and award schemes. This was used to develop four scenarios that could be used as a basis for a future Europe-wide scheme on gender equality, including one scenario looking at the Europeanisation of Athena Swan. All scenarios are underpinned by considerations of how gender inequalities intersect with other inequalities (race, ethnicity, social background, ability, nationality and other social relations.

Using these examples, we aim to encourage participants to reflect on the wider purpose of gender and diversity interventions, and how gender and diversity change agents will need to shape interventions in the future to ensure alignment between ongoing Athena Swan work and the new opportunities under the GEP requirement. We discuss the potential arising from a potential Europe-wide scheme for enhancing gender and diversity efforts, not only for meeting funding requirements but more importantly about how organisations can benefit from sharing practices and establishing a Europe-wide community of experts and change agents and tools to support change. Thus, this presentation is of interest to senior leaders, researchers and equality, diversity and inclusion practitioners.

## A48 Working towards the creation of an inclusive curriculum: a staff–student partnership approach

### Angie Makri, Stephanie McDonald

#### University of Nottingham, Nottingham, United Kingdom

##### **Correspondence:** Angie Makri (angie.makri@nottingham.ac.uk)

*BMC Proceedings 2023*, **17(15):**A48

Decolonising the curriculum is moving fast at the top of the higher education agenda. Individuals, however, often struggle to identify where to start and what approach to take in their teaching practice to implement positive change. Using an evidence-based approach, we developed a model around decolonising the psychology curriculum, involving undergraduate and postgraduate students as co-creators of the curriculum, taking an equality, diversity and inclusion perspective. As part of this project, a group of students reviewed and evaluated diversity in one of our first year modules, Developmental Psychology. Following this, module changes were implemented based on the group’s feedback, focusing on active learning methods. Further, student feedback on the new module activities was obtained using a novel impact evaluation measure. Here we present key features of this approach, as well as insights on impact of change using a novel measure we developed as part of this project. We will share our reflections and some practical suggestions for teaching practice, from the student perspective, within the context of an active approach to learning.

## A49 Using the British Council’s comprehensive global “Maximising Impacts” report to improve actions on gender equality and leadership in your institution

### Helen L Mott^1,2*^

#### ^1^Independent Consultant, Bristol, United Kingdom; ^2^The British Council, London, United Kingdom

##### **Correspondence:** Helen L Mott (helen@helenmott.com)

*BMC Proceedings 2023*, **17(15):**A49

**Background**

Gender equality and the empowerment of women and girls is central to the work of the British Council as a cultural relations organisation that promotes equality, diversity and inclusion as core values. In March 2022, the British Council published a comprehensive global report on “Gender Equality in Higher Education: Maximising Impacts” [1].

**Methods**

Using the academic and grey literature, and interviews with practitioners around the world, this report asks the questions: what is the role of HE in transforming society in relation to women’s equality and empowerment, and how is gender inequality reflected, reinforced and challenged worldwide?

**Results**

For the first time, research and best practice from around the world that addresses the multiple manifestations of gender inequality in higher education has been collated together into one research document. Gender inequality is manifested from subject selection to the curriculum, teaching and learning environments, in research and innovation, and in the threat and reality of violence against women staff and students. Men as a group remain advantaged at every stage of their academic careers. This report highlights research data, searchable by country and region, across multiple areas. The importance of intersectionality, compounded disadvantage and taking a lifecycle approach, are highlighted. Seventeen case studies – including Athena Swan – examine successful programmes and interventions, some dedicated to gender equality and others seeking to mainstream it, from policies and frameworks to actions with individual students or teachers. The report provides a compendium of resources and examples to help with gender mainstreaming, covering policy and systems development; institutional partnerships; professional development; student mobility; insight, analysis and advocacy. The resources include suggestions for gender-focused activities and for how to monitor and evaluate.

**Conclusions**

In order to maximise impacts for gender equality the following recommendations are made: 1) prioritise gender mainstreaming; 2) develop gender expertise; 3) ensure an intersectional approach; 4) put a greater focus on violence against women; 5) address women’s under-representation in HE leadership; 6) tackle subject segregation, particularly in STEM; 7) take a gendered approach to online learning and collaboration; 8) strengthen organisational leadership and commitment to address gender inequality in strategy, policy, quality assurance and delivery; 9) recognise and promote gender studies and women’s higher education institutions; 10) take a lifecycle approach; 11) assert the centrality of equality and inclusion to the definition of quality and excellence in higher education; 12) act at scale. This report is designed to assist all those working for gender equality in higher education to maximise their impact.

**Reference**

1. Mott H. Gender equality in higher education: maximising impacts. British Council 2022/M024; 2022 Mar. 128 p.

## A50 How GEPs can help EU-funded STEM projects in adding a gender+ approach to communication and dissemination activities

### Rita Bencivenga^1*^, Cinzia Leone^1^, Anna Siri^1^

#### ^1^University of Genoa, Genoa, Italy

##### **Correspondence:** Rita Bencivenga (rita.bencivenga@unige.it)

*BMC Proceedings 2023*, **17(15):**A50

**Background**

Numerous basic research projects in STEM fields funded by the EU do not yet adopt a cross-cutting approach regarding gender and diversity. This presentation discusses an experimental action [1] included in area 4, "Integrating the gender dimension into research and teaching content", of a Gender Equality Plan (GEP) created based on the European Institute for Gender Equality’s Gender Equality in Academia and Research tool. The action intends to 1) facilitate scientists’ and researchers’ understanding of what the GEP requires of the entire academy, experimenting with micro-actions for the project's tasks and deliverables; 2) understand the numerous strategies that could be adopted to address sex and gender in basic research projects as well.

Horizon Europe, the European Union research and innovation funding program 2021–2027, introduced new gender-related requirements, which extend what was already requested under the previous program, Horizon 2020. They include the requirement that the gender dimension be adopted for the entire research and innovation process as well as a gender+ strategy, which considers how gender interacts with other sources of inequality and discrimination, where possible by adopting intersectional indicators. The presentation focuses on a case study, ongoing activities in the Communication and Dissemination Work Package for a research project financed by Horizon Europe on "surface patterning", i.e. techniques for preparing and patterning surfaces from the arrangement of single molecules to the macro-scale.

**Methods**

The case study includes two sets of activities related to gender+, which will be carried out by a team comprising three gender experts: a) co-lead communication activities to comply with the modern understanding of engendered science and b) a survey collecting gender+ disaggregated data during outreach tasks at Science Festivals in five countries.

**Results**

The activities implemented so far address set a), “engendering” the Dissemination, Communication and Exploitation (C&D) plan, through strategies that embed references to the initiative in all communication and dissemination outputs. The aim is to spread information about sex/gender perspectives adopted in literature in the field, and learn about relevant resources (e.g. the SAGER guidelines and the Brussels Binder leaflets). Moreover, a textual analysis of the proposal identified specific terminology that might trigger references to gender+ in describing the project activities and in outlining the use other scientists may make of the project results (e.g. “societal benefits”, “biomedicine”). The partners will be offered advice clinics on the links between these words/concepts and sex/gender and intersectionality.

**Conclusions**

This initiative’s relevance is linked to its easy replicability, as C&D plans have very similar contents and structures in EU projects. The results will help partners implement further research with a gendered perspective, make the project an “ambassador” for engendered science and better understand the direct links a GEP may have to their research and teaching activities. The planned monitoring and evaluation activities will help achieve the desired impact.

**Reference**

1. Bencivenga R, Siri A, Leone C. Project_Gender Action Plans in academia. In: Proceedings of the 5th International Conference on Gender Research. Aveiro: University of Aveiro; 2022:43-51.

## A51 Setting up inclusive Gender Equality Plans - the CALIPER experience

### Kyriaki Karydou^1*^, Maria Sangiuliano^2^, Vicky Moumtzi^1^, Danai Kyrkou^1^, Marzia Cescon^2^

#### ^1^VILABS, Thessaloniki, Greece; ^2^Smart Venice, Venice, Italy

##### **Correspondence:** Kyriaki Karydou (kyriaki-karydou@vilabs.eu)

*BMC Proceedings 2023*, **17(15):**A51

**Background**

The adoption of a Gender Equality Plan (GEP) aims to remove the systemic obstacles to gender equality and adapt institutional practices, leaving no one behind [1]. However, there is a need for a renewed approach to the development of a GEP which is reflected in the European Commission’s new policy direction which is going towards a more inclusive approach. This new direction refers to “inclusive GEPs”, covering 3 aspects: intersectionality, intersectoriality, and geographic inclusiveness [1]. The CALIPER project, which supports nine Research Performing Organisations (RPOs)/Research Funding Organisations (RFOs) to develop a GEP, was designed and is now implemented, having these dimensions at each core and especially intersectoriality which is a key specific feature embedded in all steps of the institutional change process. Elaborating on these dimensions, our aim is to present below the CALIPER methodology and provide practical examples for setting up inclusive GEPs.

**Methods**

CALIPER's methodology for setting up inclusive GEPs [2], adopting a quadruple/multiple helix and gender-sensitive approach to innovation ecosystems, included an analysis of external [3] and internal [4] conditions for the GEP development and acceptance. During this process, potential gender biases and inequalities identified along with scenarios towards change. A “GEP Working Group” has been set up including staff members at different managerial levels including stakeholders from middle and high management. Qualitative and quantitative gender analysis has been performed as an initial and preliminary step: intersectional and inter-sectoral indicators for collecting data and relevant targets have been proposed to conduct internal assessment of gender+ inequalities and to map and analyse external innovation ecosystems with gender lenses and using mixed-methods, including Social Network Analysis. Despite the challenges met in the operationalisation of intersectional and inter-sectoral indicators on gender in research and innovation, the devised methodology has led each RPO/RFO to set up their own Research & Innovation Hubs, including stakeholders from academia and universities, industry, ministries/government, public sector, civil society organisations via the organisation of dedicated dialogues on exchanging knowledge and promoting joint actions on gender equality in research and innovation.

**Results**

A co-creation process running in parallel with both internal and selected external actors has led to the design of tailored inclusive GEPs based on each partner’s unique realities. While the plans focus on generating internal sustainable change, they include collaborative initiatives in synergy with external actors: the purpose is to promote and support gender equality inward at the CALIPER partner institutions, while having an outward and multiplying effect at the territorial level. The recommended areas for action for an RPO/RFO based on CALIPER’s experience include Human Resources, Institutional Governance, Institutional Communication, Student Services, Teaching and Research, Sexism and Sexual Harassment, Intersectionality and Transfer to Market.

**Conclusions**

CALIPER demonstrates how inclusive dimensions can be incorporated into the GEP design and development phase, working on specific focus areas and with a gradual approach. Through this process, the institutions have now approved and published their GEP, and work on the implementation of the envisaged activities. 

**Acknowledgements**

The CALIPER project has received funding from the European Union's Horizon 2020 Research and Innovation programme under Grant Agreement No 873134.

**References**

1. European Commission. Horizon Europe guidance on gender equality plans. Luxembourg: Publications Office of the European Union; 2021. 54 p.
2. CALIPER: gender equality in STEM research [Internet]. Project vision: linking research and innovation for gender equality. Available from: https://caliper-project.eu/project-vision/
3. Sangiuliano M, Cescon M, Nason G. D1.3 Gender Analysis of research and innovation ecosystems and reports from the local R&I hubs. Zenodo; 2021 Jul 13. 250 p. Report No.: Ares(2020)73164. Supported by Horizon 2020.
4. Moumtzi V. D1.2: Internal Gender Equality Assessments Results. Zenodo; 2021 Jul 13. 162 p. Report No.: Ares(2020)72537. Supported by Horizon 2020.

## A52 UCL COVID-19 Career Support Scheme: an intervention to reduce the impact on staff career development from the COVID-19 pandemic

### Kevin S M Coutinho, Sara E Mole, Peter H Holmes

#### UCL, London, United Kingdom

##### **Correspondence:** Kevin S M Coutinho (coutinhok@windsor-fellowship.org)

*BMC Proceedings 2023*, **17(15):**A52

**Background**

A workshop to explore different responses to mitigate the impact of COVID-19, identify good practice and lessons learned that could be retained and used to inform future Equality, Diversity and Inclusion (EDI) policy development. In March 2020, as the pandemic’s first wave in the UK hit, it was clear lockdown measures would disproportionately adversely impact some groups more than others. Evidence from a UCL EDI survey and roundtable events on gender equality identified these groups to be parents, carers, women and fixed-term contract staff, especially researchers. In response, the £600,000 COVID-19 Career Support Scheme was developed. The aim was to mitigate adverse impact caused by the pandemic that resulted in lost work productivity and that may cause longer-term career harm.

**Methods**

The scheme was divided into three streams. 1) *Giving Back Time (GBT)* – provided up to £500 to offer a short-term boost to work capacity by alleviating other personal pressures, such as extra child-related expenses. 2) *Equity Bridging Fund (EBF)* – provided substantial grants (≤£10,000) to support recipients’ work, including salary costs via contract extensions or increased hours for part-time staff. 3) *Supporting Teaching, Technical, Research, Academic and Professional Services (STTRAP)* – funded UniTemp workers to support UCL staff with their work. Either 20 or 40 hours of support was available.

**Results**

There were six application rounds to the scheme between November 2020 and February 2021. Each application was reviewed against established criteria. To be successful, applicants needed to demonstrate: disruption caused by COVID-19; disruption was equity and inclusion based; disruption had the potential for long-term career detriment; funding was in scope of the scheme. 221 staff applied to the scheme (1.5% of all UCL staff), with 151 applicants (69%) successful. The most popular stream was GBT (50% applications), followed by EBF (40%) and STTRAP (10%). Successful applicant profile: 45% research staff; 93% parents and/or carers; 75% women (52% white women and 21% BAME women); 29% Black, Asian or other minority ethnic; 13% disabled.

The outcomes and impact:

*GBT* –- 81 staff received an average payment of £486.90. The funding was predominantly used for childcare (95%), freeing an average of 37 working hours per person. 91% gained a short-term boost to work capacity. 79% managed to do work that otherwise would not have been done.

*EBF* – 53 staff gained on average an additional 39 working days to their contract. 90% managed to do work that otherwise would not have been done. 75% thought the scheme would mitigate some of the negative impacts of COVID-19 on their career.

*STTRAP* – 17 staff received 660 hours of UniTemp support. 93% gained a short-term boost to their work capacity. 86% managed to do work that otherwise would not have been done.

**Conclusions**

The scheme provided short-term boosts to recipients, with a significant proportion reporting improved mental health and feeling supported by UCL. The extra time gained allowed staff to write papers and grant applications, submit promotion applications, and carry out vital teaching and administrative tasks – all critical to career development.

## A53 A good practice design for a large decentralised research organisation: the case of the “Support Programme Equal Opportunities” within the Fraunhofer-Gesellschaft

### Clemens Striebing^1*^, Katharina Scharrer^2^, Katrin Mögele^2^, Katharina Hochfeld^1^, Martina Schraudner^1^

#### ^1^Fraunhofer IAO, Berlin, Germany; ^2^Fraunhofer-Gesellschaft, Munich, Germany

##### **Correspondence:** Clemens Striebing (clemens.striebing@iao.fraunhofer.de)

*BMC Proceedings 2023*, **17(15):**A53

**Background**

The Fraunhofer-Gesellschaft in Germany is the world's largest organisation for applied research. More than 30,000 staff members work in 76 institutes and research facilities throughout Germany. Typically for many STEM-oriented organisations, the proportion of women researchers is low. By 2020, the proportion of women researchers increased by four percentage points to 24% compared to 2016. As the size and decentralised organisational structure result oftentimes in complex and lengthy change processes, Fraunhofer created the ***“****Support Programme Equal Opportunities****”*** (May 2021 to March 2022) to promote gender equality. The programme’s goal is threefold: 1) accompany the Fraunhofer institutes to implement equal opportunities through a strategic approach and new measures as well as methods on the ground; 2) promote the achievement of the equality policy objectives agreed with the Federal state; 3) foster a comprehensive culture change through the dissemination of the centrally organised development programme on gender equality in the Fraunhofer Institutes.

**Methods**

The programme serves as an innovative approach recognised as good practice by the new edition of the EIGE GEAR tool. Coordinated by the central Diversity Management, the programme has the following characteristics. 1) The *coordinating department* collaborates with various departments, such as personnel marketing, recruiting or executive search. External experts support the activities, e.g. workshop design and moderation. The coordinating department organises a *workshop series* for a community of appointed institute representatives to enable participants to develop *local strategy plans* for the promotion and implementation of equal opportunities at their institutes. The *transfer of knowledge* on strategic equal opportunities management and existing funding and networking opportunities within Fraunhofer is established. 2) The institute representatives create *local steering committees,* consisting of decision-makers, personnel and equal opportunities managers from their institute, to develop and implement institute-specific local strategic plans. 3) The implementation is regularly and periodically *evaluated* during workshops and peer groups. A summative evaluation is carried out by the end of the implementation phase.

The *"Support Programme Equal Opportunities"* is based on the Community of Practice concept, tailoring all measures to the contextual conditions of a large, decentralised research organisation. It follows a *community-based approach* as participation is voluntary and anchored in peer-to-peer assessment. Even though the process is coordinated centrally, top-down, it is supported exclusively by the bottom-up commitment of the institutes, thus establishing a *multi-level approach,* resulting in an otherwise unattainable area effect.

**Results**

38 of 76 institutes participated in the workshops of the support programme. In a final survey (n = 29), half of the respondents could see progress in small steps and 20% confirmed an overall good development, compared to one tenth, seeing no progress at all; three quarters set up a team to implement equal opportunity actions; three out of five institutes are in the process of planning or implementing measures.

**Conclusions**

The initial results suggest that a process of change has been initiated in many institutes. The support programme will be continued to strengthen these positive developments in the future.

## A54 Celebrating 'age and stage’ gender equality interventions

### Jill Childs, Susan A Brooks

#### Oxford Brookes University, Oxford, United Kingdom

##### **Correspondence:** Jill Childs (jchilds@brookes.ac.uk)

*BMC Proceedings 2023*, **17(15):**A54

During work towards renewal of our Departmental Silver award, we have focused on creation of two separate, but related, diversity interventions which exemplify empathy in action. Our ‘Who cares?’ initiative evolved from the pressures of the COVID-19 pandemic where in the face of lockdowns and the requirement for home-working, those many staff with caring responsibilities – particularly those with caring responsibilities for children and/or elders – faced substantial additional pressures. At the same time, the unprecedented situation fast-tracked the evolution of carer-friendly flexible institutional working practices.

The lack of visibility associated with caring roles and the associated hidden costs for individuals is broadly acknowledged [1]. Building on the results of an internal survey, which clearly indicated that agile working improved flexibility options for staff with, often unacknowledged, caring roles, we are now able to provide guidelines to better support the needs of employees managing dual roles, including caring. Our work is underpinned by The Women's Higher Education Network’s Survey [2], which highlights how women who self-identify as belonging to dual-career households are predominantly responsible for caring-related duties.

Our work adds to the ‘care-focused feminism’ and ‘ethics of care’ paradigms, which outline how society should value caregivers and give recognition in public and private spheres [3] and sits within institutional work for the Athena SWAN award. In addition, we recognise that, as a Faculty where approximately 70% of the staff are women, including around 70% of the senior – and therefore, generally, older – staff, menopause is, in addition to caring, a major challenge affecting employees and, therefore, the productivity and effectiveness of the organisation. Our ‘menopause project’ has established a menopause café and online forum, and will encompass a cross-university awareness-raising menopause event, the development of webpages and establishment of a mentorship model, and is also focused on effective and empathetic policy change. Based on survey data and focus group feedback, plus a map of inclusive questions to inform practice, we are developing an organisational menopause policy and working to embed menopause awareness within and across Equality, Diversity and Inclusion and Healthy Ageing research networks. The project will develop an infographic/poster campaign that will seek to raise awareness across the University. Finally, looking forwards and recognising the issue of resourcing as a barrier to effective change, we are further developing an innovative ‘Athena Allies’ initiative as a creative solution to continue the momentum and impact of our Athena SWAN work.

**References**

1. Advance HE [Internet]. Generating cultural change for a more inclusive HE sector: students and staff with caring responsibilities. York (UK): Advance HE; 2019 Oct 2. Available from: https://www.advance-he.ac.uk/news-and-views/generating-cultural-change-more-inclusive-he-sector-students-and-staff-caring
2. Chalmers IV. Sharing the caring: UK Higher Education Professional Services parents, work and family life during 2020 lockdown. Bicester (UK): Women’s Higher Education Network. 34 p.
3. Held V. The ethics of care. New York: Oxford University Press; 2007.

## A55 The Roving Researcher Scheme at Babraham Institute: an innovative intervention mitigating the impact of long-term leave on life science research and researchers

### Cheryl D Smythe^1,2*^, Melanie Stammers^1^

#### ^1^Babraham Institute, Cambridge, United Kingdom; ^2^Altos Labs, Cambridge, United Kingdom

##### **Correspondence:** Cheryl D Smythe (csmythe@altoslabs.com)

*BMC Proceedings 2023*, **17(15):**A55

As a small business-owner in the 1980s, my Dad and I had animated discussions about employment of women: “what’s the business going to do when they go off to have a baby?” he would ask. It was a fair question and 40 years on, researchers still ask related questions. At Babraham we have often provided cover for those in management roles on leave, but cover is not usually sought for researchers, many choosing to add on the unused grant time at the end. So while time is not always lost, the project stalls between the ramping down and ramping up. Additional challenges arise when research cannot just be put ‘on ice’.

Organisations sometimes provide additional finance to those on leave to ease the impact, but repeated recruitment of temporary researchers to fill these gaps is costly in terms of time and money. Babraham Institute has instead developed an innovative intervention – the Roving Researcher Scheme, where a highly skilled and permanently employed post-doctoral Rover maintains the momentum of research projects while researchers are on leave – maternity-, paternity- or sick-leave. While only running for two years, it has already supported nine researchers [1].

Importantly, the Rover does not replace those on leave; rather, in advance of that leave, they develop an experimental plan together to maintain research momentum. This enables the Rover to support a number of projects at any one time. A reciprocal handover then happens at the end of the period of leave.

Excellent organisational skills are essential for anyone in this unique role together with equally good communication skills; it is a job for a true team player. In return, the Rover acquires a wide range of skills and expertise in different scientific areas which leaves them incredibly well equipped for future research roles, and as they rove, they impart novel approaches and ideas across labs.

The Rover has not only supported those on leave, but also the labs of junior group leaders who struggled to recruit during the pandemic, and core facilities, to relieve backlogs due to absences during this time. A number of requests for support have unfortunately had to be turned down resulting in the Institute recently funding an additional post, which, together with other organisations, we hope will form a community of Rovers.

Indeed, many other organisations have contacted us interested in developing similar schemes [2]. We have collated their questions in an FAQ to support others [3]. Additional resources available include the job description and advertisement, interview questions and the governance and process behind this initiative.

While this scheme has reduced the impact of long-term leave on research and researcher careers, we hope for wider impact, for example an increase in uptake of paternity leave or providing access to those who, for whatever reason, cannot access the lab. Ultimately this scheme is part of our aspiration to create an environment where the most talented researchers can contribute their skills and expertise irrespective of their personal circumstances – a true meritocracy.

**References**

1. Wynn E. A tale of two Melanies: mitigating the impact of lab long term leave [Internet]. Babraham Institute Blog; 2021 Jan 08. Available from: https://www.babraham.ac.uk/blog/2021/01/tale-two-melanies-mitigating-impact-lab-long-term-leave
2. @cdws100. Delighted to see that @MRC_LMS are also looking to reduce the impact of long-term leave from the lab by recruiting a Roving #researcher! Great opportunity for London-based #LifeSciences researcher to learn lots of new skills and work with great scientists. imperial.ac.uk/jobs/description/MED03082/research-associate. [Twitter]. 2022 Mar 28. Available from: https://twitter.com/cdws100/status/1508390938867081216?s=20&t=eT-oKPDlMI092lNig_xzDg.
3. Alumni Blogger. Roving Researcher FAQs [Internet]. Babraham Institute Blog; 2022 Jan 19. Available from: https://www.babraham.ac.uk/blog/roving-researcher-faq

## A56 Systemic diversity interventions in research intensive settings: a participative approach

### Carolin Ossenkop^1*^, Claartje J Vinkenburg^2^

#### ^1^Connectify, Wychen, Netherlands; ^2^CJ Vinkenburg Advies, Amsterdam, Netherlands

##### **Correspondence:** Carolin Ossenkop (carolin.ossenkop@connectify.nu)

*BMC Proceedings 2023*, **17(15):**A56

In this practice contribution to the 2022 Diversity Interventions conference, we propose design specifications and evidence-based recommendations for systemic diversity interventions in research intensive settings. We base these recommendations on recent commissioned projects in research intensive settings, including research funding organizations (RFOs), and research performing organisations (RPOs, e.g. universities and research institutes). These interventions are or could be used to help develop an EDI strategy, to build a customised action plan for Athena Swan or Race Equality Charter applications, or to meet the European Commission Horizon Europe Gender Equality Plan requirement. In addition, these interventions can used in the evaluation of existing action plans or the redesign of next generation plans.

For our approach we build upon ideas laid out by Vinkenburg [1], who argues that for diversity interventions in upward-mobility career systems to have sustainable impact, it is crucial to engage gatekeepers, to mitigate bias, and to improve decision making. Our work is grounded in several underlying principles: evidence-based; participative; intersectional (i.e. using an intersectional lens); context-specific.

We develop and implement systemic diversity interventions in co-creation with clients (typically diversity officers, taskforces or committees, or senior EDI policy advisors, and by inviting senior decision-makers such as selection committee members and chairs), following an iterative process of several rounds: 1) Diagnosis (i.e. what is it that the organisation wants to achieve? What is the context-specific reality of the organisation?); 2) Inspiration (i.e. what can the organisation learn from relevant others?); 3) Design (i.e. what can the organisation do to achieve their pre-defined goals?); 4) Consolidation and/or evaluation (i.e. what is an adequate and realistic timeline/roadmap? Who is going to be involved/do what? What are adequate ways of measuring and evaluating actions across time?).

In terms of content, we collect evidence and especially critical incidents related to at least two but preferably three subject areas: 1) structural (e.g. domain, hierarchical shape, career system); 2) interactional (e.g. conversations, jokes, micro-aggressions); cultural (e.g. unwritten rules, celebrations, ideal academic).

As our approach is per definition customised, we cannot offer tools or modules that can be simply copied across different contexts. However, inspirational examples of recent interventions are: process optimisation (e.g. funding decisions [2,3]); participant observation (e.g. appointment committees); experiential learning (e.g. including behaviour training [4]); visualisation and ideation (e.g. drawing the ideal career).

**References**

1. Vinkenburg CJ, Ossenkop C, Schiffbaenker H. Selling science: optimizing the research funding evaluation and decision process. Equal Divers Incl. 2022;41(9):1-14.
2. Dutch Research Council [Internet]. Inclusive assessment. Available from: https://www.nwo.nl/en/inclusive-assessment
3. Including Behavior Institute [Internet]. Training. Available from: https://includingbehavior.com/including-behavior-training/
4. Vinkenburg CJ. Engaging gatekeepers, optimizing decision making, and mitigating bias: design specifications for systemic diversity interventions. J Appl Behav Sci. 2017;53(2):212-34.

## A57 The narrative turn: critical reflections on adopting a narrative CV in research assessment

### Claartje J Vinkenburg^1*^, Aleksandra (Ola) Thomson^2^

#### ^1^CJ Vinkenburg Advies, Amsterdam, Netherlands; ^2^Elizabeth Blackwell Institute, University of Bristol, Bristol, United Kingdom

##### **Correspondence:** Claartje J Vinkenburg (c.j.vinkenburg@gmail.com)

*BMC Proceedings 2023*, **17(15):**A57

Research funding organisations (RFOs) are spearheading a major shift in research assessment through the adoption of a narrative CV [1-4]. A narrative CV describes contributions and achievements that reflect a broad range of skills and experiences. This attempt at making assessment more inclusive and recognising individuals’ wider contributions to research ecosystems is arguably a significant diversity intervention requiring attention. Following RFOs’ lead, research performing organisations (RPOs, including higher education) are considering the adoption of narrative CV in recruitment, promotion and possibly development.

While academic CVs had a more narrative nature until the 1980s, recently such formats have been phased out in favour of lists of positions, publications, citations, awards and grants. However, an individual’s overall contributions to research go beyond proxy measures of outputs and impact. This format of listing achievements does not clearly demonstrate the individual’s contributions to research communities, “real world” societal impacts or influence on the field.

A narrative CV allows individuals to craft a coherent description of their contributions, such as teaching, innovation, valorisation, outreach, supervision, administration as well as publications. A narrative account of achievements allows reviewers to clearly see and reward the full range of contributions, experiences and careers necessary to create a positive research culture, to promote diversity and to support inclusive environments that enable excellence. However, this requires evaluators to learn to assess this complexity and expand their traditionally narrow ideals of candidate and career.

Additionally, there is room for bias as narrative CVs rely on language and storytelling. Research on recommendation letters and written performance evaluations show that pronoun use, verbal (un)certainty, labels and negations likely have an effect. Higher standards, extra scrutiny and questioning independence are likely to occur. Although revealing personal context (parental status, health, socioeconomic background) may help to explain trajectories, at the same time this may reproduce and reinforce “lack of fit”.

Therefore, it is crucial that evaluators are trained to assess and compare such CVs if we hope to boost diversity and inclusion through broadening what we value and recognise in research contributions. Designing a carefully considered structure in evaluation processes (especially in the application of criteria) would facilitate bias mitigation, but also prevent putting the onus or the burden of proof on applicants.

**References**

1. Elizabeth Blackwell Institute for Health Research [Internet]. Bristol: University of Bristol. Résumé for Researchers: the whys and hows of narrative CVs; 2021 Sep 15. Available from: http://www.bristol.ac.uk/blackwell/news/2021/narrative-cvs-blog.html.
2. Hamann J, Kaltenbrunner W. Biographical representation, from narrative to list: the evolution of curricula vitae in the humanities, 1950 to 2010. Res Eval. 2022;31(4):438-51.
3. Fritch R, Hatch A, Hazlett H, Vinkenburg C. Using narrative CVs: process optimization and bias mitigation. Zenodo; 2021 Dec 22. 7 p. Supported by Horizon 2020.
4. Vinkenburg CJ, Ossenkop C, Schiffbaenker H. Selling science: optimizing the research funding evaluation and decision process. Equal Divers Incl. 2022;41(9):1-14.

## A58 Faculty-led Dimensions Pilot team as community of practice

### Art M Blake

#### Ryerson University, Toronto, Canada

##### **Correspondence:** Art M Blake (art.blake@ryerson.ca)

*BMC Proceedings 2023*, **17(15):**A58

My major goal as Director of my university’s Dimensions Pilot Program, and designer of its structure, has been to build a team of full-time, tenured faculty members (“academic staff” in UK) in each Faculty to serve as EDIA leaders in their local areas of research practice. Designated “Dimensions Faculty Chairs” (DFCs), they now receive one course release and a research stipend, and hire a postdoctoral fellow, a graduate student and an undergraduate student to work with them, all of whom are paid. Each DFC has developed and led “town hall” events and workshops, and have had one-on-one conversational “interviews” with faculty colleagues about EDIA in research/creative activities. This networked leadership model has proved very successful in building recognition of, and respect for, the Dimensions program and its goals. Our diverse team – me and the 8 DFCs – evolved quickly into a supportive, mutually educational “community of practice,” meeting monthly as well as engaged via email and online documents, generating rich discussions that will be reflected in our Dimensions application and internal reports.

As we move into compiling our application for Dimensions recognition, and writing up an internal-facing critical assessment, I’d like to share my team’s practical experience, successes and challenges, and our plans for securing the model’s future beyond the Pilot’s conclusion in December 2022.

I would also like to address the benefits of the political context of our Dimensions work: an active “truth and reconciliation” process at the university, including changing the university’s name, in response to the “Calls for Action”, a process intended to name and address the colonial legacies of harm done to Indigenous peoples in Canada; in addition, the Black Lives Matter movement has provided vital impetus to the university leadership to analyse and address systemic anti-Black racism at our institution and in Canadian higher education.

## A59 Gender, academic power and citizenship – the impact of international recruitment on gender balance in climate research

### Agnete Vabø^1^, Evanthia K. Schmidt^2^, Cathrine Egeland^1^, Thomas Franssen^3^

#### ^1^Oslo Metropolitan University, Oslo, Norway; ^2^Aarhus University, Aarhus, Denmark; ^3^Leiden University, Leiden, Netherlands

##### **Correspondence:** Agnete Vabø (agnete.vabo@oslomet.no)

*BMC Proceedings 2023*, **17(15):**A59

Research and higher education have been strongly influenced by change processes related to international cooperation and global competition to create world-leading research environments. In Norway, a small country with large public investments in research [1], international collaboration and recruitment to raise the quality of research have been strong driving forces. Today, the majority of qualified applicants for Norwegian professorships comes from institutions abroad, and foreign researchers make up approx. 30 per cent of the total number of professionals in the sector.

Despite the great political interest in internationalisation and recruitment of foreign researchers, there has been limited research on the importance of international recruitment for the research environments [2], the research agendas and the local power structures. Gender equality and internationalisation policy have functioned as two separate fields in research policy. Nonetheless, in its recent internationalisation action plan, the Research Council of Norway states that international mobility ought to safeguard gender balance and consider the gender dimension in research and innovation.

The study presented herewith is carried out in the frame of the project Gender, Academic Power and Citizenship, funded by the Programme on Gender Balance in Senior Positions and Research Management (BALANSE) of the Research Council of Norway [3], aiming at bringing structural and cultural change in the research system. The study explores the impact of internationalisation on the gender balance within climate research. As a result of international recruitment, the proportion of women is rising – but gender is constructed in manifold ways in different academic contexts. Here we look at climate research in the light of a horizontal understanding of globalised research communities.

The presentation is based on quantitative data, policy documents and in-depth interviews with foreign women and men in climate research, where Norway has a renowned research environment.

International recruitment has contributed to an increase in the proportion of women in STEM and at professor level [4]. The case of climate research shows that international recruitment has contributed to a transformation of the field – both in terms of gender composition, distribution of power and resources in a gender perspective, and renewed research agendas [5]. Moreover, the findings point to that international recruitment for gender balance should be analysed from an intersectional perspective, as foreign researchers are not a homogeneous group, and consider the complexity that characterises the field as disciplines have different epistemic, organisational and social characteristics.

**References**

1. Lepori B, Seeber M, Bonaccorsi A. Competition for talent. Country and organizational-level effects in the internationalization of European higher education institutions. Res Policy. 2015;44(3):789-802.
2. Bauder H. The international mobility of academics: a labour market perspective. Int Migr. 2015;53(1):83-96.
3. Research Council of Norway. Programme on Gender Balance in Senior Positions and Research Management (BALANSE), Work programme 2017–2022. Oslo: Research Council of Norway; 2017. 15 p.
4. Pietilä M, Drange I, Silander C, Vabø A. Gender and globalization of academic labor markets: research and teaching staff at Nordic universities. Soc Incl. 2021;9(3):69-80.
5. Research Council of Norway. Handlingsplan for internasjonalisering 2021-2027. Oslo: Research Council of Norway; 2021 May. 14 p.

## A60 Perceptions of gender equity and markers of achievement in a National Institute for Health Research (NIHR) Biomedical Research Centre (BRC): a qualitative study

### Lorna R Henderson^1,2*^, Rinita Dam^3^, Syed Ghulam Sarwar Shah^2,4^, Pavel Ovseiko^2^, Vasiliki Kiparoglou^4,5^

#### ^1^National Institute for Health Research Oxford Biomedical Research Centre, Oxford, United Kingdom; ^2^Radcliffe Department of Medicine, University of Oxford, Oxford, United Kingdom; ^3^Department of Zoology, University of Oxford, Oxford, United Kingdom; ^4^NIHR Oxford Biomedical Research Centre, Oxford, United Kingdom; ^5^Nuffield Department of Primary Care Health Sciences, University of Oxford, Oxford, United Kingdom

##### **Correspondence:** Lorna R Henderson (lorna.henderson@ouh.nhs.uk)

*BMC Proceedings 2023*, **17(15):**A60

For a full report published elsewhere, please see: Henderson LR, Dam R, Shah SGS et al. Perceptions of gender equity and markers of achievement in a National Institute for Health Research Biomedical Research Centre: a qualitative study. Health Res Policy Sys 2022;102(20). https://doi.org/10.1186/s12961-022-00904-4

## A61 Do we need a feminist bibliometrics?

### Claire Donovan

#### University of Greenwich, London, United Kingdom

##### **Correspondence:** Claire Donovan (c.a.donovan@greenwich.ac.uk)

*BMC Proceedings 2023*, **17(15):**A61

**Background**

Bibliometrics is the scientific investigation of the quality or scientific impact of academic publications, based on data about research productivity and citation numbers. Bibliometric data are increasingly used by research managers, research funders and the academic community to assess research excellence, and are assumed to be an objective basis for decisions about hiring, promotion and awarding grants. However, empirical studies reveal that the concept of academic excellence is a social construct, is gendered, and discriminates against women [1,2].

**Methods**

A literature search was conducted using Web of Science from the year 2000 onwards. Search 1 focused on the broader literature about gender and notions of academic excellence; Search 2 focused on specialised studies of gender and bibliometric data.

**Results**

The broader literature reveals systemic gender bias in the production and interpretation of data that shape notions of ‘excellence’. This includes teaching evaluations and data used in academic recruitment, granting promotion/tenure and the awarding of funding. Studies on publication metrics report gender effects regarding research productivity, journal peer review decisions, the gender citation gap and the lower visibility (and hence lower status) of feminist research and gender studies. There is conflicting evidence for and against bibliometrics being a technology that can harm [3] or liberate women academics [4], and which can expand our understanding of the dynamics of gender studies and feminist scholarship within the wider research system [5].

**Conclusions**

There is extensive evidence of gendered data collection and interpretation negatively affecting women’s career progression. Bibliometric data are generally viewed as neutral, yet can amplify gender biases when used to inform judgements about research excellence. Following the example of feminist economics, there is a case for a feminist bibliometrics. Feminist economics is a gender-aware, inclusive approach to economic enquiry, which highlights the social construction of traditional economics and offers alternative methods and models. Feminist bibliometrics is a potential intervention for data suppliers and users, which accepts that bibliometric data are socially constructed, recognises the need for indicator design to be gender-sensitive, and exposes and removes gendered assumptions and biases. The purpose is to provide gender-sensitive data to inform academic recruitment and promotion/tenure decisions, and ‘track record’ data for grant review panels; and to refine our understanding of what constitutes ‘excellence’ in research.

**References**

1. Rees T. The gendered construction of scientific excellence. Interdiscip Sci Rev. 2011;36(2):133-45.
2. Van den Brink M, Benschop Y. Gender practices in the construction of academic excellence: sheep with five legs. Organization (Lond). 2012;19(4):507-24.
3. Brooks C, Fenton EM, Walker JT. Gender and the evaluation of research. Res Policy. 2014;43(6):990-1001.
4. Van Arensbergen P, Van der Weijden I, Van den Besselaar P. Gender differences in scientific productivity: a persisting phenomenon?. Scientometrics. 2012;93(3):857-68.
5. Pearse R, Hitchcock JN, Keane H. Gender, inter/disciplinarity and marginality in the social sciences and humanities: a comparison of six disciplines. Womens Stud Int Forum. 2019;72:109-26.

## A62 Implementing an Athena Swan action plan – reflections on successes and challenges

### Dianne L Cantali

#### University of Dundee, Dundee, United Kingdom

##### **Correspondence:** Dianne L Cantali (dlcantali@dundee.ac.uk)

*BMC Proceedings 2023*, **17(15):**A62

**Background**

This reflective presentation considers the implementation of the School of Education and Social Work’s Athena Swan action plan, which is in its final 6 months. This was written to accompany the School’s successful application for an Athena Swan Bronze award. This reflective presentation forms part of the process of evaluating the progress made since the Bronze award was gained, and planning for making an application to renew the award in the 2022–23 academic session.

**Methods**

Overall, the implementation of the action plan has been overseen by the School Equality, Diversity & Inclusion committee which meets four times per year. An annual report has been produced which summarises progress and identifies priorities for the coming year. Actions have been progressed by sub-groups and individuals, leading on actions which align with their interests and professional roles. Some actions were implemented by relevant School committees. The action plan has been mapped against relevant University action plans, for example the Race Equality Charter action plan which accompanied the successful University application for a Bronze award, the University’s Athena Swan action plan.

**Results**

The successes that have arisen from implementing the action plan, as well as the challenges that we have encountered, will be discussed. Successes have included the development of women’s career development workshops, held jointly with the Schools of Humanities and Social Sciences, and the Athena Swan staff and student surveys, both of which have been run twice. Comparator data shows a welcome increase in positive responses over the two years between surveys. Another success has been the introduction of a seminar series celebrating gender equality, this being jointly with the School of Business. The benefits of working collaboratively with EDI and Athena Swan lead colleagues from across the University, and how these relationships have developed, will be discussed. Inevitably, there has been a detrimental impact on the timing and capacity of colleagues to implement some aspects due to the COVID-19 pandemic. The most significant of these was the delay of over 2 years to the holding of the inaugural Mapstone lecture, celebrating the University’s first female professor who held a Chair in Social Work. Other challenges, including limited response rates to surveys due to ‘survey fatigue’, and how we might engage with staff and students differently in the future, will be discussed.

**Conclusions**

Overall, there have been sustained positive actions to support colleagues with a range of protected characteristics within the School. While the key focus was on developing opportunities for women, many of the actions in the Action Plan served to benefit others. We are in a strong position to move forward with an application for renewal under the transformed Athena Swan charter, which will have an increased focus on intersectionality and the culture within the School.

## A63 Gender equality work in a distance learning institution

### Nicole Lotz, Advaith Siddharthan, Cinzia Priola, Janette Wallace

#### The Open University, Milton Keynes, United Kingdom

##### **Correspondence:** Nicole Lotz (nicole.lotz@open.ac.uk)

*BMC Proceedings 2023*, **17(15):**A63

With many HE institutions increasingly adopting hybrid working models and expanding their online education portfolio, an examination of the barriers as well as best practice for developing and implementing equality work in distance education settings is timely.

The Open University (OU) is the largest university in the UK, delivering flexible, distance education across the UK, Ireland and internationally. The OU holds an institutional Athena Swan Bronze Award.

Most of the Science, Technology, Engineering and Mathematics (STEM) departments hold awards at Silver level. Arts, Humanities, Social Sciences, Business and Law (AHSSBL) departments are running their initial self-assessment processes, with the Business School being recently awarded a Bronze Award. Looking across the self-assessments in these departments, the OU’s Athena Swan Champions Network – a group of the Chairs of self-assessment teams (SATs) in the departments – has identified specific gender equality challenges and opportunities that arise in a distance education setting.

**Challenges**

A large, distributed organisation, in both regions and nations, produces substantial amounts of data, and introduces errors and inconsistencies in data. Several actions set out to develop a comprehensive system for data collection and monitoring.

The distributed nature of our institution also made our equality efforts less visible. The OU has no undergraduate students on campus, and many staff are homeworkers. Several actions focused on establishing regular communication channels (and core working hours policies for meetings), as well as events to increase visibility, both online and face-to-face, such as a Women in Engineering student conference, or Maven of the Month continuing the work on the BBC internet talk radio show in 1995, and celebrations of diverse role models for Ada Lovelace Day, International Women’s Day, LGBTQ+ week, etc. While our partnerships with the BBC and open educational resources on FutureLearn or OpenLearn reach millions, monitoring their uptake is a challenge.

Further actions seek to tackle workload management in a distributed environment which can obscure transparency of workload allocation. The monitoring and reporting of workload allocation is often described as a ‘work of fiction’. While this offers freedom and independence to some staff, it leads to reports of dissatisfaction by other staff.

**Opportunities**

Actions across departments on making interview panels more gender balanced, ensuring gender balanced imagery in prospectuses, and the increasing visibility of our equality work, had positive impact on diversifying the recruitment of staff and postgraduate research students. While flexible working attracts more women to the OU, it is our responsibility to make sure their careers develop as well. Online support of career development, through training, leadership courses, peer mentoring and working groups, improving gender balance in staffing boards and making equality and inclusion work a career pathway had positive impact across the institution, with all the Athena Silver departments achieving gender balance in promotions. The increasing research and scholarship that focuses on inclusion had a positive wider impact, attracting more staff and students who are strengthening this work.

In conclusion, hybrid working brings the greatest challenges to our equality work. COVID-19 induced home working across the board brought unexpected opportunities for inclusion to the fore.

## A64 MDS SUSTAIN: a novel leadership programme at the University of Birmingham to address the leaky pipeline for women and other demographics which are underrepresented in senior academic positions

### Tom M Syder^1*^, Laura Meagher^2^, Vincent Cornelius^1^, Sarah Conner^1^, David Round^1^, Gaynor Miller^1^, Wiebke Arlt^1^

#### ^1^University of Birmingham, Birmingham, United Kingdom; ^2^Technology Development Group, Dairsie, United Kingdom

##### **Correspondence:** Tom M Syder (tomsyder@hotmail.co.uk)

*BMC Proceedings 2023*, **17(15):**A64

MDS SUSTAIN is a pilot leadership programme which supports the development of researchers establishing their first independent research group. Named after the University of Birmingham College of Medical and Dental Sciences (MDS) and the Academy of Medical Sciences’ SUSTAIN leadership programme, MDS SUSTAIN began its inaugural year last September.

While the national SUSTAIN programme is restricted to female researchers, this version of the programme was expanded to all groups which are underrepresented in senior academic positions (including but not limited to ethnic minority, LGBTQ+, disabled and first-generation academics). This decision was taken to extend the benefits of the programme, increase value for money by widening the pool of eligible participants, and avoid a deficit model. The latter point was achieved by focusing the application process on the benefits which participants expected to gain, inviting them to write reflectively about, for example, experiences of imposter syndrome or low self-confidence. This made the programme accessible and relevant to staff from the aforementioned demographics without being restricted to them or requiring their applications to focus on their identities.

The pilot programme received 21 applications, with places offered to 17 primarily based on the remaining applicants having progressed far enough to be eligible for more senior leadership programmes. Two-thirds of the inaugural cohort are women, which exceeds the proportion of eligible women and vindicates the approach taken to promote the programme to staff from underrepresented groups. This also lays the groundwork for the faculty’s overarching objective of mitigating the leaky pipeline by better supporting female staff at the start of their careers.

The programme consists of mentoring (delivered by graduates of the national SUSTAIN programme), peer coaching, and a series of training sessions, largely based on those identified by the Academy of Medical Sciences as being relevant to staff at this career stage. The programme has capitalised on its unique element of participant diversity by including additional training sessions about authentic leadership; these are intended to emphasise the importance of participants bringing their whole selves to work while enabling them to learn from each other about the impact of their diverse identities on their workplace experiences.

The programme will shortly open to applications for a second cohort, with the first year being evaluated (with support from evaluation specialist Laura Meagher) to determine effectiveness. While the long-term goal is to diversify senior academic positions, short-term evaluation measures focus on the perceived benefits to participants. To date, two surveys and a set of focus groups have taken place, with preliminary results indicating that the programme has been effective in providing space away from daily pressures to reflect, as well as increasing the skills, professional confidence, and leadership aspirations of participants. The mentoring and cohort aspects of the programme are rated particularly highly. An area for improvement is mode of delivery (which was exclusively online during the pandemic); in future the programme will be hybrid, keeping many of the flexibility and sustainability benefits of the current model while providing more opportunities for in-person community building.

## A65 Diversity in Research Group’s development of a demographic survey for patient and public involvement contributors

### Rachel Taylor^1,2*^, Alexandra Almeida^2,3^

#### ^1^Oxford University Hospitals NHS Foundation Trust, Oxford, United Kingdom; ^2^University of Oxford, Oxford, United Kingdom; ^3^Oxford Health NHS Foundation Trust, Oxford, United Kingdom

##### **Correspondence:** Rachel Taylor (rachel.taylor@ouh.nhs.uk)

*BMC Proceedings 2023*, **17(15):**A65

The Oxford Health and Oxford Biomedical Research Centres (BRCs) have developed a survey to better understand the demographic make-up of members of their various patient and public involvement and engagement (PPIE) groups.

Ensuring that everyone, regardless of their background, has the opportunity to be involved in research is a key priority for both Oxford BRCs. The survey helps us identify which communities are least involved in research so we can develop outreach programmes to facilitate their inclusion in research.

The ‘Tell Us About You’ survey was developed with vital input from the two BRCs’ Diversity in Research Group [1], a PPIE Advisory group with 15 members, mainly from minority ethnic communities and other groups who are not usually involved with research, such as younger adults, carers, and people from LGBT+ communities. The Diversity in Research Group wanted to help with developing the survey because they pointed out that demographic survey questions often feel tokenistic and not inclusive.

This survey, available for use across the BRCs’ PPIE groups, captures demographic information such as age, gender and race to get a clearer picture of who is involved in research, how representative they are of the general population and of the sections of society that are underrepresented.

The first stage of the improvement process involved a group discussion about the content of an existing demographic survey. We made amendments following this input. We then developed questions on each part of the survey to systematically collect the views of members of the group. The following improvements were made to the survey:

1. Changing the name of the survey from “Demographic survey” to “Tell us about you”.
2. Making the purpose of the survey clear – why is this information being collected and how will it be used?
3. Putting the sexuality and ethnicity categories in alphabetical order, rather than having “heterosexual” and “white” first.
4. Removing the word “highest” from the question “what is your highest level of education?”; and adding an option for overseas qualifications.

Using the data collected by the survey and the underrepresented groups identified in the NIHR INCLUDE project [2], the Diversity in Research Group members are now identifying communities that are not represented in the group, such as asylum seekers and refugees, and people who are transgender.

**References**

1. NIHR Oxford Biomedical Research Centre [Internet]. Oxford: NIHR Oxford Biomedical Research Centre. Diversity in Research Group. Available from: https://oxfordbrc.nihr.ac.uk/ppi/diversity-in-research-group/
2. NIHR: National Institute for Health and Care Research [Internet]. National Institute for Health and Care Research. Improving inclusion of under-served groups in clinical research: guidance from INCLUDE project; 2020 Aug 07. Available from: www.nihr.ac.uk/documents/improving-inclusion-of-under-served-groups-in-clinical-research-guidance-from-include-project/25435

## A66 Sharing Athena Swan good practice across STEM disciplines at The Open University

### Advaith Siddharthan, Nicole Lotz, Gwyneth Stallard, Victoria Pearson, Clem Herman, Magnus Ramage, Andrew Potter, Miriam Fernandez

#### The Open University, Milton Keynes, United Kingdom

##### **Correspondence:** Advaith Siddharthan (advaith.siddharthan@open.ac.uk)

*BMC Proceedings 2023*, **17(15):**A66

The Open University’s (OU) Science, Technology, Engineering and Mathematics (STEM) faculty has been its earliest adopter of the Athena Swan Charter. All seven STEM departments have Athena Swan awards; four have achieved Silver and a fifth recently submitted its Silver application. All are part of an Athena Swan Champions Network that shares ideas and good practice, leading to some similarities in actions and outcomes. In this abstract we summarise departmental Bronze actions and the impacts they have had, as evidenced in five departmental Silver applications across mathematics, the physical sciences, engineering and computing. We only report on actions that impact central academic staff for reasons of space.

*Recruitment:* Across departments, effort has gone into (a) organising departmental training on unconscious bias to supplement the OU’s online course, (b) ensuring diversity on selection panels, (c) modifying websites to increase visibility of women and showcase commitments to gender equality, (d) ensuring inclusive language use in advertisements, and (e) making use of women in maths/science/computing/engineering mailing lists and networks. Consequently, recruitment has become fairer, with three departments reporting gender balanced recruitment overall, and all reporting gender balanced recruitment at grades Lecturer and above, with proportions of recruited female postdoctoral researchers above national averages. Such efforts have also improved the gender balance of our PhD cohorts, with all departments now reporting proportions of women well above national averages.

*Promotions:* Departments have moved to (a) ensuring appraisal of all staff annually, with career progression a key topic of discussion, (b) offering mentoring for promotion applications, (c) postdoctoral reviews that encourage women to apply for promotions, (d) providing training for staff to understand promotions criteria, (e) gender balancing staffing/promotions panels, (f) encouraging leadership programmes like Aurora, (g) monitoring gender in leadership and committee positions, and (h) moving towards greater shared leadership, including creation of deputy roles. Through these actions, departments have achieved fairer promotions, minimally proportional to staff gender ratios, and in one case gender balanced. Through fairer recruitment and promotions, the proportion of women has increased across all departments (by 5 to 10 percentage points) and the proportion of women professors are now substantially above national averages for four departments (ranging from 29% to 60% of all professors)

*Maternity:* All departments have taken steps to (a) make staff aware of OU policies, (b) establish schemes for a maternity buddy and Keep in Touch days, (c) promote flexible working, and (d) increase entitlements for returners, from conference costs, to carrying over leave, to reduced teaching loads. All departments also follow a core working hours policy of 10 am to 4 pm for meetings, seminars and social events. Across the five departments, just a single woman (professional staff) did not return following maternity, and just one further woman (research associate on an external grant) left within 18 months of returning. All contracts that expired during maternity leave were renewed.

To conclude, the OU’s STEM faculty has benefited substantially from sharing ideas for gender equity with similar actions leading to similar impacts across departments between Athena Swan Bronze and Silver submissions.

## A67 The relationship between micro-behaviours and impostor syndrome

### Gillian Arnold

#### Tectre Ltd, Huddersfield, United Kingdom

##### **Correspondence:** Gillian Arnold (info@tectre.com)

*BMC Proceedings 2023*, **17(15):**A67

In a recent survey conducted by Tectre Ltd [1], the majority of respondents suggested that a good understanding of micro-behaviours, and their impacts, were important to help them have a perspective on the culture within the working environment. In a separate survey question, the majority of respondents also indicated that their own personal lack of self-confidence was the key inhibitor to their progression. Tectre Ltd are clear that there is a direct relationship between the workplace where there is a tendency for significant numbers of micro-behaviours and the self-doubt of those in minority groups.

It is recognised that within internal institutional culture both in academia and in industry, hierarchies of privilege ensure that people in minority groups encounter significantly more damaging micro-behaviours than those in higher tiers of the hierarchy of privilege. It is also true that those who are in the more privileged positions in the organisation, or within society, are challenged to recognise that micro-behaviours exist, or to recognise their own part in delivering them. Where individuals in the minority groups repeatedly encounter microaggressions and micro-inequities, they are subject to a build-up of self-doubt, which is continually reinforced by further micro-behaviours leading to stress, anxiety, stereotype threat and the inability to reach one's full potential. For this reason, our proposal for best practice is to run workshops that help individuals understand the impact of micro-behaviours on their sense of self-worth, the link with confidence issues such as impostor syndrome and how to arrest the cycle driving this.

Broadly, the workshops explore micro-behaviours and personal privilege, and would include information on how to mitigate the effects on self-belief. To have an impact at all, the workshops must be fully interactive: with exercises that explore known micro-behaviours and offer participants a chance to recall instances where micro-behaviours have had an impact on their beliefs about themselves. They should include scenario-based explorations of the different types of micro-behaviours and would offer specific examples and what each example is really saying to the individual recipient. It would also usefully help participants to place themselves within societal hierarchies and understand how likely they were to experience greater or fewer numbers of micro-behaviours aimed at them.

The workshops then turn to mitigating strategies around the impostor syndrome, being clear not to pathologise the participant. The workshops explore the ways in which change can be brought within the institutions to ensure that there is a general awareness of the damage that micro-behaviours can have on the cultural feel of the workplace, and how lack of self-worth within the staff and student cohorts can limit the potential of all. For more information about these and other workshops, contact Tectre Ltd [https://tectre.com/].

**Reference**

1. Tectre Survey [Internet]. Available from: https://docs.google.com/forms/d/e/1FAIpQLSfgQuP_b8XbBzDEd1n62GD4zKruhBZVXQ6JkdLcN1WRSpVlUQ/viewform

## A68 #SayIt – a student-led initiative shedding light on everyday discrimination to fight structural violence at the medical faculty and hospital

### Leonie Mac Fehr, Jacqueline Niewolik, Merve Kocyigit, Annemarie Fanny Müller, Hannah Marie Heseding, Annika Marie Tödtmann, Anna Maria Kastner

#### Medical School Hannover, Hannover, Germany

##### **Correspondence:** Anna Maria Kastner (kastner.anna@mh-hannover.de)

*BMC Proceedings 2023*, **17(15):**A68

**Background**

Discrimination in the workplace is a widespread global issue that can affect anyone [1]. It comes in different forms and can negatively impact individual outcomes [2]. Especially the university hospital setting is of special interest because discrimination not only occurs between employees but also between patients, doctors, staff and students. Yet there have been few efforts to prevent discrimination in the medical field [1]. In 2019, students of Hanover Medical School, Germany (MHH) have launched the anti-discrimination initiative #SayIt. #SayIt aims to increase awareness by anonymously publishing reports of everyday discrimination at MHH and empowering staff and students to take action through workshops.

**Methods**

#SayIt uses the online questionnaire program SoSci Survey for admission. All persons associated with MHH can hand in reports of situations in which they experienced discrimination in the university or hospital context. If permission is given by the reporting person, the submission will be anonymously published on the university’s website [https://www.mhh.de/sayit], posters on campus, and via Instagram. The submissions confirmed the urgency and relevance to act and led to the development of an empowerment workshop that applies an intersectional feminist framework. During the peer-to-peer workshop, participants can share experiences and develop and practise strategies for future situations.

**Results**

To date, we have received 59 submissions. On our Instagram account (say_it_hannover), we regularly share submissions and provide educational background information with our 549 followers. A national exchange has developed and further #SayIt groups have been established at other medical faculties. The workshop was conducted three times and is to be implemented as a regular monthly seminar affiliated with the university's own skills lab. So far our initiative has been sustained by unpaid activist work. To achieve sustainable change, #SayIt and the gender equality office submitted a funding application for an anti-discrimination counsellor, which was rejected by the university.

**Conclusions**

Since 2019, #SayIt has shed light on discrimination in the hospital as a workplace, university and research institution. It continues to provide tools to actively confront and fight discrimination. Due to the structural component of all forms of discrimination, the request for institutional support has been and remains one of the core issues of the #SayIt initiative. While our initial focus was on raising awareness of discrimination, building and nurturing a critical and supportive community has become increasingly important given the lack of action from the university’s side. Together we share experiences, educate and empower each other or as bell hooks wrote, “For one of the most vital ways we sustain ourselves is by building communities of resistance, places where we know we are not alone” [3].

**References**

1. Jenner SC, Djermester P, Oertelt-Prigione S. Prevention strategies for sexual harassment in academic medicine: a qualitative study. J Interpers Violence. 2022;37(5-6):NP2490-NP2515.
2. Dhanani LY, Beus JM, Joseph DL. Workplace discrimination: a meta-analytic extension, critique, and future research agenda. Pers Psychol. 2018;71:147-79.
3. hooks b. Yearning: race, gender, and cultural politics. Boston, MA: South End Press; 1990. 236 p.

## A69 Women and spinouts: a case for action

### Simonetta Manfredi

#### Oxford Brookes University, Oxford, United Kingdom

##### **Correspondence:** Simonetta Manfredi (smanfredi@brookes.ac.uk)

*BMC Proceedings 2023*, **17(15):**A69

This session provides an overview of the outputs and interventions developed as part of a project on *Women and Spinouts: A Case for Action*, funded by the Engineering and Physical Sciences Research Council as part of their *Inclusion Matters* programme (2018–2021) [1]. This project was led by the Centre for Diversity Policy Research and Practice (CDPRP) in collaboration with the Mathematical, Physical and Life Sciences Division at the University of Oxford. It followed from a discussion paper published by the CDPRP, highlighting the underrepresentation of women researchers in the founding and governance of university spinout companies and the need to address this gender imbalance [2].

Key project objectives included: 1) map women’s participation as founders in university spinout companies; 2) understand of how gender affects the experience of women scientists involved in setting up spinout companies; 3) raise awareness about the importance of embedding a gender perspective in research and innovation in the pursuit of academic excellence; 4) build institutional capacity through the development of resources to tackle cultural and structural barriers.

**The context**

The mapping of women’s participation as founders in university spinout companies in the UK from a gender perspective shows that: only 18% (figure updated to 2022) of active academic spinouts have at least one woman founder and that women founders are less likely to receive large innovation grants and to be featured in high-growth list. A series of interviews with women and men founders about their spinout journey [3] highlighted: shared challenges (both women and men founders identified three key challenges: lack of business experience, the need for universities to properly support spinout-related activities and commercialisation of research through allocation of time, and the need to reward these activities in the academic promotion process); gender bias in the investment community; gender stereotypes and sexism. These findings were complemented by insights from 12 focus groups undertaken in a range of universities with 63 early career researchers, who represent the future pipeline of academic entrepreneurs.

**Developing interventions**

The practice session will focus on sharing how the insights from the above research informed the development of a toolkit co-created with experts from the sector and industry. These tools are aimed at raising awareness about the need to increase women’s participation in spinout leadership and to build institutional capacity to achieve this. They include a framework to develop gender inclusive academic entrepreneurship, speed-mentoring with women founders, videos and case studies. The use of these tools as well as other interventions such as roundtable discussions with key stakeholders will be explored to support women researchers careers in the innovation ecosystem.

All project reports and resources can be freely accessed online [https://www.brookes.ac.uk/women-and-spinouts/].

**References**

1. Griffiths H, Humbert AL. Gender and university spinouts in the UK: geography, governance and growth. Oxford: Oxford Brookes University Centre for Diversity Policy Research and Practice; 2019. 48 p. Supported by the Engineering and Physical Sciences Research Council.
2. Jarboe N, Grisoni L, Manfredi S. University spinouts: exploring women’s participation. A discussion paper. Oxford: Oxford Brookes University Centre for Diversity Policy Research and Practice; 2018.
3. Griffiths H, Grisoni L, Manfredi S, Still A, Tzanakou C. The spinout journey: barriers and enablers to gender inclusive innovation. Summary report. Oxford: Oxford Brookes University Centre for Diversity Policy Research and Practice; 2020. 24 p. Supported by the Engineering and Physical Sciences Research Council.

## A70 Monitoring and evaluation in the professional development of EDI practitioners

### Alvin Leung

#### University of Oxford, Oxford, United Kingdom

##### Correspondence: Alvin Leung (alvinleung@cantab.net)

*BMC Proceedings 2023*, **17(15):**A70

Monitoring and evaluation (M&E) are fundamental functions in the international development sector; they are however less known – although they are already being carried out to some extent – in advancing equality, diversity and inclusion (EDI) in higher education.

Adapting the definitions provided by the Independent Evaluation Group (IEG) of the World Bank [1] to the EDI context, monitoring can be defined as a continuing function that uses systematic collection of data on specified indicators to provide management and the main stakeholders of EDI interventions with indications of the extent of progress and achievement of objectives.

Evaluation, on the other hand, can be defined as the process of determining the worth or significance of EDI activities or programmes to determine the relevance of objectives, the efficacy of design and implementation, the efficiency of resource use, and the sustainability of results. An evaluation should also enable the incorporation of lessons learned into decision-making processes.

I would like to suggest three reasons why knowledge and skills of M&E are important elements in developing the careers of EDI practitioners in higher education.

First, M&E help ensure that EDI activities are data-driven and evidence-based. With M&E data and evidence, EDI practitioners can justify and account for resources and investment, as well as more confidently inform and influence decision makers in universities. The findings from M&E can also help universities share with other institute’s their experiences in advancing EDI to benefit an even larger number of people. In addition, it can be argued that the Athena Swan charter has M&E principles embedded and therefore requires EDI practitioners to understand M&E; three notable examples are success measures, rationales and impact, all of which are key concepts of M&E.

Second, universities are ideal environments for EDI practitioners to develop M&E capabilities. The knowledge and skills of M&E are not dissimilar to those of social science research; for example, both require defining problems/issues and objectives, designing data collection tools and analysing data. Given the similarities and the fact that most universities are staffed with experienced social scientists, universities are well resourced to provide training for their EDI practitioners.

Third, skills involved in conducting M&E are transferrable and can be used to pursue careers in multiple sectors. These transferrable skills include project management, data management, data analysis, communication and strategic planning.

My project in the University of Oxford has enabled me (as an M&E specialist) to co-implement pilot initiatives with EDI practitioners as a meaningful way to share knowledge and skills of M&E. There are also ready-to-use guides on M&E available online; for example, the National Evaluation Guide for STEM equity programs [2] published by Women in STEM Ambassador in Australia is an excellent resource.

**References**

1. Independent Evaluation Group, World Bank Group [Internet]. Washington DC: Independent Evaluation Group. What monitoring and evaluation are. Available from: https://ieg.worldbankgroup.org/what-monitoring-and-evaluation
2. Kingsley I. Evaluating STEM equity programs: a guide to effective program evaluation. Sydney: Office of the Women in STEM Ambassador; 2020. 68 p.

## A71 An analysis of the self-reported impact of COVID-19 on the lifestyle and working arrangements of staff and postgraduate students in a research-intensive department

### Melissa D'Ascenzio, David Booth, Gopal Sapkota, Lesley-Anne Pearson

#### University of Dundee, Dundee, United Kingdom

##### **Correspondence:** Melissa D'Ascenzio (m.dascenzio@dundee.ac.uk)

*BMC Proceedings 2023*, **17(15):**A71

Concerned by reports on the gendered impact of COVID-19 on academic and research staff [1,2], we developed a survey that allowed us to capture the perceived effect of lockdown and other COVID-related restrictions on the lifestyle and working arrangements of staff and postgraduate students in the School of Life Sciences at the University of Dundee.

The survey comprised two parts: in part 1 responders were asked to evaluate the time spent on activities carefully chosen to represent household management, caring responsibilities, home schooling and paid work [3]. In part 2, responders were asked to answer questions about gender, age, ethnicity and disability. The analysis of the behaviour and association of questions suggested that there were two principal sociometric dimensions within this survey questionnaire: one associated with changes in lifestyle and the other with work-related issues. In general, the reliability of responses was acceptable (alpha score > 0.6). While all responses were considered in the qualitative analysis, staff data was included in the quantitative analysis only if they completed part 2 of the survey (240 respondents).

The aggregate scores from responders’ answers were then fed into a model that allowed us to investigate whether protected characteristics were a predictor of the level of disruption that individuals experienced. As a point of reference, a female academic in the youngest category (25 to 29), not disabled, not furloughed, with no caring responsibilities was chosen as benchmark and all other characteristics such as sex, age, disability and role measured against it.

Unsurprisingly, aggregate scores seemed to suggest that most survey respondents experienced significant negative disruption to both their daily life and their work due to COVID-19. Although the data might be subject to confounding, recollection and selection biases, we identified several trends:

1. Staff with caring responsibility reported that the pandemic had the largest impact on their lifestyle, regardless of gender.
2. Early career academics and, more generally, staff in their late 30s to early 40s reported greater changes in their lifestyle, regardless of gender. This observed effect might be driven by caring responsibilities.
3. Postgraduate researchers, academics and research staff showed the highest levels of anxiety around their productivity and job performance.
4. Disabled staff reported a much more negative perception of the impact of the pandemic on their job productivity, performance and flexibility.

**References**

1. Viglione G. Are women publishing less during the pandemic? Here’s what the data say. Nature. 2020 May;581(7809):365-6.
2. Staniscuaski F, Reichert F, Werneck F, de Oliveira L, Mello-Carpes P, Soletti RC, Infanger Almeida C, Zandona E, Ricachenevsky FK, Neuann A, Schwartz IVD, Tamajusuku ASK, Seixas A, Kmetzsch L. Impact of COVID-19 on academic mothers. Science. 2020;368(6492):724.
3. Women Count Team, UN Women. Rapid gender assessment surveys on the impacts of COVID-19: guidance document. New York: UN Women; 2020 May. 30 p.

## A72 Workshop for auditing and improving gender sensitivity in higher education curriculum and teaching

### Tamsin Hinton-Smith

#### University of Sussex, University of Sussex, United Kingdom

##### **Correspondence:** Tamsin Hinton-Smith (j.t.hinton-smith@sussex.ac.uk)

*BMC Proceedings 2023*, **17(15):**A72

This workshop has been developed as an outcome of a collaborative international research project: Gender on the Higher Education Learning Agenda Internationally: Co-constructing foundations for equitable futures (GOTHELAI), a two-year project funded by the British through the Global Challenges Research Fund (GCRF). The intention of the funding stream is to foster international, interdisciplinary collaboration to develop problem-orientated research focused on delivering development impact; fostering equitable research partnerships; and enhancing understanding and responses to global challenges, including in education [1].

Research by the Association of Commonwealth Universities and British Council identifies the effectiveness of international higher education partnerships in supporting the Sustainable Development Goals (SDGs) [2]. This research contributes across SDGs, particularly Education (SDG4), Gender equality (SDG5), Reduced inequality (SDG10) and the importance of these to goals such as Decent work and economic growth (SDG8) and Industry, innovation and infrastructure (SDG9). The research recognises the centrality of higher education teaching and learning in shaping generations of graduates and leaders who will tackle these challenges in their professional lives. This signposts the relevance of this research to the Athena Swan agenda and wider international imperative to increase gender equality in higher education by removing barriers and advancing the careers of women. Increasing numbers, recognition and progression opportunities of women academics across disciplines and countries requires women and men students to see positive representations of gender in the teaching that they receive [3], in order to inspire women to aspire to higher education careers; and men and women to fight together for gender equality within these.

Our research undertaken through surveys and interviews with university staff and students across five international case studies identifies the importance of substantive focus and approach of higher education teaching across disciplines from natural sciences to liberal arts; the expert perspectives and case study examples presented by higher education teachers; and how students of different genders are engaged and values in the higher education classroom. For many classrooms, disciplines and institutions, this imperative requires a radical review and rethink around what is taught, by whom, how, why, and to what effect [4]. This research has been a collaboration of research teams at universities with different profiles in India, Kazakhstan, Morocco, Nigeria and the UK. The international research team spans expertise from Chemistry and Climate change through to Writing Studies, unified by commitment as feminist academics engaged in gender and wider equalities work within higher education at our institutions, including equality, diversity and inclusion leads. The research has led to academic and non-academic outputs tailored to different audiences and including a book in progress, series of reports [4], international online conference, a toolkit and series of workshops to support higher staff engaged in gender equality initiatives to lead gender equality audits of teaching contexts within their own institutions. This session will share this workshop, giving participants an opportunity to engage with considering the relevance of higher education teaching and learning to increasing gender equality in the professional contexts in which they are engaged.

**References**

1. UK Research and Innovation [Internet]. London: UKRI. Global Challenges Research Fund; 2023 Mar 24. Available from: https://www.ukri.org/what-we-offer/international-funding/global-challenges-research-fund/
2. Krcal A, Allinson R, Sutinen L (Technopolis). Role of international higher education partnerships in contributing to the sustainable development goals. British Council and the Association of Commonwealth Universities; 2021 July. 81 p.
3. Morris C, Hinton-Smith T, Marvell R, Brayson K. Gender back on the agenda in higher education: perspectives of academic staff in a contemporary UK case study. J Gend Stud. 2022;31(1):101-13.
4. Hinton-Smith T, Marvell R, Morris C, Brayson K. ‘It’s not something that we think about with regard to curriculum.’ Exploring gender and equality awareness in higher education curriculum and pedagogy. Gend Educ. 2022;34(5):495-511.

## A73 Synergising resources to improve approaches to intersectionality at the University of Dundee

### George Simmonds

#### University of Dundee, University of Dundee, United Kingdom

##### **Correspondence:** George Simmonds (gsimmonds001@dundee.ac.uk)

*BMC Proceedings 2023*, **17(15):**A73

The focus on intersectionality within Equality, Diversity and Inclusion (EDI) initiatives has never been greater and there is an increasing requirement by external charter awards to consider and reflect upon the diversity of people who comprise protected characteristic groups through intersectional approaches.

The University of Dundee currently holds Bronze-level awards in both Athena Swan [1] and the Race Equality Charter [2]. It also holds a Bronze-level award for Stonewall’s Workplace Equality Index [3] and is actively pursuing an award from the Emily Test Charter [4] (which is concerned with preventing gender-based violence). From an internal perspective, having different teams working on separate awards, there is the potential risk that workstreams will become siloed with resources being ineffectively utilised. This could result in intersectional concerns being relegated as a priority for action.

To ensure that intersectional issues are properly considered, the University of Dundee’s approach is to provide an EDI Officer with specific responsibility for external charters. This person attends meetings of different charter teams and staff network groups that specifically allows for this individual to act as a conduit between the groups and highlight where different actions and initiatives can complement each other or where there is duplication of effort (Figure 1). Through the discussion of activities being undertaken by other groups, awareness of intersectional concerns is produced and maintained throughout the different strands of EDI work within the university.

A specific example of this approach can be highlighted through the collaboration of the institutional Athena Swan Self-Assessment Team (SAT) and the LGBT+ Staff Network. The Athena Swan charter requires universities to commit themselves to tackling discrimination against members of the trans community. The EDI Officer recognised that activities which support staff and students who are trans are already being undertaken through our Stonewall Workplace Equality Index Action Plan. It was further recognised that the LGBT+ Staff Network is in a better position to communicate with our trans staff and to understand their specific needs. An invitation was given to one of the Co-Chairs of the LGBT+ Staff Network to become a member of the Athena Swan SAT, which enabled the combined resources of both groups to support the intersection needs of our trans communities.

Similarly, members of the Athena Swan SAT were consulted with in relation to our recent Race Equality Charter Action Plan. This allowed for synergies to be identified where specific interventions will support women from minority ethnic backgrounds, such as improved mentoring, particularly for the academic promotions process.

To conclude, the University of Dundee envisage that there will be a greater need for intersectional approaches to EDI. The key to success in this endeavour will be through greater communication between different workstreams in order to support and strengthen our work in this area.

**References**

1. Advance HE [Internet]. York: Advance HE. The transformed UK Athena Swan Charter. Available from: https://www.advance-he.ac.uk/equality-charters/transformed-uk-athena-swan-charter
2. Advance HE [Internet]. York: Advance HE. Race Equality Charter. Available from: https://www.advance-he.ac.uk/equality-charters/race-equality-charter
3. Stonewall [Internet]. UK Workplace Equality Index. Available from: https://www.stonewall.org.uk/creating-inclusive-workplaces/workplace-equality-indices/uk-workplace-equality-index
4. EmilyTest: tackling gender based violence in education [Internet]. GBV Charter. Available from: http://emilytest.co.uk/charter/

**Fig. 1:**
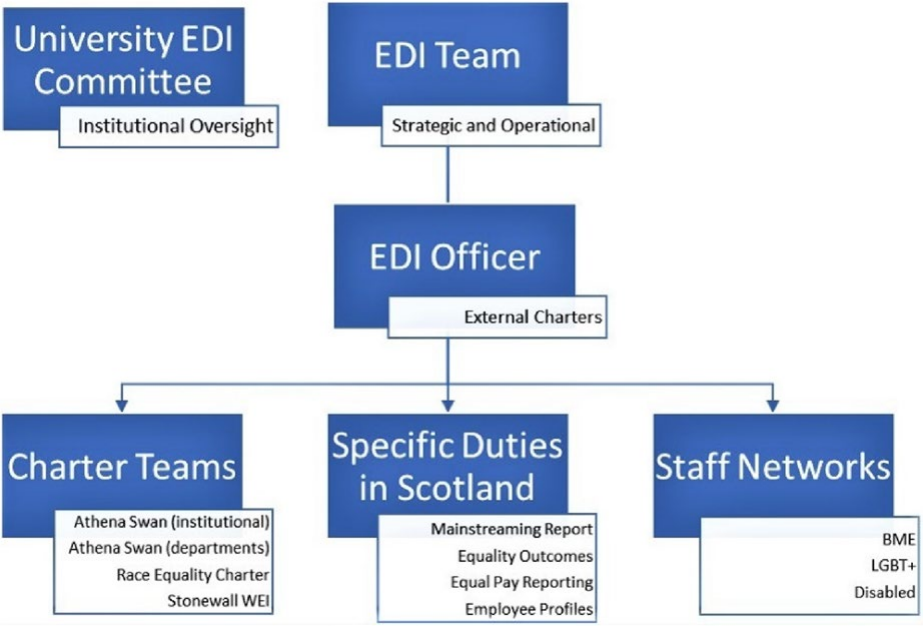
**(abstract A73).** The University of Dundee’s EDI governance structure

## A74 Reflections on designing and delivering equality, diversity, and inclusion training: key skills for York Chemistry undergraduate students, postgraduate students and graduate teaching assistants

### Leonie C Jones, Julia P Sarju

#### University of York, York, United Kingdom

##### **Correspondence:** Leonie C Jones (leonie.jones@york.ac.uk)

*BMC Proceedings 2023*, **17(15):**A74

Inequality exists in access, participation, and experience of higher education. Stereotypes about who can and cannot succeed in STEM persist, which puts additional burden on minoritised students. The Department of Chemistry delivers dedicated Equality, Diversity and Inclusion (EDI) training for undergraduates [1] and research students (Figure 1). Inclusive teaching and accessibility have also been incorporated into the Chemistry graduate teaching training [2].

This presentation will describe how training activities were developed and how they have expanded and evolved in response to the changing EDI landscape. The way the training connects and influences other key topics in the curriculum, for example resilience and employability, will be discussed. In addition, plans for adapting the course in response to changing student needs in the post-COVID–19 ‘new normal’ will be presented.

**References**

1. Jones LC, Sarju JP, Dessent CEH, Matharu AS, Smith DK. What makes a professional chemist? Embedding equality, diversity, and inclusion into chemistry skills training for undergraduates. J. Chem Educ. 2022;99(1):480-6.
2. Sarju JP, Jones LC. Improving the equity of undergraduate practical laboratory chemistry: incorporating inclusive teaching and accessibility awareness into chemistry graduate teaching assistant training. J. Chem Educ. 2022;99(1):487-93.

**Fig. 1 (abstract A74).**
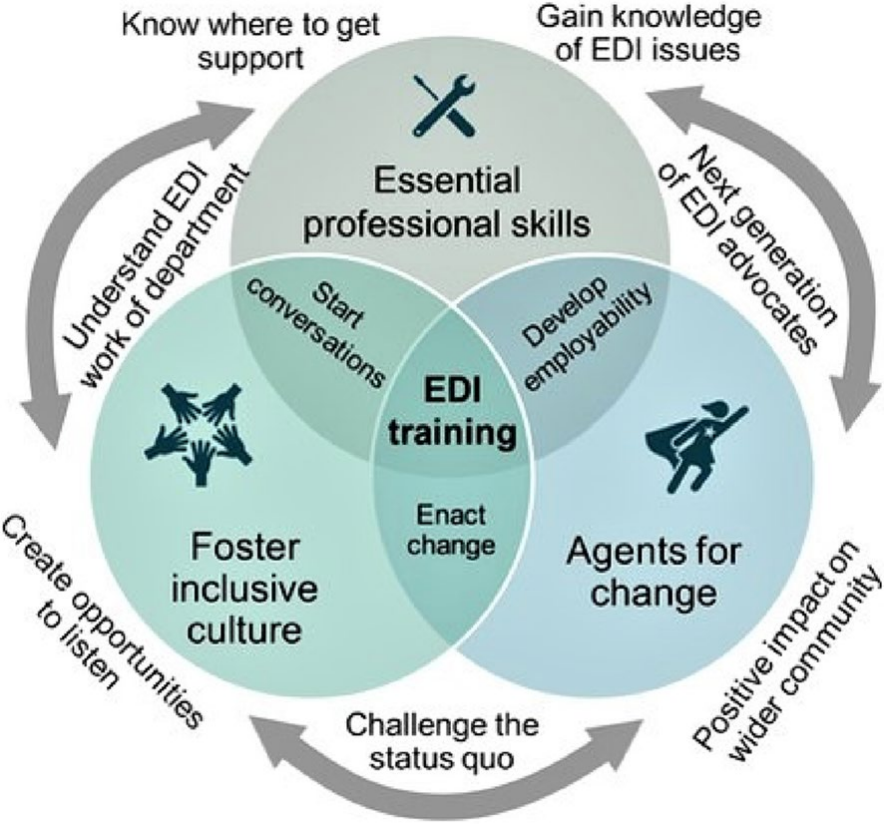
Key aims of EDI training

## A75 Cross-institutional ex-ante evaluation of gender equality plans' interventions

### Mustafa Özbilgin^1*^, Nur Gündoğdu^2^

#### ^1^Brunel University London, London, United Kingdom; ^2^University of Birmingham, Birmingham, United Kingdom

##### **Correspondence:** Mustafa Özbilgin (mustafa.ozbilgin@brunel.ac.uk)

*BMC Proceedings 2023*, **17(15):**A75

**Background**

This cross-institutional ex-ante evaluation aims to address the indented Gender Equality (GE) targets of each participating institution before developing their Gender Equality Plans (GEPs) and launching their interventions. As a part of action research, we examined data from five universities across the UK, Europe and the Middle East to show the direction of possible cross-institutional changes.

**Methods**

We created the ex-ante evaluation survey tool based on the indicators developed and agreed upon in the Evaluation Plan report [1] and applied it to participant institutions. The survey provided 77 qualitative and quantitative questions/indicators under 18 dimensions. These dimensions were derived from micro-, meso- and macro-level GE principles and indexes like Athena Swan [2], UN [3] and World Economic Forum [4]. Each institution has also access to the other partners’ responses. Accessing other partners’ responses facilitated cross-fertilisation of ideas and wider discussion of gender equality issues. We evaluated and summarised each participant's likelihood of GEP target settings for each broad parameter.

**Results**

The purpose of the institutional evaluation was to show the institutions where they were particularly focusing. Figure 1 demonstrates a cross-institutional picture of the ex-ante evaluation of GEPs' Interventions. We now provide a detailed account of how to cross-institutional evaluation is performed. If there are one or more targets indicated for an indicator in a parameter, we marked that parameter as active (green). If there are no targets set for indicators for a particular parameter, we marked it as inactive (red).

The overall evaluation shows institutionally unique arrangements with some commonality across some parameters. There are four dimensions in which all partners may have possible targets. These indicators are: (1) numerical representation, (2) careers, (3) human resources and (4) intersectionality. There is no category among the 18 categories that do not have a possible target. Some categories are less popular, with less than three institutions indicated as targets. These include (1) change, (2) curriculum, (3) impact, (4) values and (5) COVID-19 impact.

**Conclusions**

This evaluation focused on participating institutions’ intentions, and key concerns regarding their GEP development. Participant institutions are embedded in different regulatory and cultural systems have different states of play in terms of GE. As we had expected, this is reflected in this evaluation in terms of differences in their target choices and institutional arrangements.

**References**

1. Özbilgin M, Gundogdu N. Deliverable 5.1: TARGETED-MPI Evaluation Plan. 2021 Jun 21. 45 p. Supported by Horizon 2020.
2. Advance HE [Internet]. York: Advance HE. The transformed UK Athena Swan Charter. Available from: https://www.advance-he.ac.uk/equality-charters/transformed-uk-athena-swan-charter
3. Cohen SI, Sachdeva N, Taylor SJ, Cortes P. Gender mainstreaming approaches in development programming: being strategic and achieving results in an evolving development context. New York: UN Women; 2013 Oct 30. 59 p.
4. World Economic Forum. Global gender gap report 2020. Geneva: World Economic Forum; 2019 Dec 16. 370 p.

**Fig. 1 (abstract A75).**
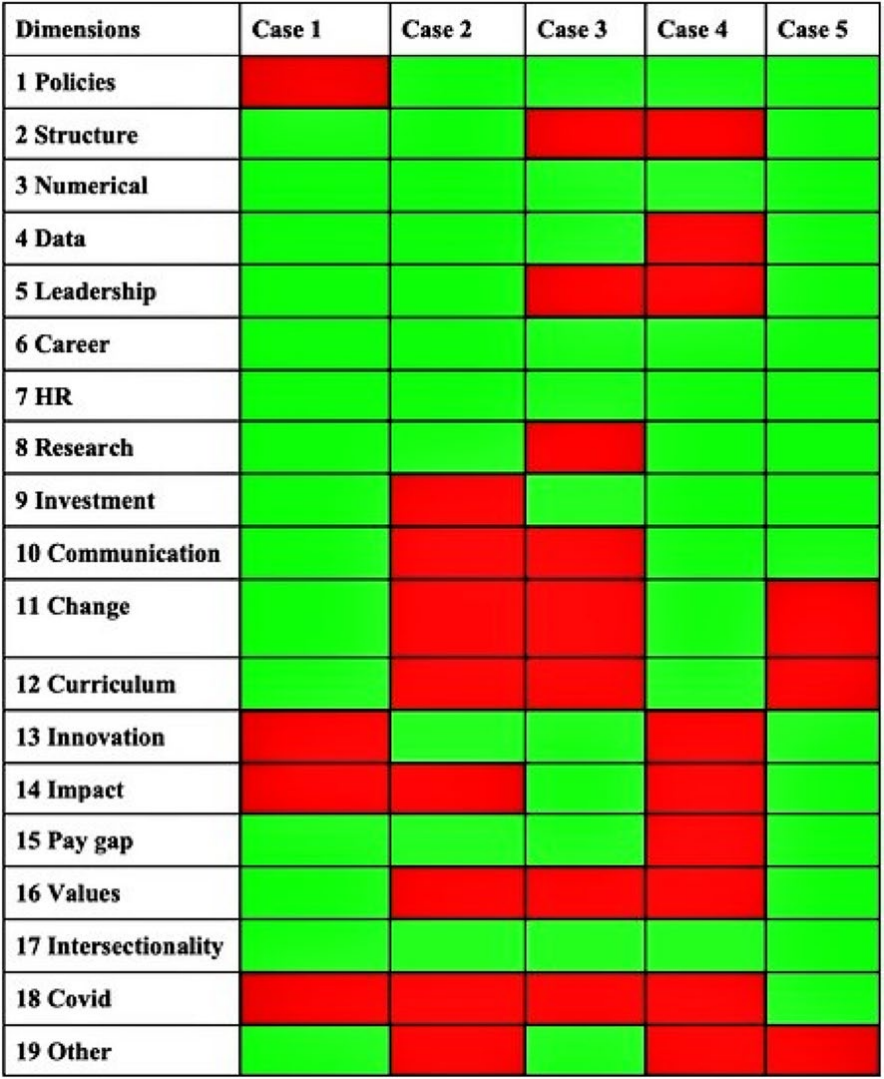
Cross-institutional ex-ante evaluation of Gender Equality Plans' interventions across 18 dimensions

## A76 Pathways to gender equality: experiences from Spain, Poland and Greece in the RESBIOS project

### Janire Salazar^1*^, Esther Garcés^1^, Evanthia Kalpazidou Schmidt^2^, Magdalena Żadkowska^3^, Natasza Kosakowska-Berezecka^3^, Izabela Raszczyk^3^, Marta Dziedzic^3^, Aglaia Pappa^4^, Alex Galanis^4^

#### ^1^Institut de Ciències del Mar, Barcelona, Spain; ^2^Aarhus University, Aarhus, Denmark; ^3^University of Gdańsk, Gdańsk, Poland; ^4^Department of Molecular Biology and Genetics Democtitus University of Greece, Alexandroupolis, Greece

##### **Correspondence:** Janire Salazar (jsalazar@icm.csic.es; janireslzr@gmail.com)

*BMC Proceedings 2023*, **17(15):**A76

Responsible Research and Innovation (RRI) [1] is a concept used by the European Commission, which implies that different societal actors (researchers, citizens, policy makers, businesses, etc.) work together during the research and innovation process with the objective of better aligning its outcomes with the values, needs and expectations of society. RRI connects different aspects of the relationship between research and innovation and society, e.g. public engagement, open access, gender equality, science education, ethics and governance [2]. The European project RESBIOS [3] (“Responsible Research in Biosciences”, 2020–2022) draws on the expertise of 12 partners from 11 countries to bring sustainable institutional changes based on RRI implementation in the field of bioscience.

In this presentation, the experience of three European institutions (based respectively in Spain, Poland and Greece) implementing institutional changes to achieve gender equality in their research institutions will be discussed. With the presentation of the three different grounding actions and pathways towards gender equality, we would like to inspire other research institutions to pursue institutional change through RRI practice. The success of these actions lies on the setting up of a mutual learning environment in the frame of the project, which connects RRI experienced partners acting as mentors with less experienced partners, newcomers in implementing RRI actions.

The RESBIOS grounding actions are structured in three different and consecutive strands, respectively aimed at: (i) developing a self-reflection on the change needs in the research institutions and dialoguing with the involved stakeholders for co-designing the grounding actions (co-reflexive strand); (ii) implementing the set of grounding actions in cooperation with societal actors in the territory and inside the research institutions (proactive strand); and (iii) developing permanent spaces of integration with society, thus practising institutional change inside the research organisation and reframing its governance settings (institutional strand).

**References**

1. RRI Tools [Internet]. What is RRI? Available from: https://rri-tools.eu/about-rri
2. Kalpazidou Schmidt E. Creating a developmental framework for evaluating RRI implementation in research organisations. Evaluation and Program Planning 2023;100:102350.
3. RESBIOS: Responsible Research in Biosciences [Internet]. Available from: https://resbios.eu

## A77 The Gender Equality Plan at University of Minho

### Emilia Araujo

#### University of Minho, Braga, Portugal

##### **Correspondence:** Emilia Araujo (emiliararaujo@gmail.com)

*BMC Proceedings 2023*, **17(15):**A77

The process of building and implementing gender equality plans in higher education institutions in Portugal has been rather slow. Until 2022, only a few institutions had gender equality plans approved and in operation. In most cases, these plans stemmed from research projects developed by researchers working in the institutions. There is still a large void in the specific field of science and technology in the country concerning gender equality issues. However, the country is undergoing significant changes largely driven by EU legislation that makes gender equality plans mandatory for access to EU funding. Furthermore, issues related to abuse and harassment in higher education institutions have moved also more frequently to the media stage, and universities have been investing in codes of ethics and also in internal processes of discussion of several measures.

In the case of the University of Minho (UMinho), the process started some years ago, also based on a research project [1], but it was only formalised this year with the elaboration of a three-year Gender Equality Plan based on a report taking into account the results of previous projects as well as data obtained from a survey among the teaching and research populations, students and non-academic staff [2].

The information allowed the establishment of some fundamental policy guidelines for the consolidation of the UMinho Gender Equality Plan [2]: participation, involvement and debate; awareness, debate and training on gender issues; study, diagnosis and monitoring; involvement of people of different genders at all decision levels. The following actions were defined on the basis of those guidelines:

1. Elaboration of UMinho's code of conduct concerning gender equality;
2. Establishment of the Commission for Gender Equality at UMinho (CIGUM);
3. Analysis and reorganisation of UMinho's internal information collection and dissemination procedures;
4. Development of training actions on gender issues in academy and science, with an interdisciplinary character and focusing on different audiences;
5. Training aimed at identifying unconscious gender bias among teachers, staff with management positions, non-academic staff and students, raising awareness of the issue among the various groups and audiences;
6. Promotion of gender balance in careers and teaching;
7. Promotion of measures to improve the conciliation of professional, family and personal life;
8. Elaboration of a protocol for forwarding complaints related to gender discrimination.

At this point, it is still too early to assess the impact of the equality plan. Even so, it can be argued that this is a lengthy process, which involves establishing many bridges between the various internal actors in order to consolidate a culture of equality and pluralism, free from stereotypes and biases.

**References**

1. Barros V, Ramos I. Gender equality at the University of Minho: empowering women for successful careers in engineering. Scienza e società. 2019;4:77-96.
2. Ferreira EC, Araújo ER, Ramos I, Gomes ME, Maciel P, Jerónimo P, Ramada JASS, Isaías M. Proposta de plano para a igualdade de género da Universidade do Minho (IGUM 2022-2024). Braga: University of Minho; 2022.

## A78 Addressing the attainment gap through Inclusive curriculum design and student data analysis

### Charis Kaur Pooni, Tasmina Islam, Helen Coulshed, Lucy Ward

#### King's College London, London, United Kingdom

##### **Correspondence:** Charis Kaur Pooni (charis.pooni@kcl.ac.uk)

*BMC Proceedings 2023*, **17(15):**A78

**Background**

The black, Asian and minority ethnic (BAME) attainment gap has been a long-standing issue across Higher Education (HE) institutions within the UK. The attainment gap is defined as the difference in the percentage of BAME and white students who achieve a 2:1 or a first, which has been found to be 13% for UK universities [1]. In this project, we aim to gain more insight to the attainment gap at the departmental level, by looking within the Natural, Mathematical and Engineering Sciences (NMES) faculty at King’s College London.

In addition, we are conducting a systematic literature review with recommendations for best practice for curriculum design, and possible causes of the attainment gap. We aim to bring our results and literature review to key stakeholders within the NMES faculty to highlight, address and close the attainment gap that exists.

**Methods**

We collect core first-year module assessment and demographic data for 19/20 and 20/21 from Chemistry, Engineering, Informatics, Mathematics and Physics departments in the NMES faculty, and calculate the attainment gap based on student ethnicity, fee status and gender. We maintain ethnicity sub-groups within our analysis, rather than calculating the BAME attainment gap by grouping the data, to gain as much resolution as possible, i.e. students with a lower level of attainment do not go unnoticed due to averaging [2].

Furthermore, we analyse the data in an intersectional way, using data about students’ gender, ethnicity and fee status. Intersectionality, first introduced by Kimberlé Crenshaw in 1989, describes the ways in which an individual’s experience of oppression is unique and can only be fully realised by considering how every mode of oppression (due to e.g. race, gender, class, disability etc.) interacts [3].

**Results**

From our ongoing literature review, we have found interesting examples of non-inclusive assessments such as true–false–abstain examinations [4]. We also highlight the importance of having a more flexible approach to assessments which could encourage engagement and academic achievement through a more personalised learning experience [5].

**Conclusions**

It is essential that we collect, process and analyse data over 3 to 5 years to improve the reliability of our recommendations. Through reviewing the awarding gap regularly, assessment type and student engagement, we aim to see a positive impact on the awarding gap for marginalised groups.

**References**

1. Universities UK and National Union of Students. Black, Asian and minority ethnic student attainment at UK universities: #ClosingTheGap. London: Universities UK and National Union of Students; 2019 May. 82 p.
2. Singh G. Black and minority ethnic (BME) students’ participation in higher education: improving retention and success: a synthesis of research evidence. London: Higher Education Academy; 2011 Jun 29. 73 p.
3. Crenshaw K. Demarginalizing the intersection of race and sex: a Black feminist critique of antidiscrimination doctrine, feminist theory and antiracist policies. Univ Chic Leg Forum. 1989;1989(1):139-67.
4. Kelly S, Dennick R. Evidence of gender bias in True-False-Abstain medical examinations. BMC Med Educ. 2009;9:32.
5. Higher Education Funding Council for England (HEFCE), Office for Fair Access (OFFA). National strategy for access and student success in higher education. London: Department for Business, Innovation and Skills; 2014 Apr. 113 p.

